# To Bond or Not to Bond: Metal–Metal Interaction in Heterobimetallic Rare-Earth Metal–Silver Complexes

**DOI:** 10.1021/acs.inorgchem.3c02377

**Published:** 2023-10-18

**Authors:** Alexandra Haidinger, Christina I. Dilly, Roland C. Fischer, Dennis Svatunek, Johanna M. Uher, Johann A. Hlina

**Affiliations:** † Institute of Chemistry, Inorganic Chemistry, University of Graz, Schubertstraße 1, 8010 Graz, Austria; ‡ Institute of Inorganic Chemistry, Graz University of Technology, Stremayrgasse 9, 8010 Graz, Austria; § Institute of Applied Synthetic Chemistry, TU Wien, Getreidemarkt 9, 1060 Vienna, Austria

## Abstract

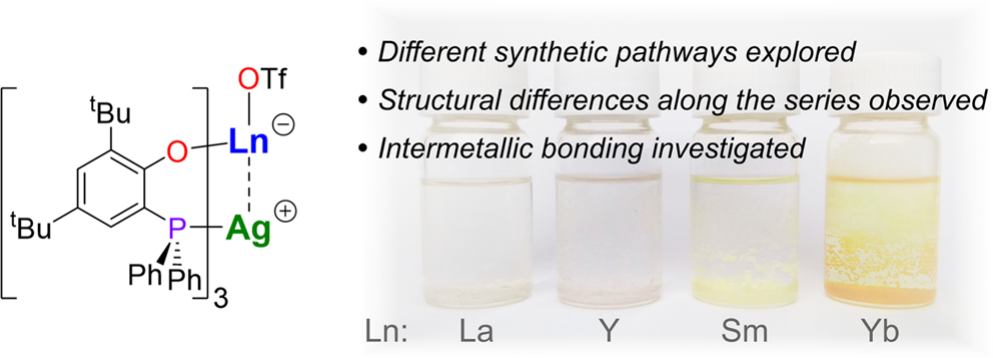

The reaction of 2,4-*^t^*Bu_2_-6-(PPh_2_)PhOH (HOAr^P^) with silver(I) triflate in a 3:1 molar ratio gave the mononuclear coinage metal complex (HOAr^P^-κ*P*)_3_Ag^I^OTf (**1**). Treatment of HOAr^P^ with Ln^III^[N(SiMe_3_)_2_]_3_ (Ln = La, Sm, Y, Yb) in a 3:1 molar ratio yielded the mononuclear rare-earth metal complexes Ln^III^(OAr^P^-κ^2^*O*,*P*)_3_ (**2-Ln**). The heterobimetallic rare-earth metal–silver complexes Ln^III^(OTf)(μ-OAr^P^-1κ^1^*O*,2κ^1^*P*)_3_Ag^I^ (**3-Ln**) were prepared from monometallic precursors by reactions of equimolar amounts of **1** with Ln^III^[N(SiMe_3_)_2_]_3_ or **2-Ln** with silver(I) triflate, respectively. The compounds were characterized by NMR, ultraviolet–visible (UV–vis), and infrared (IR) spectroscopy, single-crystal X-ray diffraction, elemental analysis, and the effective magnetic moments of the paramagnetic complexes were determined via the Evans NMR method. Computational studies were conducted on **3-La** and **3-Y**.

## Abstract 2

A series of heterobimetallic rare-earth metal(III)−silver(I) complexes were investigated for four different rare-earth metals (La, Sm, Yb, Y) also exploring two different synthetic pathways. Covering the ion radii range from lanthanum(III) to ytterbium(III), the solid-state structures of all four heterodinuclear complexes indicated the possibility of intermetallic bonding, which we further investigated, e.g., using computational methods.

## Introduction

**Figure 1:**
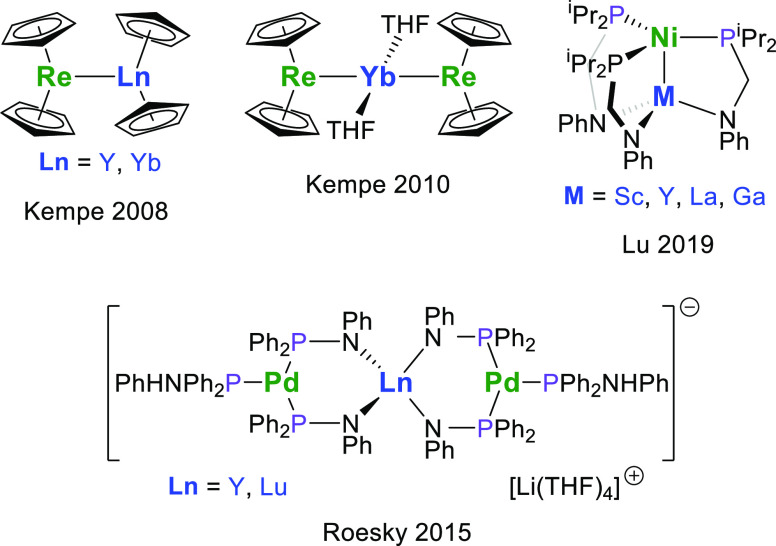
Examples of previously reported heterobimetallic rare-earth-transition metal complexes.^7,9,11,16^

Molecular bonds between transition metals have been known for several decades.^1,2^ Their study provides valuable insight into complex bonding situations and opportunities to challenge our theoretical understanding of bonding and refining our models. Intermetallic transition metal bonding opened up doors to reactivity not accessible to single-metal atoms and paved the way to catalytic applications beyond the capabilities of mononuclear catalysts.^3,4^ Although transition metals have been at the center of research for several decades, the investigation of metal–metal interactions involving f-block elements has received significantly less attention and examples are very few by comparison (Figure 1).^5,6^ Early examples of compounds featuring rare-earth-transition metal bonds were reported by the group of Kempe in which anionic metalloligands, such as bis(cyclopentadienyl)rhenate, Cp_2_Re^–^, act as ligands enabling intermetallic bonding in complexes such as Cp_2_ReLnCp_2_ (Ln = Y, Yb), (Cp_2_Re)_3_Ln (Ln = La, Sm, Lu), and (Cp_2_Re)_2_Yb(THF)_2_.^7−10^ Considering the limited stability of such metal–metal bonds, the use of ligand systems that provide support to keep the individual metal ions within proximity has been prominently explored in heterometallic transition metal chemistry and subsequently employed to realize heterometallic d-f-block metal complexes. Using this approach, the group of Roesky reported the di- and trinuclear rare-earth-group 10 metal complexes, in which phosphinoamide ligands provide the means to bind the different metal centers within proximity of each other.^11,12^ Later, Cui and co-workers also used similar ligand systems to explore intermetallic bonding and reactivity of rare-earth metal−palladium complexes.^13−15^ In another recent contribution, Lu and her group also used a phosphinoimide ligand system to support rare-earth metal−nickel bonding. In this case, the choice of the rare-earth metal or gallium allowed for tuning the reactivity of the complexes toward the catalytic hydrogenation of alkynes.^16^ Another prominent ligand system, which was already demonstrated to provide support for d-f-metal bonding, are phosphinoaryloxide compounds. In previous works on multinuclear complexes, the *o*-phosphinoaryloxide ligand system demonstrated its ability to support intermetallic bonding.^17−19^

In this work, we present our contribution to rare-earth-transition metal complexes, investigating a series of rare-earth metal–silver complexes.

## Results and Discussion

### Synthesis and Characterization

**Figure 2:**
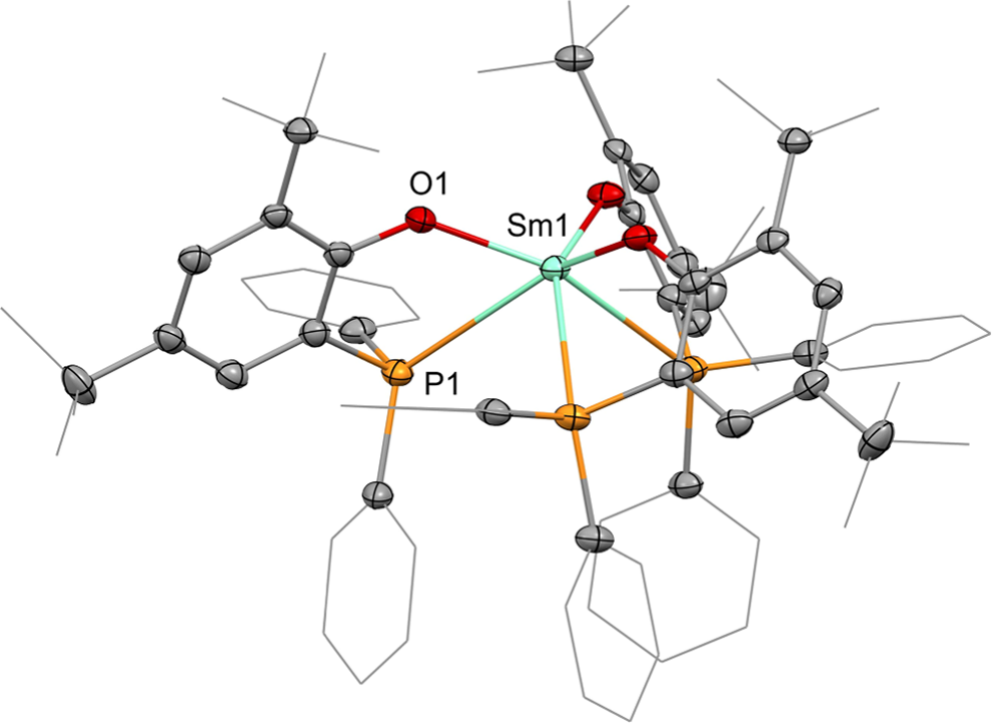
Molecular structure of **2-Sm**. Hydrogen atoms are omitted, and selected ligand carbon atoms are depicted as a wireframe for clarity. Thermal ellipsoids were drawn at 50% probability. Selected distances (Å) and angles (deg): Sm1–O1: 2.173(1), Sm1–P1: 3.1132(5), O1–Sm1–P1: 62.83(3), O1–Sm1–O1′: 108.56(3), P1–Sm1–P1′: 90.60(1).

**Figure 3:**
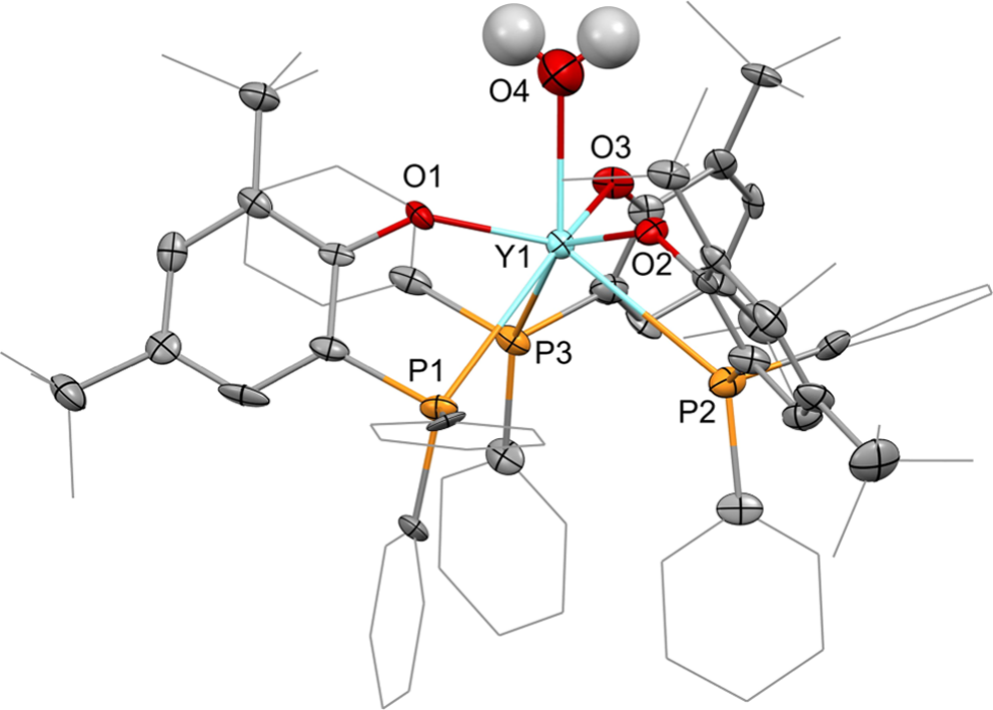
Molecular structure of **2-Y·H**_**2**_**O**. Hydrogen atoms, except for those of the water molecule, are omitted, and selected ligand carbon atoms are depicted as a wireframe for clarity. Thermal ellipsoids drawn at 50% probability. Selected distances (Å) and angles (deg): Y1–O1: 2.137(6), Y1–O2: 2.127(5), Y1–O3: 2.158(5), Y1–O4: 2.519(7), Y1–P1: 3.161(2), Y1–P2: 3.137(3), Y1–P3: 3.075(2), O1–Y1–P1: 62.0(2), O2–Y1–P2: 62.5(1), O3–Y1–P3: 62.4(2).

**Scheme 1:**
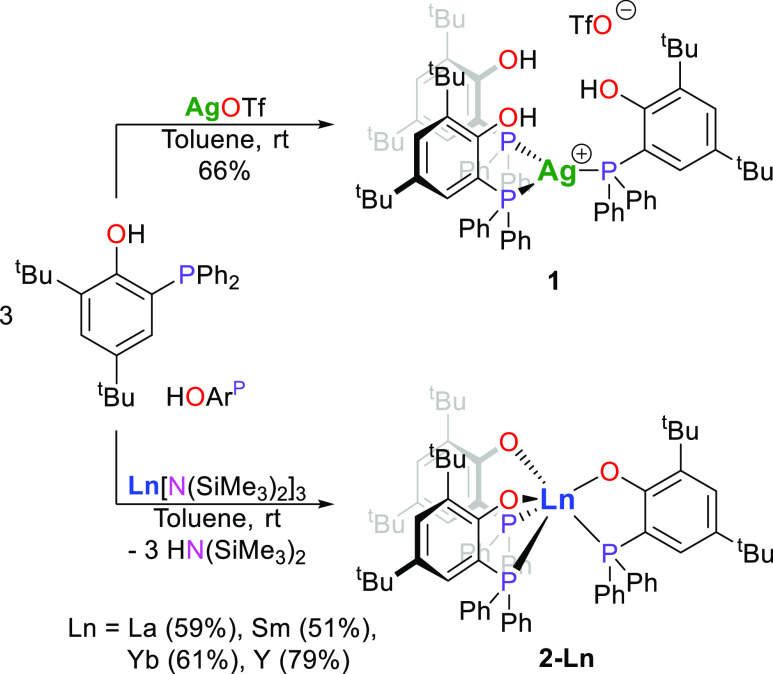
Synthesis of the Mononuclear Silver (**1**) and Rare-Earth Metal Complexes (**2-Ln**, Ln = La, Sm, Yb, Y)

The preparation of dinuclear rare-earth-coinage metal complexes, using 2,4-di-*tert*-butyl-6-(diphenylphosphanyl)phenol (HOAr^P^) as supporting ligand, may be approached by first introducing the coinage metal center followed by the rare-earth metal center or vice versa. In order to explore both synthetic pathways, we prepared the mononuclear silver complex (HOAr^P^-κ*P*)_3_Ag^I^OTf (**1**) by reaction of HOAr^P^ with silver(I) triflate in a 3:1 molar ratio in toluene at ambient temperature, yielding the compound **1** as colorless solids in 66% (Scheme 1). The NMR spectroscopic analysis of **1** exhibited a single set of resonances for the ligands with a ^31^P{^1^H} NMR shift at −0.5 ppm without any recognizable couplings to ^107^Ag and ^109^Ag. The synthesis of the mononuclear rare-earth metal complexes was performed by treatment of solutions of HOAr^P^ in pentane with solutions of Ln^III^[N(SiMe_3_)_2_]_3_ (Ln = La, Sm, Yb, Y) in the same solvent in 3:1 molar ratio at ambient temperature. The resulting rare-earth metal complexes, Ln^III^(OAr^P^-κ^2^*O*,*P*)_3_ (**2-Ln**, Ln = La, Sm, Y, Yb), precipitated from the reaction mixtures as colorless (**2-La** and **2-Y**), pale (**2-Sm**), and intense yellow (**2-Yb**) microcrystalline solids in yields of 51 to 79%. The ^1^H NMR spectra of compounds **2-Ln** show only single sets of resonances for the Ar^P^O^–^ ligand, which fits with the structures observed in the solid state (see Figure 2 for **2-Sm** and Figures S1–S3 in the Supporting Information for **2-La**, **2-Yb**, and **2-Y**). The ^1^H NMR signals of the paramagnetic compound **2-Yb** cover the range from −18.56 to 28.77 ppm, whereas for **2-Sm**, all resonances are observed within −0.51 and 10.13 ppm. The solid-state structures exhibit a difference in the orientation of the two phosphane-bound phenyl groups, one in apical and the other in equatorial orientation. In the ^1^H NMR spectra of the paramagnetic complexes **2-Sm** and **2-Yb**, the two phenyl groups show individual sets of signals, whereas in the diamagnetic complexes **2-La** and **2-Y**, they appear to overlap. We attribute this difference in behavior to the paramagnetic properties of Sm(III) and Yb(III), causing a distribution of the signals over a wider spectral range in comparison with diamagnetic La(III) and Y(III). The ^31^P{^1^H} NMR spectroscopic analysis for the diamagnetic lanthanum and yttrium complexes showed signals at −0.3 (**2-La**) and −9.7 ppm (**2-Y**). In the case of **2-Y**, the signal is observed as a doublet with a 61 Hz coupling constant due to the coupling to ^89^Y, indicating the coordination of the phosphane groups to the yttrium center. For the paramagnetic derivatives **2-Sm** and **2-Yb**, no ^31^P NMR resonances could be observed, and for the latter compound also, no ^13^C NMR data could be obtained. We calculated the effective magnetic moments μ_eff_ using the Evans NMR method of the paramagnetic compounds to 1.2 (**2-Sm**) and 4.0 μ_B_ (**2-Yb**), and the values are similar to those of the free ions.^20,21^ Crystallization of the rare-earth metal complexes from benzene/pentane yielded single crystals suitable for X-ray diffraction analysis. The two compounds with the larger rare-earth metal ions, **2-La** and **2-Sm**, crystallized in the trigonal space group *R*3̅, **2-Yb** in the monoclinic space group *P*2_1_/*c*, and **2-Y** in the triclinic space group *P*1̅. The solid-state structures of **2-Ln** show the rare-earth metal ions bound to the three Ar^P^O^–^ groups as bidentate ligands in distorted trigonal prismatic coordination (see Figure 2 for **2-Sm** and Figures S1–S3 in the Supporting Information for **2-La**, **2-Yb**, and **2-Y**). The Ln–O bond distances are observed in the range from 2.255(1) Å in **2-La** to 2.085(3)–2.101(3) Å in **2-Yb** following the decrease of the ionic radii of the rare-earth metal 3+ ions. Similarly, the Ln–P distances span from 3.2214(5) Å in **2-La** to 2.9112(8)–2.9635(9) Å in **2-Yb**. This results in O–Ln–P bite-angles in the range of 68.24(8)–69.23(8)° in **2-Yb** to 60.05(4)° in **2-La**. In the first attempt to collect data for **2-Y**, we serendipitously encountered the hydrate of **2-Y** (Figure 3). We assume that this adduct is the result of trace water present in the toluene used for crystallization in this case. Considering the sensitivity of **2-Ln** to hydrolysis, we believe **2-Y·H**_**2**_**O** to represent an intermediate of this process. Due to the limited quality of the data, we are hesitant to discuss the structural properties of **2-Y·H**_**2**_**O** in detail and limit it to a few selected parameters. The distance of the water oxygen atom to the yttrium ion is 2.519(7) Å. The phenolate oxygen atoms are coordinated in the range of 2.127(5) to 2.158(5) Å and, therefore, slightly elongated in comparison to the Y–O bond distances, 2.116(2)–2.128(3) Å, in the molecular structure of the unhydrated **2-Y** complex. Similarly, the Y–P distances in **2-Y·H**_**2**_**O**, 3.075(2)–3.261(2) Å, are also longer than in the water-free congener **2-Y**, 2.967(1)–3.008(1) Å. The slight elongation of the other coordinated ligand atoms fits with the presence of an additional donor atom bound to the yttrium center. Hydrates of yttrium(III) phenolate complexes have previously been reported, for instance, using a phenol-based cryptate ligand.^22^

**Scheme 2:**
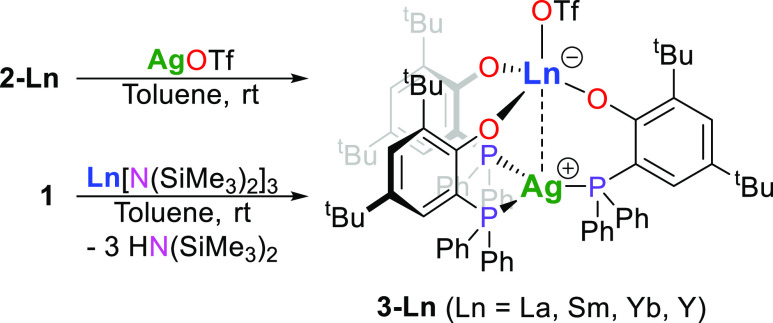
Synthesis of the Dinuclear Rare-Earth Metal–Silver Complexes (**3-Ln**)

For the incorporation of the rare-earth metal center to yield Ln^III^(OTf)(μ-OAr^P^-1κ^1^*O*,2κ^1^*P*)_3_Ag^I^ (**3-Ln**, Ln = La, Sm, Yb, Y), we treated **1** with equimolar amounts of Ln^III^[N(SiMe_3_)_2_]_3_ (Ln = La, Sm, Yb, Y) using toluene or THF at ambient or elevated temperatures of up to 60 °C (Scheme 2). However, the reactions gave only impure products and unsatisfying yields after purification by washing with pentane of typically less than 20%. When performed at ambient temperature, the reactions exhibited remaining rare-earth metal-bound hexamethyldisilazide groups. Therefore, we changed the synthetic approach to the alternative pathway and introduced the rare-earth metal prior to silver into the compounds. The reaction of **2-Ln** (Ln = La, Sm, Yb, Y) with equimolar amounts of silver(I) triflate in toluene or THF gave improved yields of isolated **3-Ln** to a range of 55 to 68% for the series (Scheme 2). The complexes were isolated as colorless (**3-La** and **3-Y**), pale (**3-Sm**), and intense yellow (**3-Yb**) microcrystalline powders. Attempts to exchange the triflate group in **3-La** or **3-Y** for hexamethyldisilazide, by reaction with equimolar amounts of NaN(SiMe_3_)_2_ in THF or toluene at ambient temperature repeatedly, gave inseparable mixtures of corresponding **2-Ln** and [AgN(SiMe_3_)_2_]_4_ along with trace amounts of a complex, which we presume to be the desired dinuclear compounds.^23^ Other attempts to exchange the triflate group in the aforementioned complexes for benzyl or methyl groups using benzylpotassium or methyllithium, respectively, in THF or toluene at ambient temperature resulted in decomposition under deposition of metallic silver typically within the hour.

The low solubility of the range of **3-Ln** complexes inhibited the observation of the entire set of expected ^13^C{^1^H} signals, and in case of the paramagnetic complex **3-Yb**, no reliable data could be recorded within a reasonable measurement period. The ^31^P{^1^H} NMR spectra of the dinuclear complexes show the resonances of the silver-coordinated phosphanes equivalent with very similar signals for the lanthanum, samarium, and yttrium derivatives at −0.5 (**3-La**), −1.0 (**3-Sm**), and −1.3 ppm (**3-Y**). Only the ytterbium derivative (**3-Yb**) exhibits a strongly paramagnetically shifted signal at 54.0 ppm. In contrast to mononuclear silver complex **1**, well-resolved couplings of phosphorus to silver are observed for dinuclear complexes **3-La**, **3-Sm**, and **3-Y** with 325 Hz to ^107^Ag and 375 Hz to ^109^Ag in all three complexes. Only in the case of **3-Yb** are the individual couplings between ^31^P and the two different silver nuclei, ^107^Ag and ^109^Ag, indistinguishably merged into a single broadened doublet with an averaged coupling constant of 342 Hz. Considering that we observed a ^1^*J*-coupling between ^89^Y and ^31^P in **2-Y**, we took a closer look at the ^31^P{^1^H} NMR data for **3-Y**; however, there is no recognizable ^2^*J*-coupling between ^89^Y and ^31^P, which may be conveyed by the ^107/109^Ag nucleus. The observed coupling constants are within the wide range of previously reported values.^24^ Using the Evans NMR method also for the two paramagnetic dinuclear complexes **3-Sm** and **3-Yb**, the effective magnetic moments were calculated to be 1.2 and 4.7 μ_B_, respectively. For the samarium complex, we encounter no change of the effective magnetic moment upon introduction of the silver ion into the complex, but for the ytterbium derivative, we observed an increase in comparison to the parent mononuclear compound, which is still similar to that of the free ion.^21^

**Figure 4:**
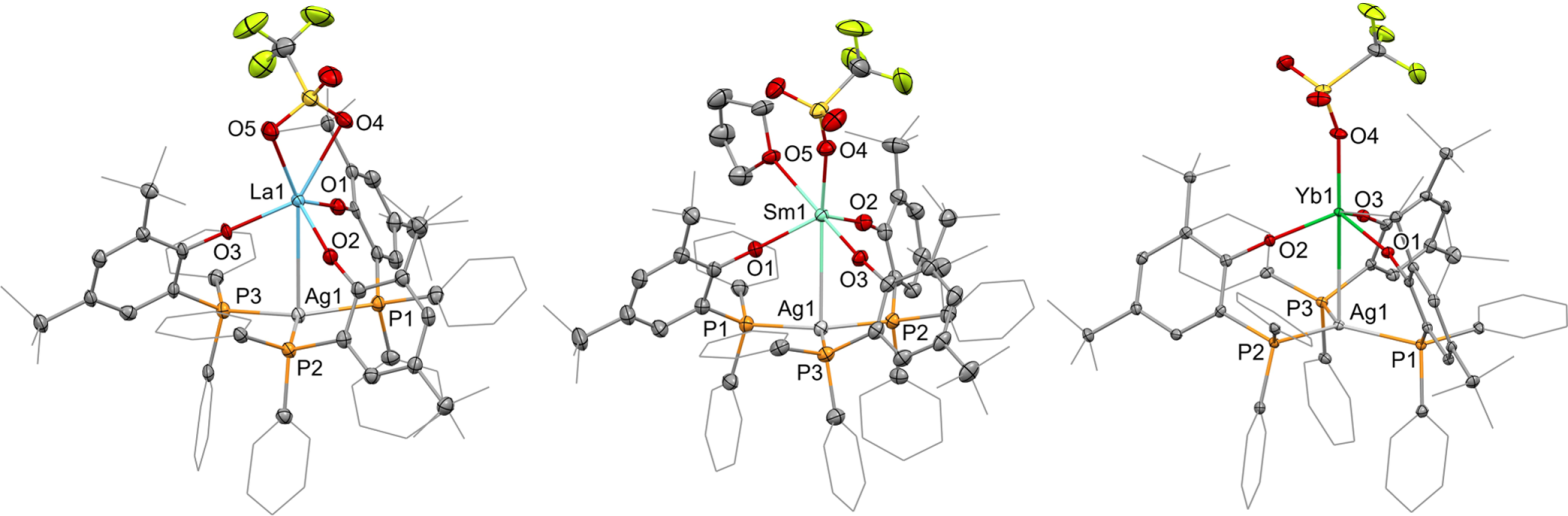
Molecular structure of **3-La** (left), **3-Sm** (middle), and **3-Yb** (right). Hydrogen atoms are omitted, and selected ligand carbon atoms are depicted as a wireframe for clarity. Thermal ellipsoids drawn at 50% probability. Selected distances (Å) and angles (deg): **3-La**: La1–Ag1: 3.4462(8), La1–O1: 2.232(2), La1–O2: 2.221(2), La1–O3: 2.250(1), La1–O4: 2.633(2), La1–O5: 2.710(2), La1–Ag1–P1: 92.82(2), La1–Ag1–P2: 89.81(2), La1–Ag1–P3: 98.86(2). **3-Sm**: Sm1–Ag1: 3.3660(8), Sm1–O1: 2.173(2), Sm1–O2: 2.184(2) Sm1–O3: 2.177(2), Sm1–O4: 2.356(2), Sm1–O5: 2.547(2), Sm1–Ag1–P1: 93.34(2), Sm1–Ag1–P2: 91.01(2), Sm1–Ag1–P3: 95.59(2). **3-Yb**: Yb1–Ag1: 3.2178(5), Yb1–O1: 2.058(2), Yb1–O2: 2.059(2), Yb1–O3: 2.047(2), Yb1–O4: 2.198(2), Yb1–Ag1–P1: 96.72(2), Yb1–Ag1–P2: 90.06(2), Yb1–Ag1–P3: 91.07(2).

The crystallization of the dinuclear complexes from THF/pentane or benzene/pentane at ambient temperature afforded the crystalline material suitable for single-crystal X-ray diffraction analysis. The dinuclear **3-Ln** complexes crystallized in the *P*2_1_/*c* (Ln = La, Y, Yb) and *P*2_1_/*n* (Ln = Sm) space groups and exhibit the silver cation coordinated by the three phosphorus atoms and the rare-earth metal ion bound to the three phenolates and the triflate via the oxygen atoms (Figures 4 and S4 in the Supporting Information). A distinct difference between the four complexes is the coordination mode of the triflate to the rare-earth metal center, which is bound via two oxygen atoms in **3-La**, which in this case was crystallized from benzene/pentane and via only one oxygen atom in **3-Sm**, **3-Yb**, and **3-Y**, whereas in **3-Sm**, the samarium atom is additionally coordinated by a THF molecule. We presume this to be a result of the larger ionic radii of lanthanum(III) and samarium(III) in comparison with ytterbium(III) and yttrium(III), through which the former provides a larger area available for coordination of the second oxygen donor in the form of a triflate oxygen atom (**3-La**) or a THF ligand (**3-Sm**).^25^ The most striking features of the dinuclear complexes are the Ln–Ag distances, which are 3.4463(8) Å (**3-La**), 3.3659(5) Å (**3-Sm**), 3.2725(7) Å (**3-Y**), and 3.2178(5) Å (**3-Yb**). The intermetallic distances are shorter than the sums of the covalent radii of the respective metals, 3.53 (**3-La**), 3.43 (**3-Sm**), 3.35 (**3-Y**), and 3.32 Å (**3-Yb**), and the formal shortness ratios, the quotients of intermetallic distances and the sums of the respective covalent radii, are in range of 0.97–0.98.^26^ The availability of a filled 4d(*z*^2^) orbital on silver(I), a situation that enabled dative uranium(IV)–nickel(0) bonding using a very similar ligand system, suggests the possibility of dative Ln–Ag bonding.^17^ Moreover, a closer look on the coordination of the silver atom by the three phosphanes shows the Ln–Ag–P angles are in range of 89.80(3)–98.87(3)° (**3-La**), 91.00(1)–95.59(2)° (**3-Sm**), 90.18(3)–96.89(3)° (**3-Y**), and 90.06(2)–96.72(2)° (**3-Yb**). Therefore, in all four cases, the silver atom is slightly above the plane formed by the phosphorus atoms and leaning toward the rare-earth metal center.

### Optical Spectroscopy

The UV–vis spectra, recorded from solutions of the compounds in toluene, exhibit a single absorption band for mononuclear silver complex **1** rising to the cutoff at 300 nm with a molar extinction coefficient of 6.2 × 10^3^ Lmol^–1^cm^–1^ (see Figures S28 in the Supporting Information). This contrasts the mononuclear rare-earth metal complexes **2-Ln**, which show the absorption bands of the aromatic ligand within the narrow range of 327 (Ln = La, Sm) to 329 nm (Ln = Yb, Y) with molar extinction coefficients in the range of 1.2 × 10^4^ to 1.4 × 10^4^ Lmol^–1^cm^–1^ (see Figures S29–S32 in the Supporting Information). Similarly, the UV–vis absorption behavior of the dinuclear complexes **3-Ln** exhibits intense bands in the range of 305 (1.7 × 10^4^ Lmol^–1^cm^–1^, **3-Yb**) to 310 nm (1.5 × 10^4^ Lmol^–1^cm^–1^, **3-La**) (see Figures S33–S36 in the Supporting Information). Accordingly, we attribute these absorption bands to π–π* transitions in the ligand. The samarium compounds show weak absorptions, which may be assigned to f-f transitions from the ground-state ^6^*H*_5/2_ to ^4^*L*_13/2_ (410 nm), (^6^*P*,^4^*P*)_5/2_ (415 nm), ^4^*M*_19/2_ (420 nm), and ^4^*I*_15/2_ (429 nm) in **2-Sm** and to ^4^*H*_7/2_ (351 nm), ^4^*D*_1/2_ (382 nm), ^4^*M*_19/2_ (420 nm), and ^4^*L*_13/2_ (411 nm) in **3-Sm**.^27^ The two ytterbium complexes exhibit additional absorptions at 401 nm (3.2 × 10^2^ Lmol^–1^cm^–1^) in monometallic **2-Yb** and at 415 nm (2.6 × 10^2^ Lmol^–1^cm^–1^) in dinuclear **3-Yb**, which we attribute to ligand-to-metal charge transfer processes.^28,29^

The IR spectra of the silver complex **1** show the presence of the hydroxyl groups on the ligand with the broadband at 3468 cm^–1^, which is expectedly absent in the rare-earth metal complexes. The δ_as_ and δ_s_ vibrations for the *tert*-butyl groups are located at 1478 and 1437 cm^–1^, respectively, with the latter being significantly weaker than in **2-Ln** and **3-Ln**, where it is observed as part of double bands characteristic for *tert*-butyl groups in the range from 1420 to 1433 cm^–1^.^30^ We attribute the stronger bands to a lower degree of freedom of movement of the ligands in the rare-earth metal compounds compared to **1**. Due to the presence of numerous additional bands in the IR spectra of the **3-Ln** complexes, we cannot assign bands to vibrations of the SO_3_ unit of the triflate, especially in the region from 1000 to 1300 cm^–1^ for which corresponding stretching vibrations have been reported, and unambiguously determine the binding mode to the rare-earth metal ions in addition to the data from single-crystal X-ray diffraction analysis.^31^

### Computational Studies

To analyze intermetallic bonding in **3-La** and **3-Y**, localized molecular orbitals (LMOs) were calculated by using the Pipek–Mezey and Foster–Boys method. LMOs are commonly used to analyze bonding in metal complexes and metal clusters.^32−36^ In all cases, no bonds between the silver atom and La and Y could be located. For **3-Y**, an additional Intrinsic Bond Orbital (IBO) analysis was performed, which also showed no bond between the two metal atoms. Fuzzy Bond Order analysis revealed low bond orders of 0.36 and 0.35 for Y–Ag and La–Ag, respectively. Therefore, we conclude that there is only a weak covalent interaction between the metal atoms in either complex.

## Conclusions

In summary, we presented the synthesis and characterization of mononuclear complexes (HOAr^P^-κ*P*)_3_Ag^I^OTf (**1**) and Ln^III^(OAr^P^-κ^2^*O*,*P*)_3_ (**2-Ln**, Ln = La, Sm, Y, Yb) and explored their utility to prepare the dinuclear rare-earth metal–silver complexes Ln^III^(OTf)(μ-OAr^P^-1κ^1^*O*,2κ^1^*P*)_3_Ag^I^ (**3-Ln**, Ln = La, Sm, Y, Yb). Here, we found the approach to start from the mononuclear rare-earth metal complexes and introduce the silver center as the second metal to be the superior synthetic pathway. In the solid state, the dinuclear rare-earth metal–silver complexes **3-Ln** exhibit intermetallic distances within the sums of the covalent radii, suggesting the possibility of metal–metal bonding. In order to further clarify the situation, computational studies based on these solid-state structures were performed on **3-La** and **3-Y**, and despite the close proximity of the two metal centers, the calculations indicate that there is only a weak interaction between the rare-earth and silver atoms. Considering the intramolecular charge separation between the rare-earth metal atom, which is embedded as an *ate*-fragment, and the phosphane-coordinated silver cation, we conclude that the metal–metal interaction is of primarily electrostatic nature.

## Experimental Section

### General Details

All manipulations were carried out under an atmosphere of dry, oxygen-free nitrogen using standard Schlenk and glovebox techniques. Benzene-*d*_6_ was distilled from potassium. THF was purified by distillation from calcium hydride under nitrogen. All other solvents were purified by passing through columns of activated alumina.^37^ Ln^III^[N(SiMe_3_)_2_]_3_ (Ln = La, Sm, Yb, Y)^38^ and 2,4-*^t^*Bu_2_-6-(PPh_2_)PhOH^39^ were prepared according to published procedures. Other chemicals were obtained from different suppliers and used without further purification. The NMR spectra were recorded on a Varian INOVA 500 or Bruker AVANCE III 300 instrument and are referenced to Me_4_Si (^1^H, ^13^C). The ^1^H NMR data required for the Evans NMR method calculations of the effective magnetic moments, μ_eff_, were recorded using solutions of the analytes in benzene-*d*_6_ with and without a sealed capillary containing benzene-*d*_6_ to determine the shift differences between the solvent residue peaks. The shift differences of the resonances were then used for the calculations.^20,40^ For X-ray structure analyses, the crystals were mounted onto the tips of glass fibers. Data collection was performed with a Bruker-AXS SMART APEX CCD diffractometer using graphite-monochromated Mo Kα radiation (0.71073 Å) or a Bruker APEX II diffractometer using an Incoatec microfocus sealed tube of Mo Kα radiation (0.71073 Å) with a CCD area detector. The data were reduced to F_o_^2^ and corrected for absorption effects with SAINT^41^ and SADABS^42,43^ respectively. The structures were solved by direct methods and refined by full-matrix least-squares method (SHELXL97 or SHELXL19).^44^ If not noted otherwise, all non-hydrogen atoms were refined with anisotropic displacement parameters. All hydrogen atoms were located in calculated positions to correspond to the standard bond lengths and angles. Computational investigations were performed in ORCA 5.0.3^45^ using PBE-D3 in combination with the SARC-ZORA-TZVPP^46,47^ basis set on La, Y, and Ag, and ZORA-def2-SVP^48^ for all other atoms using geometries from the solid-state structures of **3-La** and **3-Y**. The zeroth order regular approximation (ZORA)^49^ was used to include relativistic effects. Bonding analysis was performed using Pipek–Mezey^50^ and Foster–Boys^51^ localized molecular orbitals. The “New-BOYS” algorithm in the ORCA was used to calculate Foster–Boys orbitals. Intrinsic bond orbitals,^52^ which are a popular tool to evaluate chemical bonding,^53−55^ were also evaluated for **3-Y**, but due to technical constraints, they are not available for lanthanides. Detailed input and output sections can be found in Supporting Information. MultiWFN 3.8^56^ was used for Fuzzy Bond Order analysis (FBO).^57^ UV–vis spectra were recorded on an Agilent Cary 60 UV–vis spectrophotometer using toluene to prepare the analyte solutions for the measurements. Elemental analysis was carried out using a Heraeus VARIO ELEMENTAR instrument and could only be performed for compounds free of fluorine. IR data were recorded on a Bruker α-T attenuated total reflectance-Fourier transform infrared (ATR-FTIR) spectrometer.

### Syntheses

#### Tris[2,4-di-*tert*-butyl-6-(diphenylphosphanyl)phenol]silver(I) Triflate (HOAr^P^-κ*P*)_3_Ag^I^OTf **1**

A scintillation vial equipped with a magnetic stir bar was charged with Ag^I^OTf (52 mg, 200 μmol) and toluene (5 mL). Then a solution of 2,4-di-*tert*-butyl-6-(diphenylphosphanyl)phenol (235 mg, 600 μmol) in toluene (5 mL) was added dropwise under stirring. The reaction mixture was stirred for 18 h at ambient temperature. After that the solvent was evaporated, and the residue was washed with pentane. The product was dried under vacuum and isolated as a colorless microcrystalline solid (189 mg, 66%). mp. 192–195 °C. ^1^H NMR (δ in ppm, benzene-*d*_6_, 298 K): 1.11 (s, 27H, ^*t*^Bu), 1.49 (s, 27H, ^*t*^Bu), 6.73 (dd, 3H, ^3^*J*_(1)H(31)P_ = 7 Hz, ^4^*J*_(1)H(1)H_ = 2 Hz, Ar*H*), 7.00 (s, 21H, Ar*H*), 7.27 (br s, 12H, Ar*H*), 7.59 (d, 3H, ^4^*J*_(1)H(1)H_ = 2 Hz, Ar*H*). ^13^C{^1^H} NMR (δ in ppm, benzene-*d*_6_, 298 K): 30.4, 31.4, 34.7, 35.1, 120.4, 120.9, 127.5, 128.6, 129.0, 130.6, 130.8, 131.1, 134.3 (d, ^1^*J*_(13)C(31)P_ = 17 Hz), 139.9 (d, ^2^*J*_(13)C(31)P_ = 3 Hz), 144.3 (d, ^2^*J*_(13)C(31)P_ = 7 Hz), 155.0 (d, ^2^*J*_(13)C(31)P_ = 10 Hz). ^31^P{^1^H} NMR (δ ppm, benzene-*d*_6_): −0.5. UV–vis (λ nm, ε Lmol^–1^cm^–1^ in brackets): 300 (6.2 × 10^3^). IR (ATR, cm^–1^): 400, 423, 464, 503, 579, 629, 694, 746, 751, 820, 887, 918, 1004, 1021, 1101, 1145, 1179, 1197, 1220, 1235, 1273, 1291, 1361, 1366, 1437, 1478, 2868, 2912, 2960, 3468.

#### Lanthanum(III)tris[2,4-di-*tert*-butyl-6-(diphenylphosphan-yl)phenolate] La^III^(OAr^P^-κ^2^*O*,*P*)_3_ (**2-La**)

A solution of La[N(SiMe_3_)_2_]_3_ (977 mg, 1.57 mmol, 1.05 equiv) in pentane (10 mL) was added to a stirred solution of HOAr^P^ (1.76 g, 4.5 mmol, 3.00 equiv) in 8 mL of the same solvent. During the addition, a white precipitate formed. After stirring for another 2 h, the clear solution was separated, and the white precipitate was washed with pentane (2 × 5 mL). Drying under reduced pressure gave **2-La** as white powder (1.15 g, 59%) and was used without further purification. The complex may be recrystallized from benzene/pentane. mp 225–229 °C. ^1^H NMR (δ ppm, benzene-*d*_6_): 1.25 (s, 27H, ^*t*^Bu), 1.52 (s, 27H, ^*t*^Bu), 6.80–7.02 (m, 21H, Ar*H*), 7.31 (s, 12H, Ar*H*), 7.57 (d, ^4^*J*_(1)H(1)H_ = 2 Hz, 3H, Ar*H*). ^13^C NMR (δ ppm, benzene-*d*_6_): 30.0, 32.0, 34.5, 35.5, 122.6 (d, ^1^*J*_(13)C(31)P_ = 26 Hz), 126.9, 129.0, 129.3, 133.9 (d, ^2^*J*_(13)C(31)P_ = 11 Hz), 134.4, 136.8, 139.1, 167.9. ^31^P{^1^H} NMR (δ ppm, benzene-*d*_6_): −0.3. UV–vis (λ nm, ε Lmol^–1^cm^–1^ in brackets): 327 (1.2 × 10^4^). IR (ATR, cm^–1^): 374, 419, 439, 497, 524, 574, 629, 693, 743, 777, 832, 857, 886, 915, 998, 1025, 1069, 1091, 1108, 1141, 1197, 1253, 1275, 1359, 1386, 1421, 1460, 1477, 1586, 2865, 2901, 2950, 3054. Analysis calculated for C_78_H_90_LaO_3_P_3_: C 71.66. H 6.94. Found: C 71.61. H 6.87.

#### Samarium(III)tris[2,4-di-*tert*-butyl-6-(diphenylphosphan-yl)phenolate] Sm^III^(OAr^P^-κ^2^*O*,*P*)_3_ (**2-Sm**)

**2-Sm** was prepared in the same way as **2-La** using Sm^III^[N(SiMe_3_)_2_]_3_ (959 mg, 1.52 mmol, 1.00 equiv) and HOAr^P^ (1.78 g, 4.55 mmol, 3.00 equiv). **2-Sm** was isolated as a pale yellow microcrystalline solid (1.02 g, 51%). The complex may be recrystallized from benzene/pentane. mp 233–235 °C. ^1^H NMR (δ ppm, benzene-*d*_6_): 0.51 (vbr s, ^*t*^Bu), 1.83 (s, 27H, ^*t*^Bu), 3.96 (vbr s, Ar*H*), 5.53 (vbr s, Ar*H*), 6.00 (vbr s, Ar*H*), 7.16 (s, 3H, Ar*H*), 7.74 (vbr s, Ar*H*), 8.43 (vbr s, Ar*H*), 8.54 (s, 3H). 10.13 (vbr s, Ar*H*). ^13^C NMR (δ ppm, benzene-*d*_6_): 29.6, 32.5, 35.5, 36.7, 128.9, 129.5, 132.5 (br), 136.7, 140.7, 181.6. μ_eff_ = 1.2 μ_B_. UV–vis (λ nm, ε Lmol^–1^cm^–1^ in brackets): 327 (1.2 × 10^4^), 410 (55), 415 (20), 420 (12), 429 (14). IR (ATR, cm^–1^): 374, 421, 445, 499, 526, 573, 631, 693, 744, 777, 835, 886, 915, 998, 1026, 1069, 1091, 1108, 1139, 1199, 1254, 1284, 1359, 1386, 1421, 1460, 1477, 1587, 2868, 2902, 2949, 3057. Analysis calculated for C_78_H_90_O_3_P_3_Sm: C 71.04. H 6.88. Found: C 71.26. H 7.11.

#### Ytterbium(III)tris[2,4-di-*tert*-butyl-6-(diphenylphosphan-yl)phenolate] Yb^III^(OAr^P^-κ^2^*O*,*P*)_3_ (**2-Yb**)

**2-Yb** was prepared in the same way as **2-La** using Yb^III^[N(SiMe_3_)_2_]_3_ (1.03 g, 1.58 mmol, 1.05 equiv) and HOAr^P^ (1.76 g, 4.5 mmol, 3.00 equiv). **2-Yb** was isolated as a white powder (1.22 g, 61%). The complex may be recrystallized from benzene/pentane. Mp 239–242 °C. ^1^H NMR (δ ppm, benzene-*d*_6_): −18.56 (s, 3H), −10.04 (s, 6H), −8.85 (s, 27H, *^t^*Bu), −7.71 (s, 3H), −5.76 (s, 3H), −4.33 (s, 3H), 24.44 (s, 3H), 27.55 (s, 27H, *^t^*Bu), 28.77 (s, 6H). μ_eff_ = 4.0 μ_B_. UV–vis (λ nm, ε Lmol^–1^cm^–1^ in brackets): 329 (1.4 × 10^4^), 401 (3.2 × 10^2^, sh). IR (ATR, cm^–1^): 377, 432, 451, 462, 496, 531, 571, 631, 693, 741, 775, 836, 856, 883, 915, 998, 1026, 1044, 1095, 1108, 1138, 1196, 1251, 1285, 1359, 1383, 1423, 1458, 1477, 1587, 2865, 2905, 2949, 3054. Analysis calculated for C_78_H_90_O_3_P_3_Yb: C 69.83. H 6.76. Found: C 68.59. H 6.60.

#### Yttrium(III)tris[2,4-di-*tert*-butyl-6-(diphenylphosphanyl)-phenolate] Y^III^(OAr^P^-κ^2^*O*,*P*)_3_ (**2-Y**)

**2-Y** was prepared in the same way as **2-La** using Y^III^[N(SiMe_3_)_2_]_3_ (941 mg, 1.65 mmol, 1.10 equiv) and HOAr^P^ (1.76 g, 4.50 mmol, 3.00 equiv). **2-Y** was isolated as a white powder (1.50 g, 79%). The complex may be recrystallized from benzene/pentane. Mp 245–247 °C. ^1^H NMR (δ ppm, benzene-*d*_6_): −1.20 (s, 27H, ^*t*^Bu), −1.67 (s, 27H, ^*t*^Bu), 6.71–6.98 (m, 24H, Ph*H*), 7.10 (s, 3H), 7.40 (s, 6H), 7.60 (d, ^4^*J*_(1)H(1)H_ = 3 Hz, 3H, Ar*H*). ^13^C NMR (δ ppm, benzene-*d*_6_): 29.8, 31.9, 34.5, 35.7, 121.9 (dd, ^1^*J*_(13)C(31)P_ = 21 Hz, ^2^*J*_(13)C(89)Y_ = 11 Hz),127.4, 128.7, 129.5, 133.5, 133.9, 137.7, 139.5, 167.6. ^31^P{^1^H} NMR (δ ppm, benzene-*d*_6_): −9.7 (d, ^1^*J*_(31)P(89)Y_ = 61 Hz). UV–vis (λ nm, ε Lmol^–1^cm^–1^ in brackets): 329 (1.3 × 10^4^). IR (ATR, cm^–1^): 381, 451, 496, 531, 572, 631, 693, 743, 777, 836, 856, 884, 915, 999, 1026, 1093, 1110, 1138, 1199, 1227, 1253, 1285, 1360, 1386, 1423, 1459, 1477, 1587, 2865, 2905, 2950, 3054. Analysis calculated for C_78_H_90_O_3_P_3_Y: C 74.51. H 7.21. Found: C 75,19. H 7.52.

#### Lanthanum(III)tris[2,4-di-*tert*-butyl-6-(diphenylphosphan-yl)phenolate]silver(I) Triflate La^III^(OTf)(OAr^P^-1κ^1^*O*,2κ^1^*P*)_3_ Ag^I^**3-La**

A vial equipped with a magnetic stirrer bar was charged with Ag^I^OTf (103 mg, 400 μmol) and toluene (5 mL), and a solution of **2-La** (523 mg, 400 μmol) in toluene (5 mL) was added dropwise. The reaction mixture was stirred for 18 h at ambient temperature, after which the solution was filtered and the solvent was evaporated under reduced pressure. Then, the crude product was crystallized from THF/pentane, yielding colorless crystals of **3-La** (376 mg, 60%). mp 249 °C (dec.). ^1^H NMR (δ in ppm, benzene-*d*_6_, 298 K): 1.22 (s, 27H, ^*t*^Bu), 1.33 (s, 27H, ^*t*^Bu), 6.80 (br s, 6H, Ar*H*), 6.80 (ddd, ^3^*J*_(31)P,(1)H_ = 6 Hz, ^4^*J*_(107,109)Ag,(1)H_ = 3 Hz, ^4^*J*_(1)H,(1)H_ = 3 Hz, 3H, Ar*H*), 6.93 (br s, 3H, Ar*H*), 7.13 (br s, 3H, Ar*H*), 7.16 (s, 3H, Ar*H*), 7.41 (br s, 3H, Ar*H*), 7.48 (d, ^4^*J*_(1)H,(1)H_ = 3 Hz, 3H, Ar*H*). ^13^C{^1^H} NMR (δ in ppm, benzene-*d6*, 298 K): 29.8, 31.9, 34.5, 34.5, 118.5, 126.9, 129.1, 132.7, 135.1, 138.4, 139.2, 165.6. ^31^P{^1^H} NMR (δ in ppm, benzene-*d*_6_, 298 K): −0.5 (d, ^1^*J*_(31)P, (107)Ag_ = 325 Hz, d, ^1^*J*_(31)P, (109)Ag_ = 375 Hz). UV–vis (λ nm, ε Lmol^–1^cm^–1^ in brackets): 310 (1.5 × 10^4^). IR (ATR, cm^–1^): 409, 427, 446, 466, 499, 508, 525, 578, 589, 639, 692, 743, 772, 836, 883, 914, 1017, 1068, 1017, 1068, 1091, 1112, 1176, 1197, 1250, 1282, 1333, 1360, 1387, 1419, 1461, 1478, 2865, 2956, 3057, 3075.

#### Samarium(III)tris[2,4-di-*tert*-butyl-6-(diphenylphosphan-yl)phenolate]silver(I) Triflate Sm^III^(OTf)(OAr^P^-1κ^1^*O*,2κ^1^*P*)_3_ Ag^I^**3-Sm**

The complex was prepared via the same procedure that was employed for **3-La** by using **2-Sm** (660 mg, 500 μmol) and Ag^I^OTf (129 mg, 500 μmol). The complex was isolated as pale yellow crystals of **3-Sm** (532 mg, 68%). mp 296 °C (dec.). ^1^H NMR (δ in ppm, benzene-*d*_6_, 298 K): 1.33 (s, 27H, ^*t*^Bu), 2.43 (s, 27H, ^*t*^Bu), 6.57 (ddd, ^3^*J*_(31)P,(1)H_ = 6 Hz, ^4^*J*_(107,109)Ag,(1)H_ = 3 Hz, ^4^*J*_(1)H,(1)H_ = 3 Hz, 3H, Ar*H*), 6.70 (br s, 9H, Ar*H*), 7.06 (br s, 15H, Ar*H*), 7.16 (s, 3H, Ar*H*), 7.93 (d, ^4^*J*_(1)H,(1)H_ = 3 Hz, 3H, Ar*H*). ^13^C{^1^H} NMR (δ in ppm, benzene-*d*_6_, 298 K): 31.2, 31.8, 34.9, 36.2, 116.8, 127.1, 127.8, 129.8, 132.8, 134.3, 139.1, 139.3, 164.6. ^31^P{^1^H} NMR (δ in ppm, benzene-*d*_6_, 298 K): −1.0 (d, ^1^*J*_(31)P, (107)Ag_ = 325 Hz, d, ^1^*J*_(31)P, (109)Ag_ = 375 Hz). μ_eff_ = 1.2 μ_B_. UV–vis (λ nm, ε Lmol^–1^cm^–1^ in brackets): 308 (1.7 × 10^4^), 351 (688, sh), 382 (297, sh), 411 (58, sh). IR (ATR, cm^–1^): 426, 447, 466, 498, 508, 528, 578, 636, 691, 742, 770, 838, 860, 882, 915, 1011, 1069, 1092, 1113, 1180, 1197, 1232, 1251, 1281, 1325, 1339, 1360, 1388, 1421, 1459, 1476, 1588, 2865, 2900, 2956, 3055.

#### Ytterbium(III)tris[2,4-di-*tert*-butyl-6-(diphenylphosphan-yl)phenolate]silver(I) Triflate Yb^III^(OTf)(OAr^P^-1κ^1^*O*,2κ^1^*P*)_3_ Ag^I^**3-Yb**

The complex was prepared via the same procedure that was employed for **3-La** using **2-Yb** (537 mg, 400 μmol) and Ag^I^OTf (103 g, 400 μmol). The complex was isolated as yellow crystals of **3-Yb** (388 mg, 61%). mp 279 °C (dec.). ^1^H NMR (δ in ppm, benzene-*d*_6_, 298 K): −8.54 (s, 3H, Ar*H*), −3.13 (br s, 6H, Ar*H*), −2.43 (br s, 3H, Ar*H*), −2.24 (s, 27H, *^t^*Bu), 3.68 (vbr s, 6H, Ar*H*), 7.16 (s, 27H, *^t^*Bu), 10.74 (s, 3H, Ar*H*), 14.11 (br s, 3H, Ar*H*), 16.05 (br s, 6H, Ar*H*), 29.04 (br s, 6H, Ar*H*),. ^31^P{^1^H} NMR (δ ppm, benzene-*d*_6_): 54.0 (d, ^1^*J*_(31)P, (107, 109)Ag_ = 342 Hz). μ_eff_ = 4.7 μ_B_. UV–vis (λ nm, ε Lmol^–1^cm^–1^ in brackets): 305 (1.7 × 10^4^), 415 (2.6 × 10^2^). IR (ATR, cm^–1^): 409, 433, 447, 466, 498, 501, 534, 579, 634, 691, 741, 770, 844, 863, 882, 916, 1011, 1069, 1092, 1115, 1196, 1232, 1254, 1283, 1344, 1388, 1422, 1432, 1459, 1476, 1585, 2865, 2906, 2953, 3055.

#### Yttrium(III)tris[2,4-di-*tert*-butyl-6-(diphenylphosphanyl)-phenolate]silver(I) Triflate Y^III^(OTf)(OAr^P^-1κ^1^*O*,2κ^1^*P*)_3_Ag^I^**3-Y**

The complex was prepared via the same procedure that was employed for **3-La** using **2-Y** (503 mg, 400 μmol) and Ag^I^OTf (103 mg, 400 μmol). The complex was isolated as colorless crystals of **2-Y** (330 mg, 55%). mp 267 °C (dec.). ^1^H NMR (δ in ppm, benzene-*d*_6_, 298 K): 1.22 (s, 27H, ^*t*^Bu), 1.37 (s, 27H, ^*t*^Bu), 6.67–6.77 (m, 6H, Ar*H*), 6.80 (ddd, ^3^*J*_(31)P,(1)H_ = 6 Hz, ^4^*J*_(107,109)Ag,(1)H_ = 3 Hz, ^4^*J*_(1)H,(1)H_ = 3 Hz, 3H, Ar*H*), 6.82–6.92 (m, 6H, Ar*H*), 6.93–7.02 (m, 3H, Ar*H*), 7.06 (br s, 3H, Ar*H*), 7.16 (s, 3H, Ar*H*), 7.44 (d, ^4^*J*_(1)H,(1)H_ = 3 Hz, 3H, Ar*H*), 7.47 (br s, 3H, Ar*H*). ^13^C{^1^H} NMR (δ in ppm, benzene-*d*_6_, 298 K): 29.8, 31.9, 34.4, 34.5, 118.6, 126.7, 129.0, 132.7, 135.1, 139.1, 139.8, 163.3. ^31^P{^1^H} NMR (δ in ppm, benzene-*d*_6_, 298 K): −1.3 (d, ^2^*J*_(31)P, (107)Ag_ = 325 Hz, d, ^2^*J*_(31)P, (109)Ag_ = 375 Hz). UV–vis (λ nm, ε Lmol^–1^cm^–1^ in brackets): 306 (1.6 × 10^4^). IR (ATR, cm^–1^): 410, 434, 446, 466, 498, 508, 532, 579, 634, 691, 741, 772, 843, 861, 882, 916, 1011, 1067, 1092, 1115, 1186, 1196, 1234, 1254, 1283, 1344, 1359, 1388, 1422, 1459, 1478, 1587, 2869, 2949, 3060.

## Supporting Information Available

Crystallographic data, NMR, UV–vis, and IR spectroscopic data of the presented compounds and computational data and methods (PDF)
