# Supplementary material for: To Bond or Not to Bond: Metal–Metal Interaction in Heterobimetallic Rare-Earth Metal–Silver Complexes
Source: Inorg Chem. 2023 Oct 18;62(43):17713–20. doi: 10.1021/acs.inorgchem.3c02377 (PMC10618923; doi:10.1021/acs.inorgchem.3c02377)
Supplement: Supplementary file 1 — ic3c02377_si_001.pdf [file ic3c02377_si_001.pdf]

To Bond or Not to Bond: Metal-Metal Interaction in Heterobimetallic Rare-Earth Metal-Silver Complexes

Alexandra Haidinger,[b,†] Christina I. Dilly,[b,†] Roland C. Fischer,[b] Dennis Svatunek,[c] Johanna M. Uher,[b] and Johann A. Hlina[a,*]

[a]Institute of Chemistry, Inorganic Chemistry, University of Graz, Schubertstraße 1, 8010 Graz, Austria.

[b]Institute of Inorganic Chemistry, Graz University of Technology, Stremayrgasse 9, 8010 Graz, Austria.

[c]Institute of Applied Synthetic Chemistry, TU Wien, Getreidemarkt 9, 1060 Vienna, Austria.

[†]These authors contributed equally to this work.

Corresponding author e-mail: johann.hlina@uni-graz.at

Supporting Information

Table of Contents

Crystallography 2

- Additional Molecular Structures 2
- Crystallographic Data 4

NMR Spectroscopy 7

UV-vis Spectroscopy 19

IR Spectroscopy 24

Computational Studies 29

Crystallography:

Additional Molecular Structures:

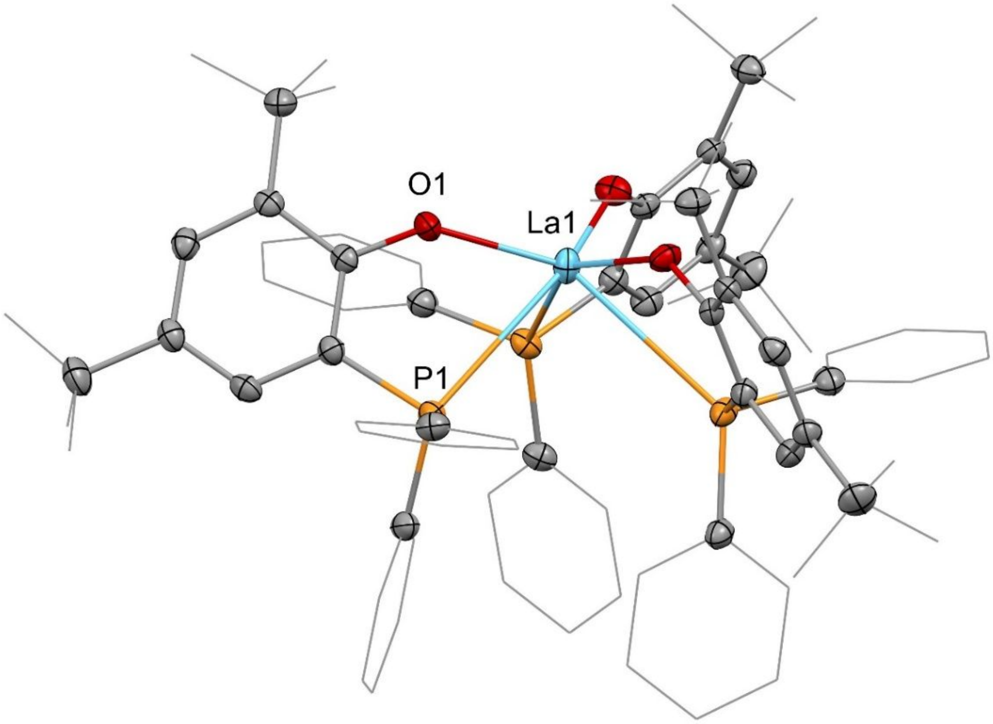

Figure S1. Molecular structure of **2-La**. Hydrogen atoms are omitted and selected carbon atoms depicted as wireframe for clarity. Thermal ellipsoids drawn at 50 % probability.

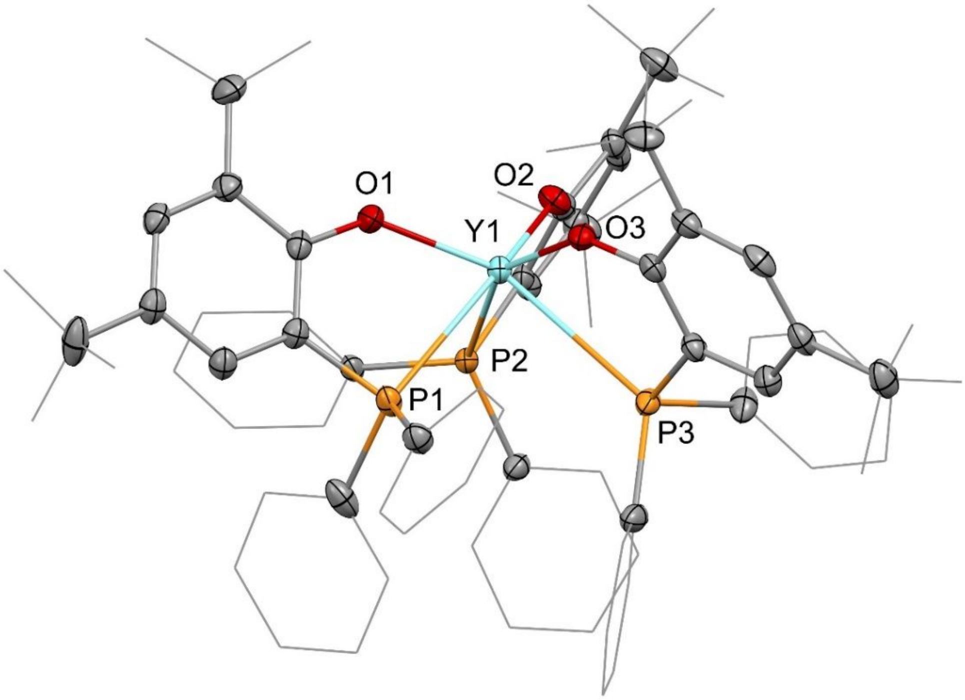

Figure S2. Molecular structure of **2-Y**. Hydrogen atoms are omitted and selected carbon atoms depicted as wireframe for clarity. Thermal ellipsoids drawn at 50 % probability.

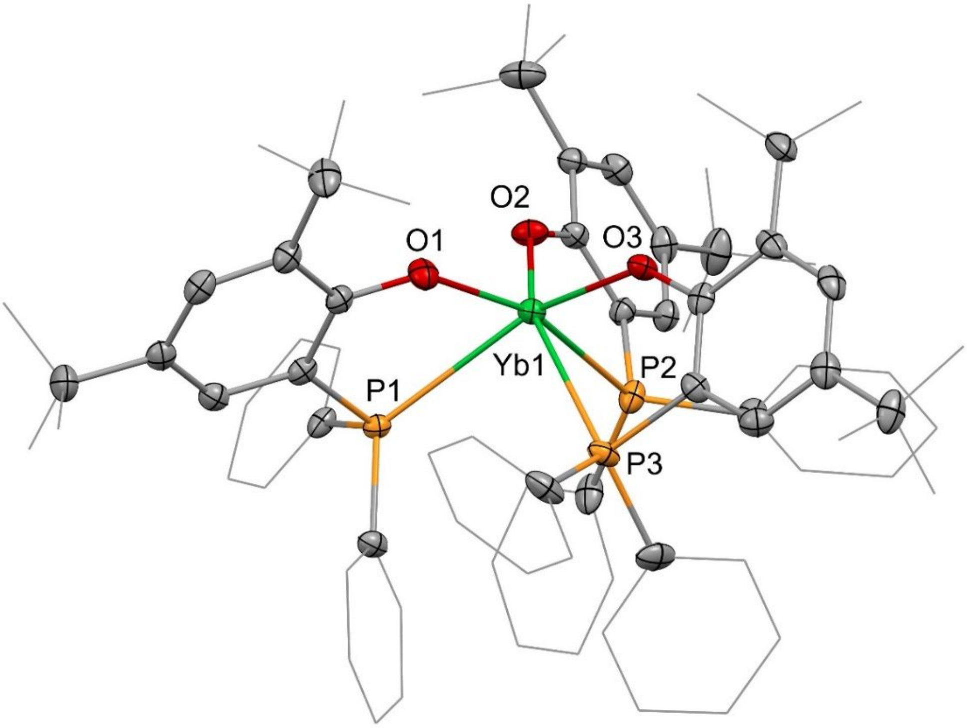

Figure S3. Molecular structure of **2-Yb**. Hydrogen atoms are omitted and selected carbon atoms depicted as wireframe for clarity. Thermal ellipsoids drawn at 50 % probability.

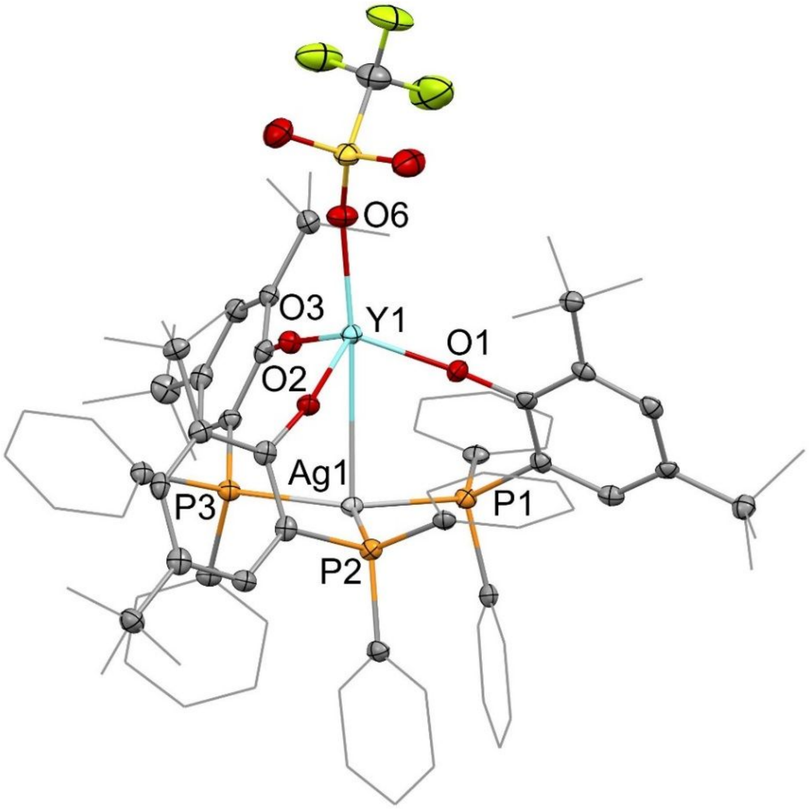

Figure S4. Molecular structure of **3-Y**. Hydrogen atoms are omitted and selected carbon atoms depicted as wireframe for clarity. Thermal ellipsoids drawn at 50 % probability.

Crystallographic Data:

Table S1. Crystallographic data of **2-La**, **2-Sm**, and **2-Yb**.

	**2-La**	**2-Sm**	**2-Yb**
CCDC number	2255810	2255811	2255808
Empirical formula	$C_{78}H_{90}LaO_3P_3$	$C_{78}H_{90}O_3P_3Sm$	$C_{84}H_{96}O_3P_3Yb$
Formula weight	1307.41	1307.41	1419.55
Temperature /K	150	150	99.8
Crystal system	trigonal	trigonal	monoclinic
Space group	*R*-3	*R*-3	$P2_1/c$
a /Å	22.605(3)	22.480(3)	22.0999(9)
b /Å	22.605(3)	22.480(3)	15.1402(6)
c /Å	24.599(6)	24.688(5)	23.0787(17)
α /°	90	90	90
β /°	90	90	100.929(2)
γ /°	120	120	90
Volume /Å^3	10886(3)	10805(4)	7582.0(7)
Z	6	6	4
ρ_{calc}g /cm^3	1.197	1.216	1.244
M /mm^{-1}	0.699	0.926	1.343
F(000)	4107.4	4134.0	2956.0
Crystal size /mm^3	0.2 x 0.18 x 0.18	0.33 x 0.3 x 0.3	0.25 x 0.18 x 0.1
2Θ range for data collection /°	2.66 to 52.72	2.66 to 52.8	3.566 to 53.998
Index ranges	-28<=h<=28, -28<=k<=28, -30<=l<=30	-28<=h<=28, -28<=k<=28, -30<=l<=30	-28<=h<=28, -19<=k<=19, -29<=l<=29
Reflections collected	28554	28900	189237
Independent reflections	4946 [R(int) = 0.0418]	4908 [R(int) = 0.0315]	16535 [R(int) = 0.1309]
Data/restraints/parameters	4946 / 79 / 293	4908 / 93 / 293	16535 / 585 / 1033
Goodness-of-fit on F^2	0.970	1.152	1.045
Final R indexes [I>=2σ (I)]	R1 = 0.0716, wR2 = 0.1415	R1 = 0.0352, wR2 = 0.0855	R1 = 0.0510, wR2 = 0.0883
Final R indexes [all data]	R1 = 0.0731, wR2 = 0.1424	R1 = 0.0361, wR2 = 0.0860	R1 = 0.0823, wR2 = 0.1001
Largest diff. peak/hole / e Å^{-3}	1.42 and -3.25	1.78 and -1.02	1.31 and -1.53

Table S2. Crystallographic data of **2-Y**, **2-Y·H$_2$O**, and **3-La**.

	**2-Y**	**2-Y·H$_2$O**	**3-La**
CCDC number	2255812	2255816	2255814
Empirical formula	$C_{85}H_{98}O_3P_3Y$	$C_{85}H_{100}O_4P_3Y$	$C_{79}H_{90}AgF_3LaO_6P_3S$
Formula weight	1349.45	1367.46	1564.25

Temperature /K	150	150	150
Crystal system	triclinic	monoclinic	monoclinic
Space group	*P*-1	$P2_1/c$	$P2_1/c$
a /Å	12.254(2)	15.528(2)	15.022(3)
b /Å	16.081(3)	16.841(3)	16.807(3)
c /Å	23.165(5)	32.433(5)	32.279(6)
α /°	91.870(4)	90	90
β /°	95.489(4)	98.351(3)	96.653(3)
γ /°	104.795(3)	90	90
Volume /Å^3	4385.5(14)	8392(2)	8095(3)
Z	2	4	4
ρ_{calc}g /cm^3	1.022	1.082	1.283
M /mm^{-1}	0.760	0.796	0.901
F(000)	1432.0	2904.0	3216.0
Crystal size /mm^3	0.44 x 0.18 x 0.1	0.14 x 0.1 x 0.08	0.24 x 0.18 x 0.15
2Θ range for data collection /°	1.77 to 52.742	3.394 to 46.996	2.736 to 52.662
Index ranges	-15<=h<=15,<br>-20<=k<=20,<br>-28<=l<=28	-17<=h<=17,<br>-18<=k<=18,<br>-36<=l<=36	-18<=h<=18,<br>-20<=k<=20,<br>-40<=l<=40
Reflections collected	35249	52642	63138
Independent reflections	17668 [R(int) = 0.0527]	12417 [R(int) = 0.1403]	16396 [R(int) = 0.0266]
Data/restraints/parameters	17668 / 263 / 871	12417 / 120 / 871	16396 / 129 / 890
Goodness-of-fit on F^2	1.004	1.198	1.056
Final R indexes [I>=2σ (I)]	R1 = 0.0744, wR2 = 0.1945	R1 = 0.1227, wR2 = 0.2356	R1 = 0.0291, wR2 = 0.0708
Final R indexes [all data]	R1 = 0.1083, wR2 = 0.2123	R1 = 0.1608, wR2 = 0.2542	R1 = 0.0323, wR2 = 0.0723
Largest diff. peak/hole / e Å^{-3}	1.28 and -1.03	0.90 and -1.00	0.86 and -0.43

Table S3. Crystallographic data of **3-Sm**, **3-Yb** and **3-Y**.

	**3-Sm**	**3-Yb**	**3-Y**
CCDC number	2255815	2255809	2255813
Empirical formula	$C_{91}H_{114}AgF_3O_6P_3SSm$	$C_{83}H_{98}AgF_3O_6P_3SYb$	$C_{79}H_{90}AgF_3O_6P_3SY$
Formula weight	1792.01	1670.49	1514.25
Temperature /K	150	99.7	100
Crystal system	monoclinic	monoclinic	monoclinic
Space group	$P2_1/n$	$P2_1/c$	$P2_1/c$
a /Å	13.741(3)	15.2764(8)	15.334(3)
b /Å	27.591(5)	16.6164(8)	16.630(3)
c /Å	23.983(5)	31.7533(16)	31.755(6)

α /°	90	90	90
β /°	90.020(3)	97.300(2)	97.177(3)
γ /°	90	90	90
Volume /Å^3	9093(3)	7994.9(7)	8034(3)
Z	4	4	4
ρ_{calc}g /cm^3	1.309	1.388	1.252
Μ /mm^{-1}	0.989	1.552	1.102
F(000)	3716.0	3428.0	3144.0
Crystal size /mm^3	0.38 x 0.33 x 0.24	0.29 x 0.16 x 0.07	0.28 x 0.12 x 0.09
2Θ range for data collection /°	3.312 to 52.746	3.564 to 56	2.77 to 52.76
Index ranges	-17<=h<=17, -34<=k<=34, -29<=l<=29	-20<=h<=20, -21<=k<=21, -41<=l<=41	-19<=h<=19, -20<=k<=20, -39<=l<=39
Reflections collected	71873	450213	52990
Independent reflections	18517 [R(int) = 0.0270]	19277 [R(int) = 0.0758]	16293 [R(int) = 0.0658]
Data/restraints/parameters	18517 / 149 / 1021	19277 / 0 / 916	16293 / 129 / 896
Goodness-of-fit on F^2	1.056	1.073	0.962
Final R indexes [I>=2σ (I)]	R1 = 0.0319, wR2 = 0.0815	R1 = 0.0279, wR2 = 0.0677	R1 = 0.0479, wR2 = 0.1022
Final R indexes [all data]	R1 = 0.0350, wR2 = 0.0832	R1 = 0.0345, wR2 = 0.0718	R1 = 0.0672, wR2 = 0.1086
Largest diff. peak/hole / e Å^{-3}	1.06 and -0.79	2.14 and -1.22	1.14 and -0.53

NMR Spectroscopy:

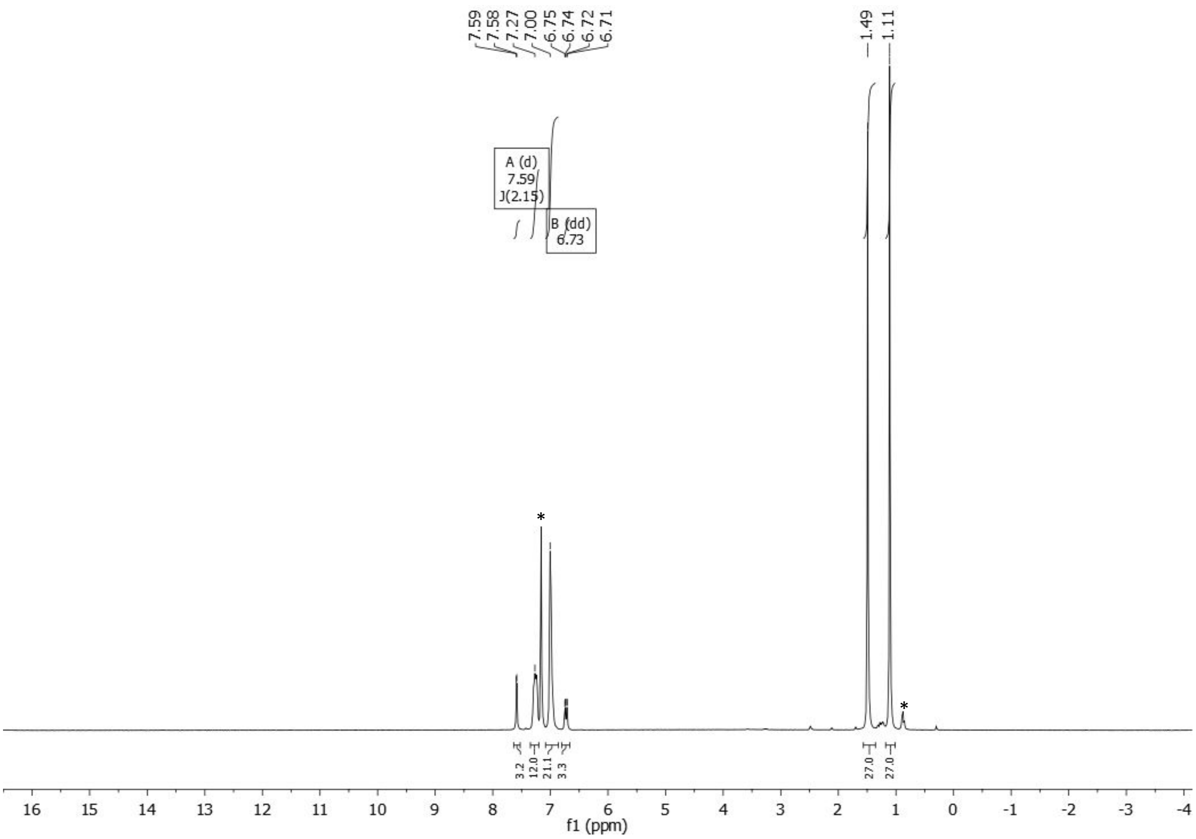

Figure S5. ¹H NMR spectrum of **1** in C_6D_6 recorded at 298 K. NMR solvent and impurities are marked with an asterisk.

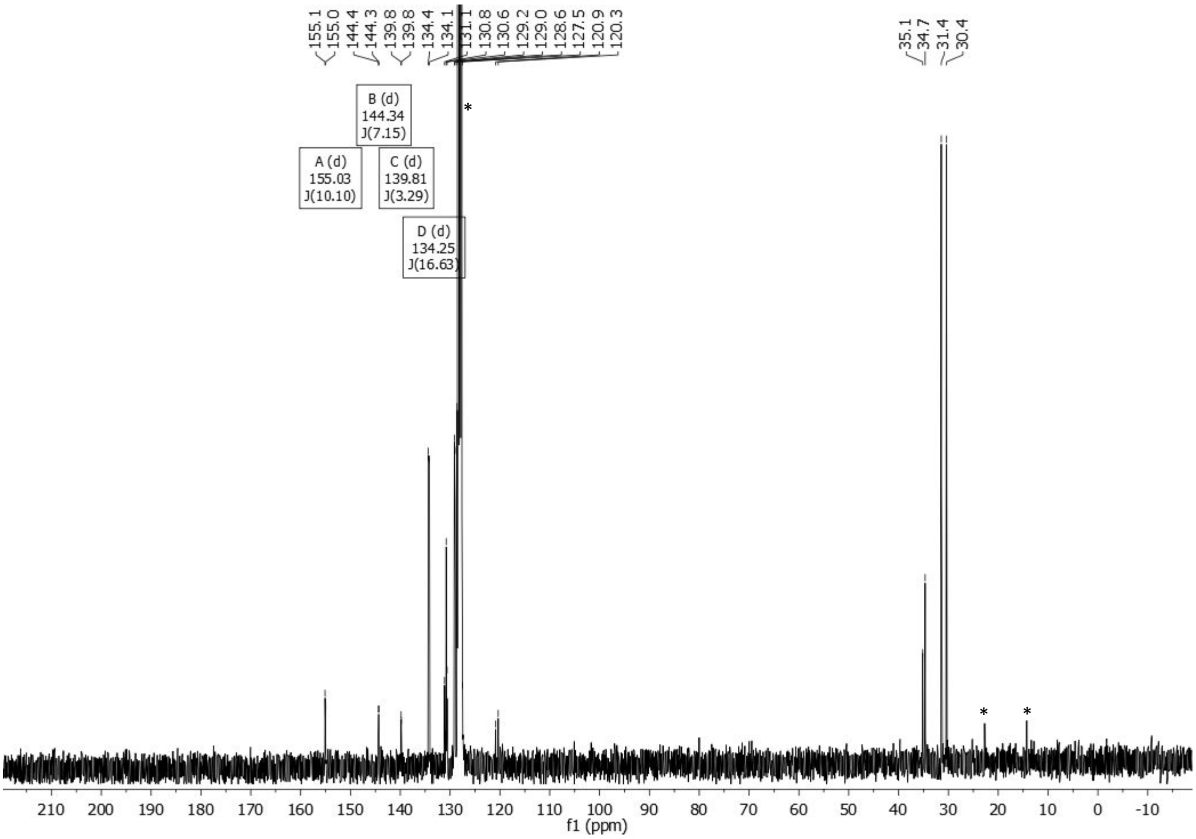

Figure S6. $^{13}C\{^1H\}$ NMR spectrum of **1** in C_6D_6 recorded at 298 K. NMR solvent and impurities are marked with an asterisk.

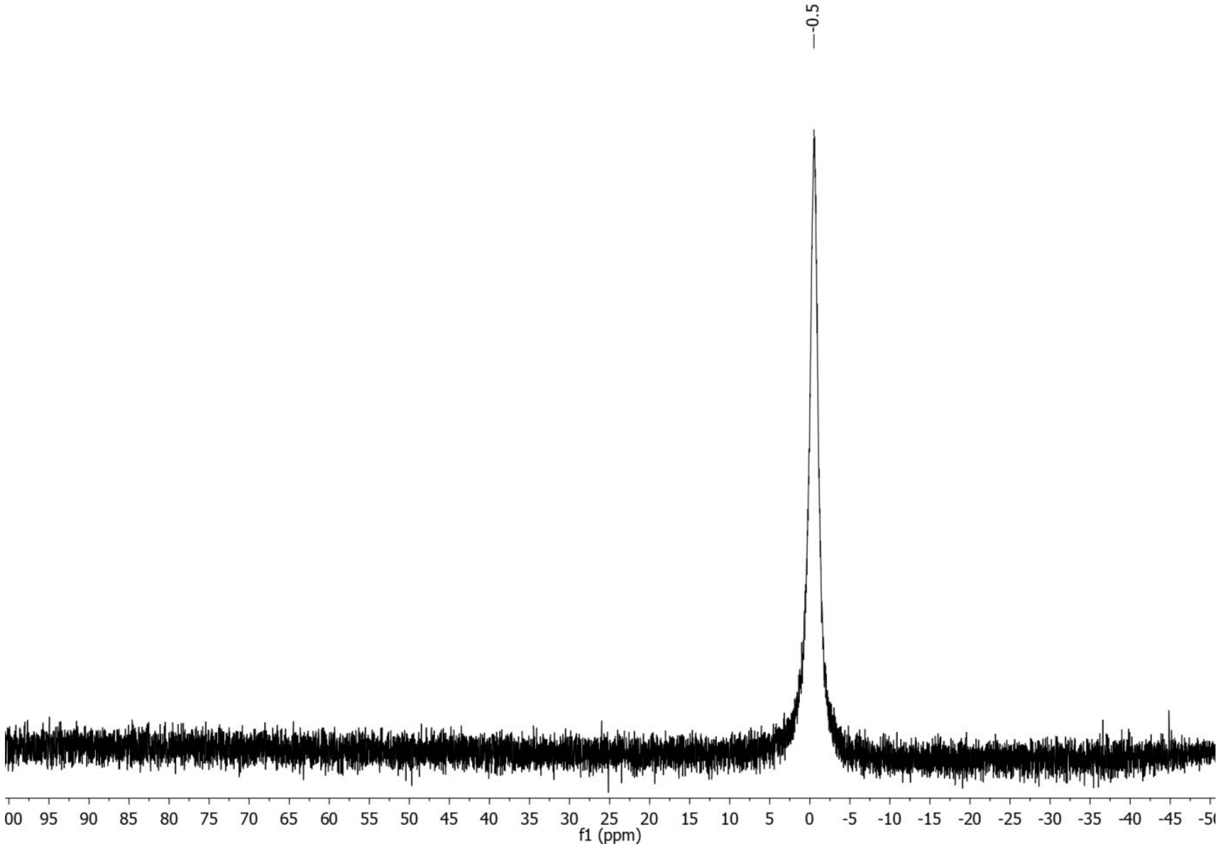

Figure S7. $^{31}P\{^{1}H\}$ NMR spectrum of **1** in C_6D_6 recorded at 298 K.

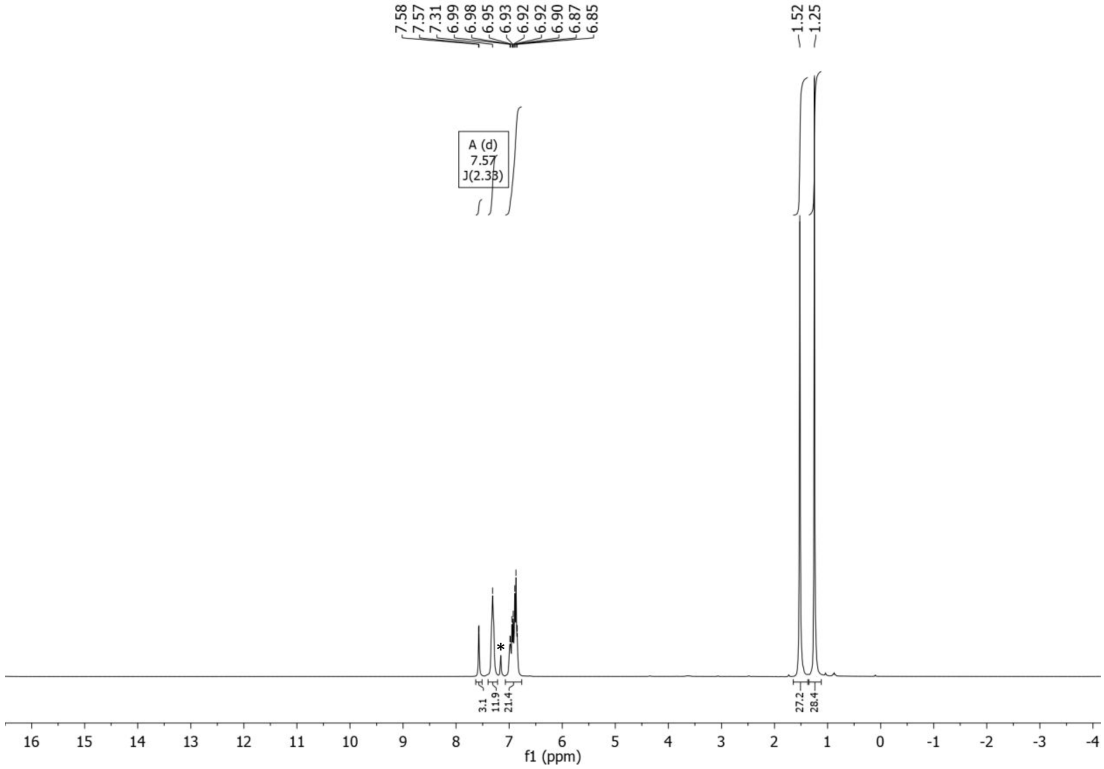

Figure S8. ^{1}H NMR spectrum of **2-La** in benzene-d6 recorded at 298 K. NMR solvent is marked with an asterisk.

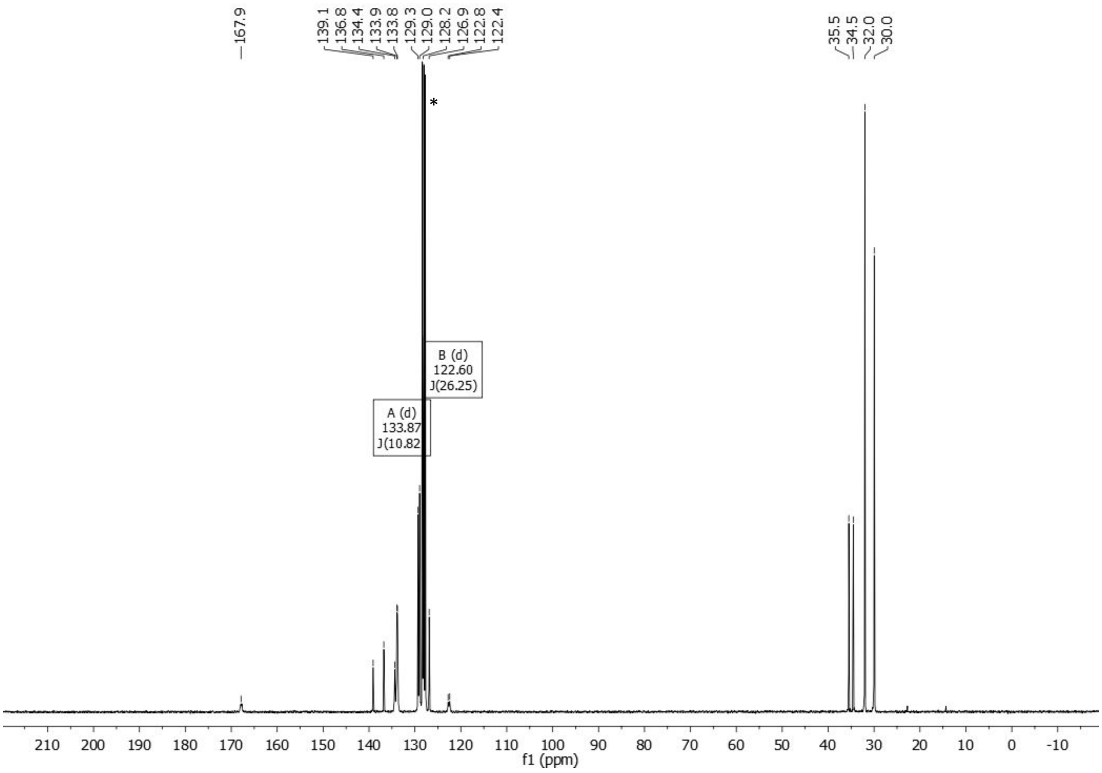

Figure S9. $^{13}C\{^{1}H\}$ NMR spectrum of **2-La** in benzene-d6 recorded at 298 K. NMR solvent is marked with an asterisk.

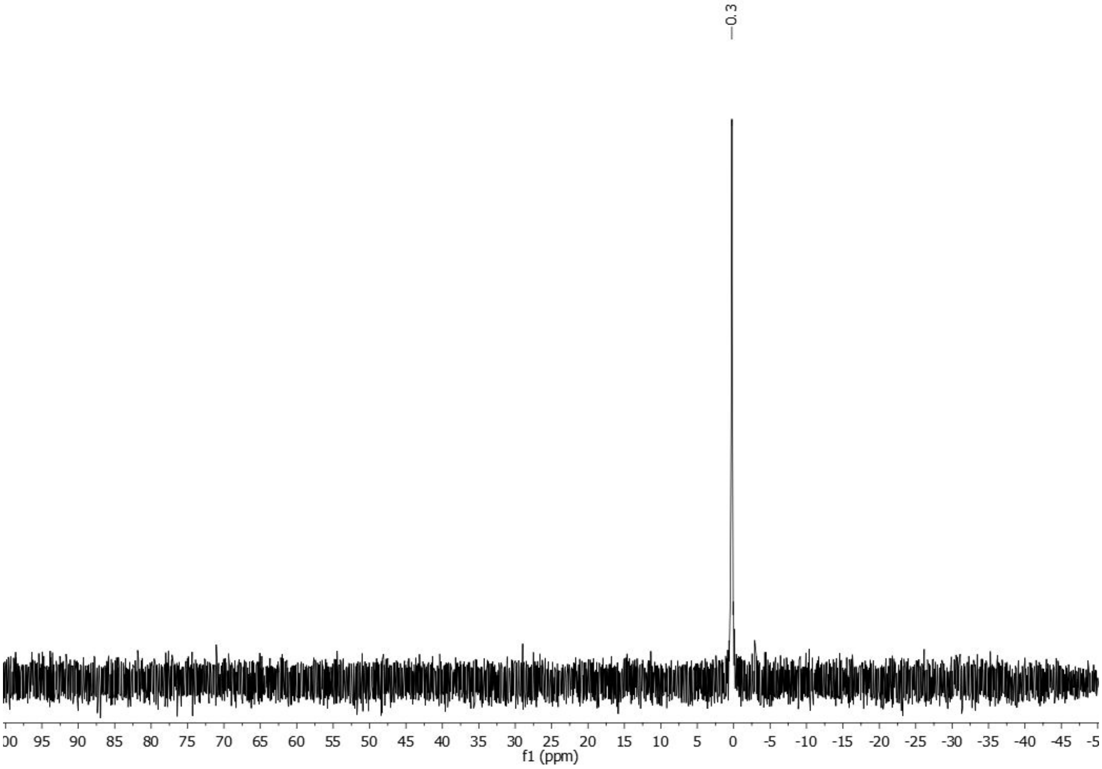

Figure S10. $^{31}P\{^{1}H\}$ NMR spectrum of **2-La** in benzene-d6 recorded at 298 K.

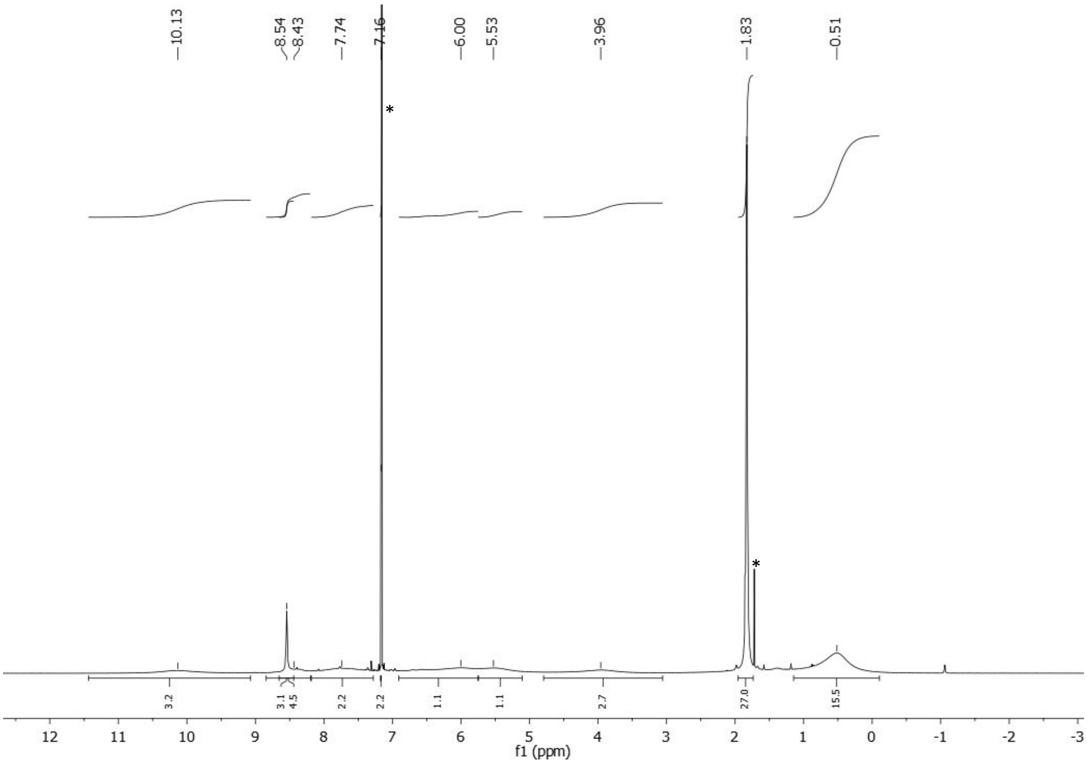

Figure S11. ^{1}H NMR spectrum of **2-Sm** in benzene-d6 recorded at 298 K. NMR solvent and impurities are marked with an asterisk.

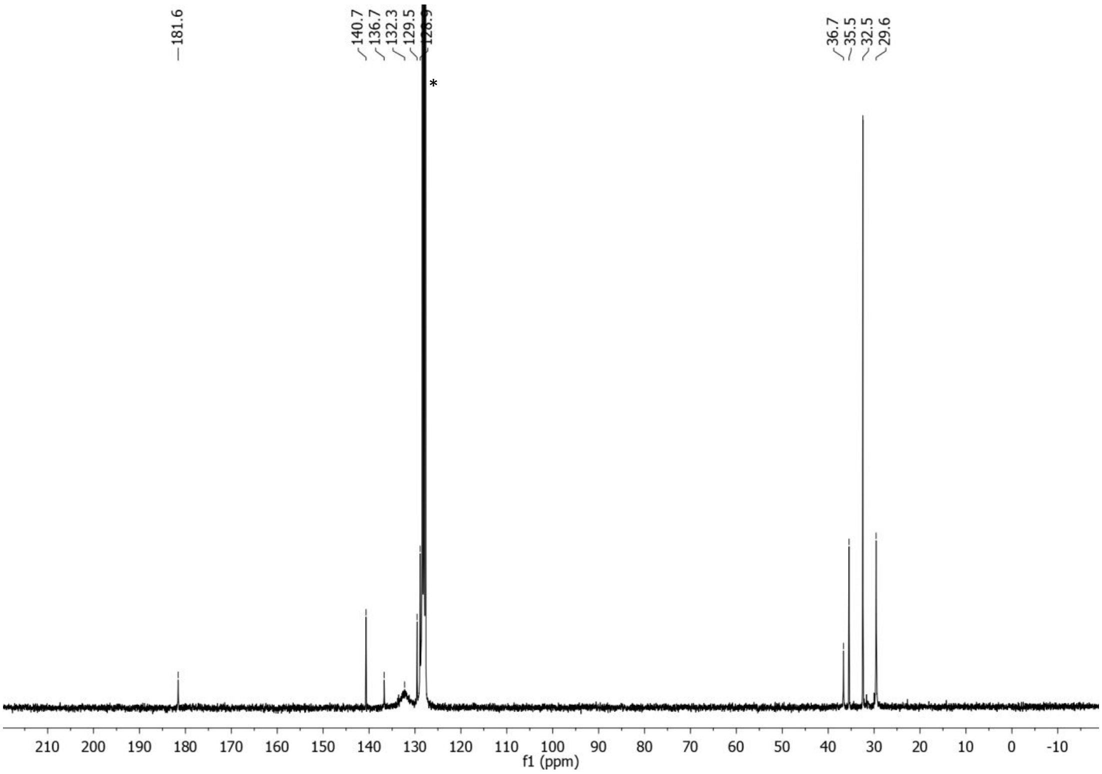

Figure S12. $^{13}C\{^{1}H\}$ NMR spectrum of **2-Sm** in benzene-d6 recorded at 298 K. NMR solvent is marked with an asterisk.

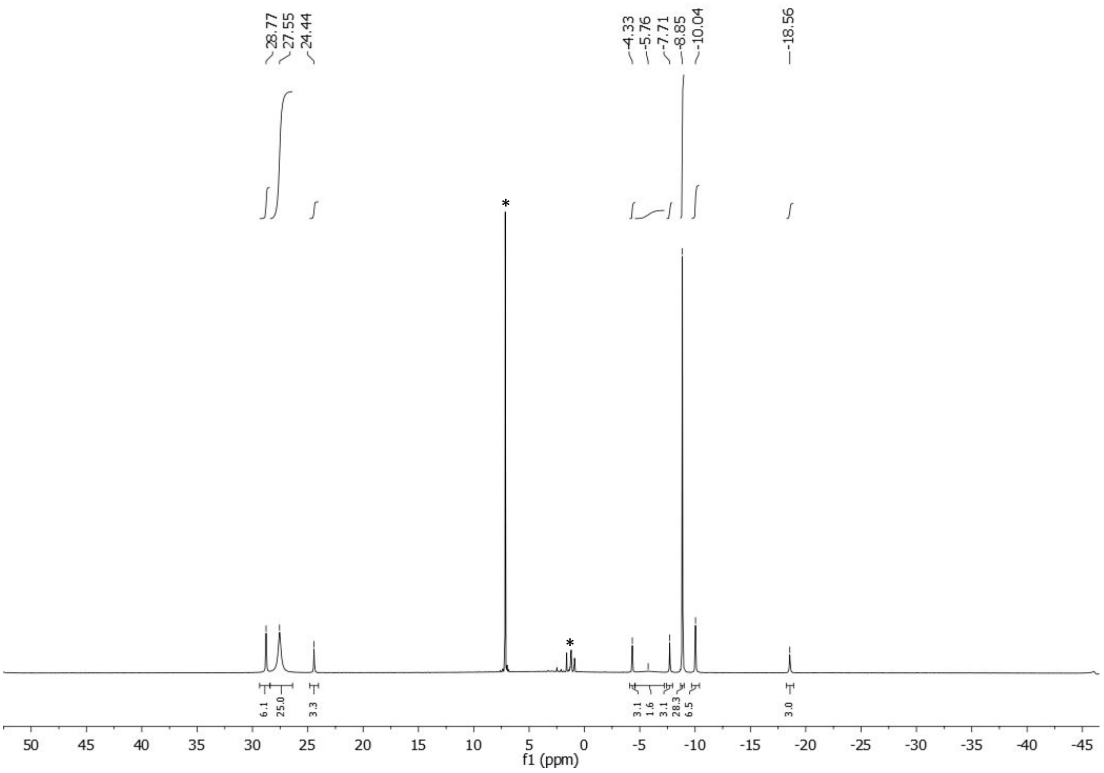

Figure S13. ^{1}H NMR spectrum of **2-Yb** in benzene-d6 recorded at 298 K. NMR solvent and impurities are marked with an asterisk.

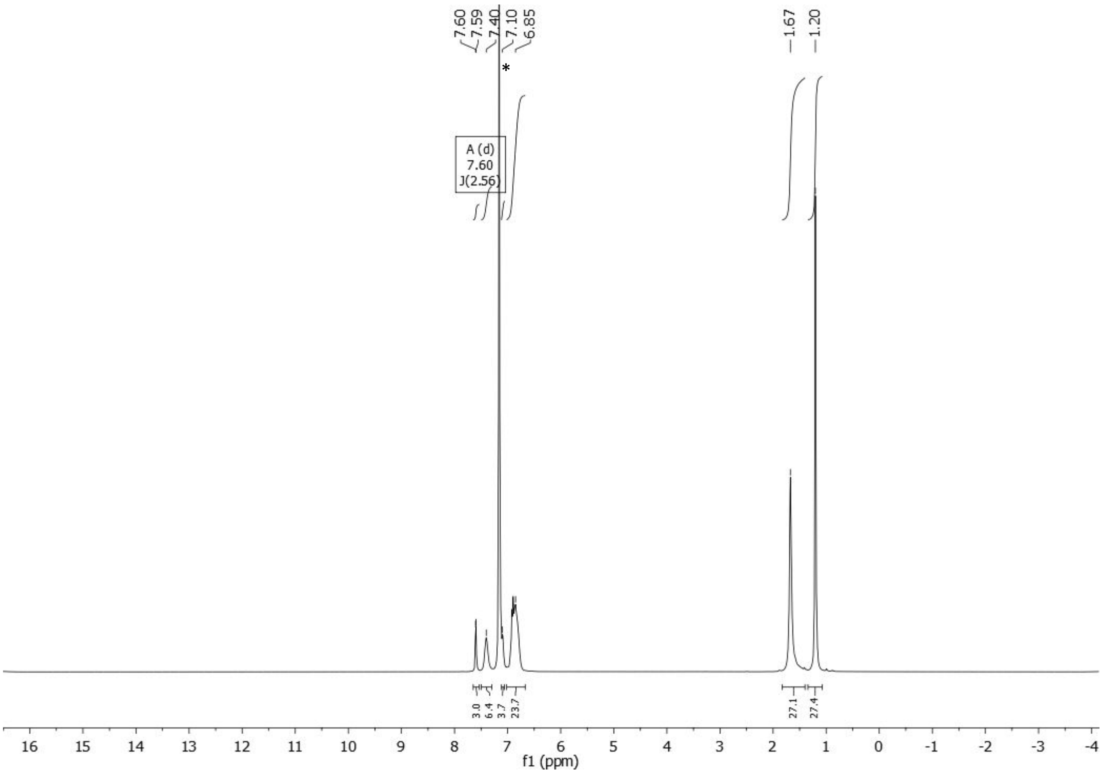

Figure S14. ^{1}H NMR spectrum of **2-Y** in benzene-d6 recorded at 298 K. NMR solvent is marked with an asterisk.

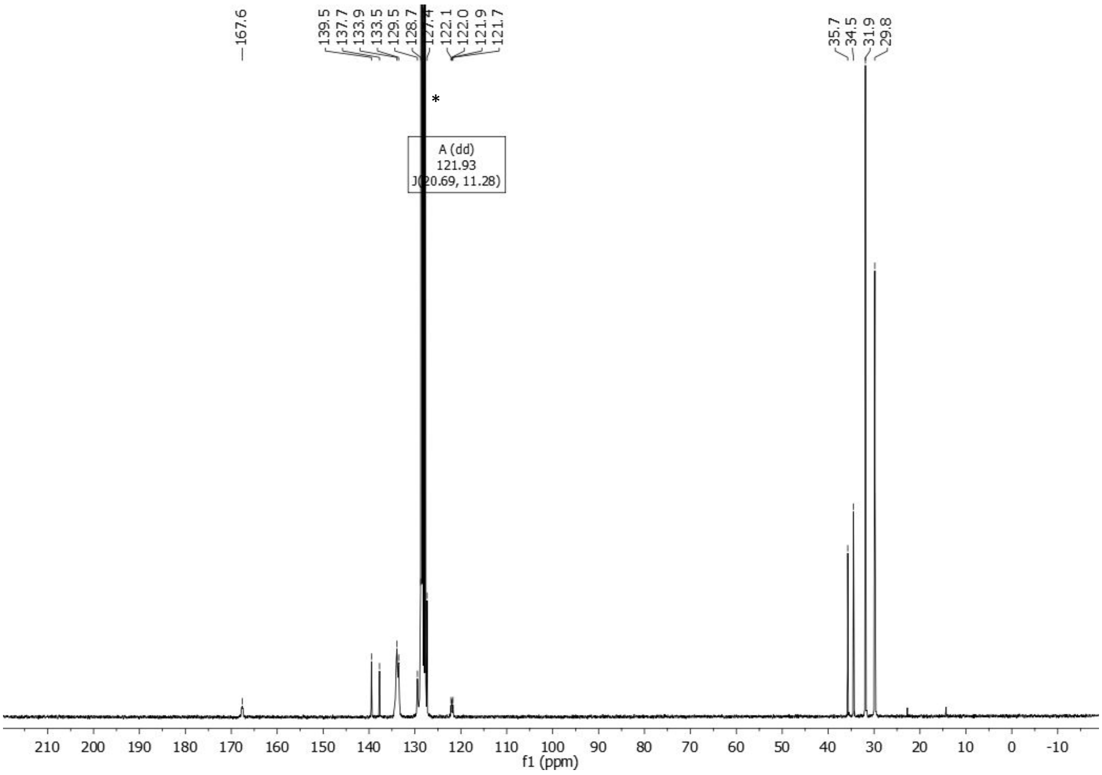

Figure S15. $^{13}C\{^1H\}$ NMR spectrum of **2-Y** in benzene-d6 recorded at 298 K. NMR solvent is marked with an asterisk.

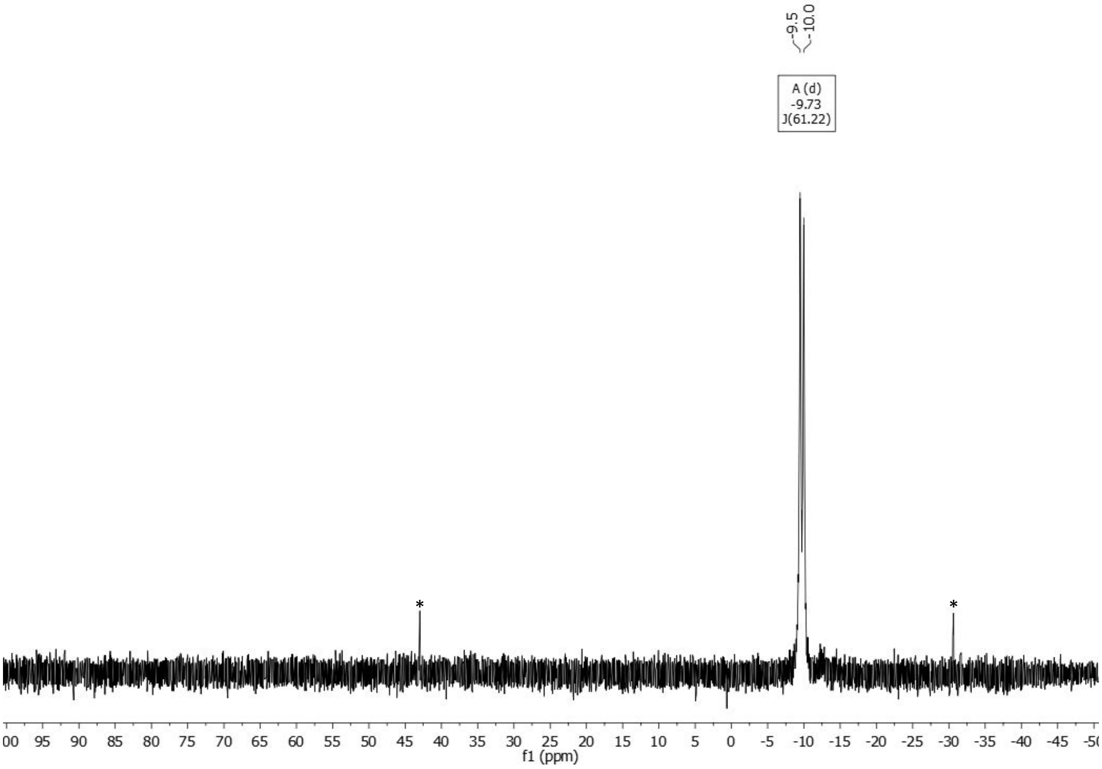

Figure S16. $^{31}P\{^1H\}$ NMR spectrum of **2-Y** in benzene-d6 recorded at 298 K. Impurities are marked with an asterisk.

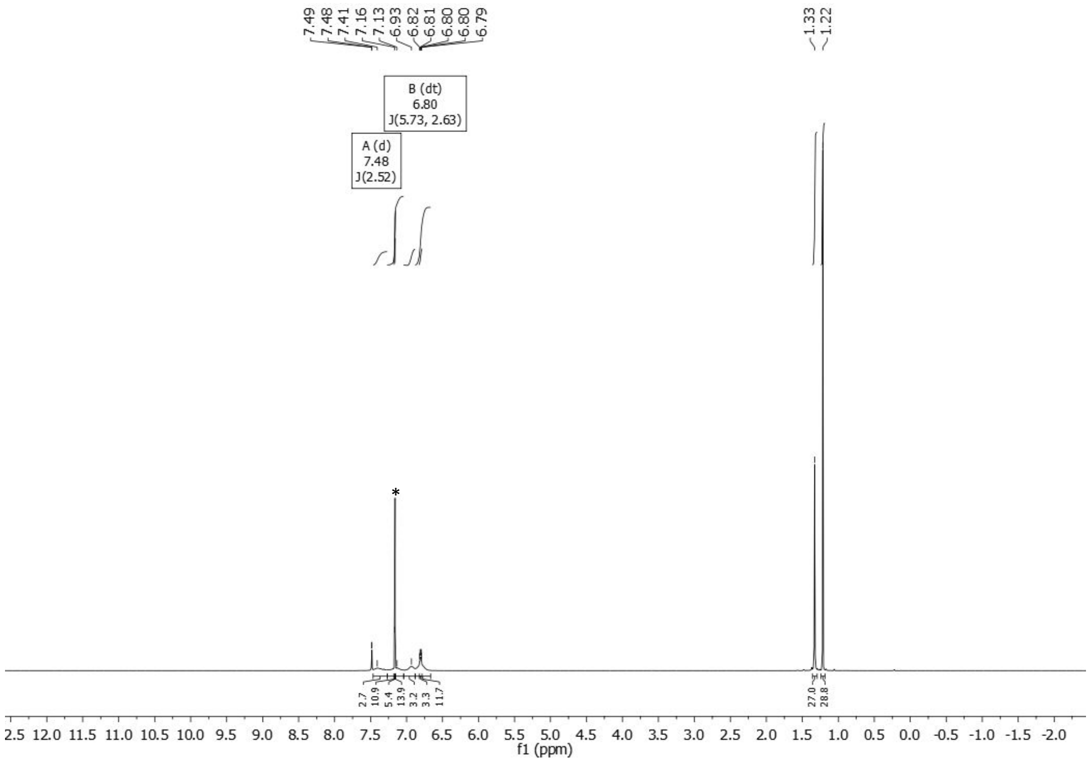

Figure S17. ^{1}H NMR spectrum of **3-La** in C_6D_6 recorded at 298 K. NMR solvent is marked with an asterisk.

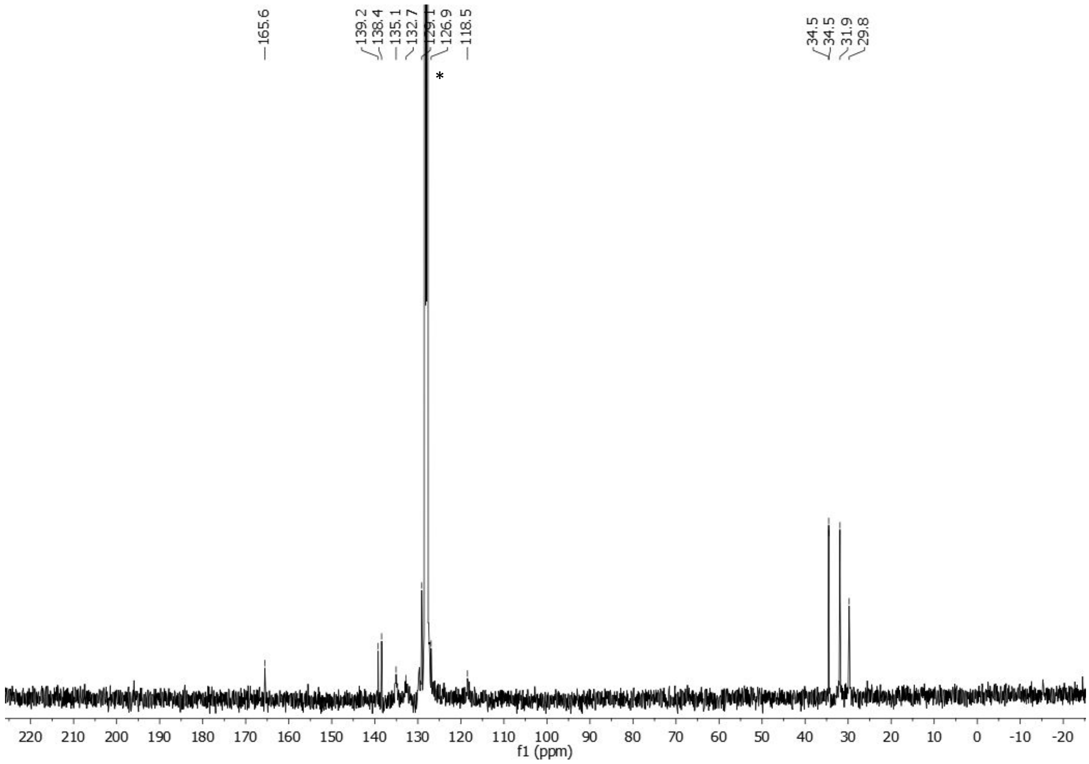

Figure S18. $^{13}C\{^{1}H\}$ NMR spectrum of **3-La** in C_6D_6 recorded at 298 K. NMR solvent is marked with an asterisk.

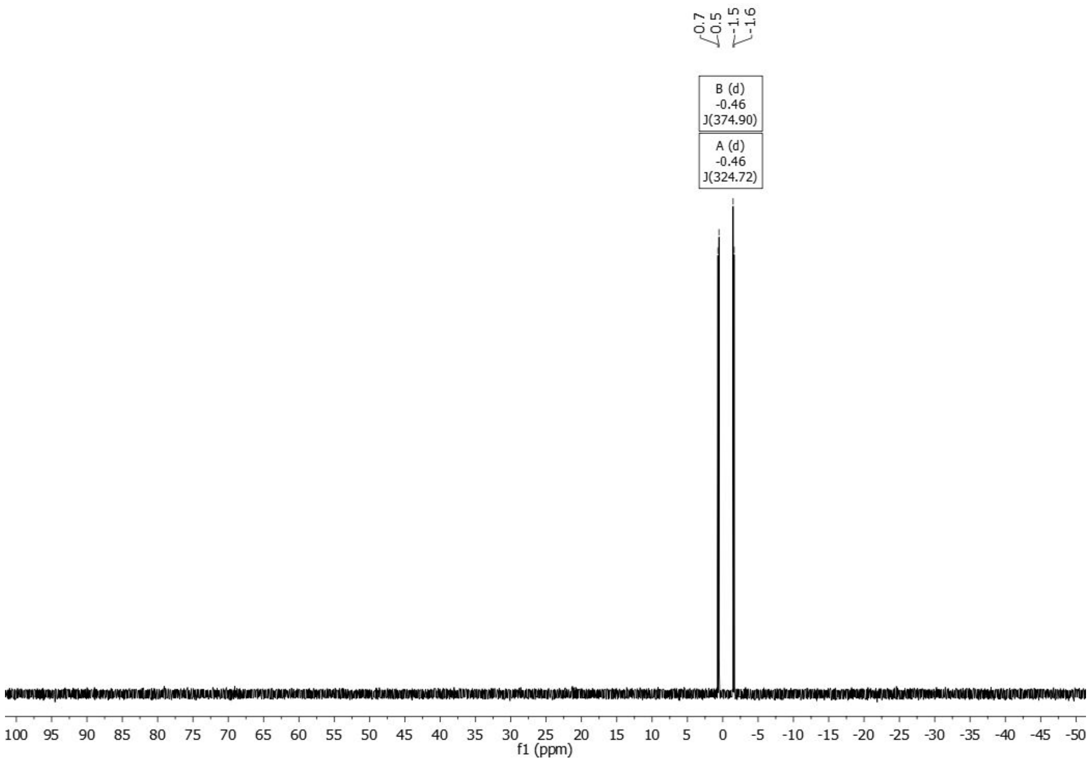

Figure S19. $^{31}P\{^{1}H\}$ NMR spectrum of **3-La** in C_6D_6 recorded at 298 K.

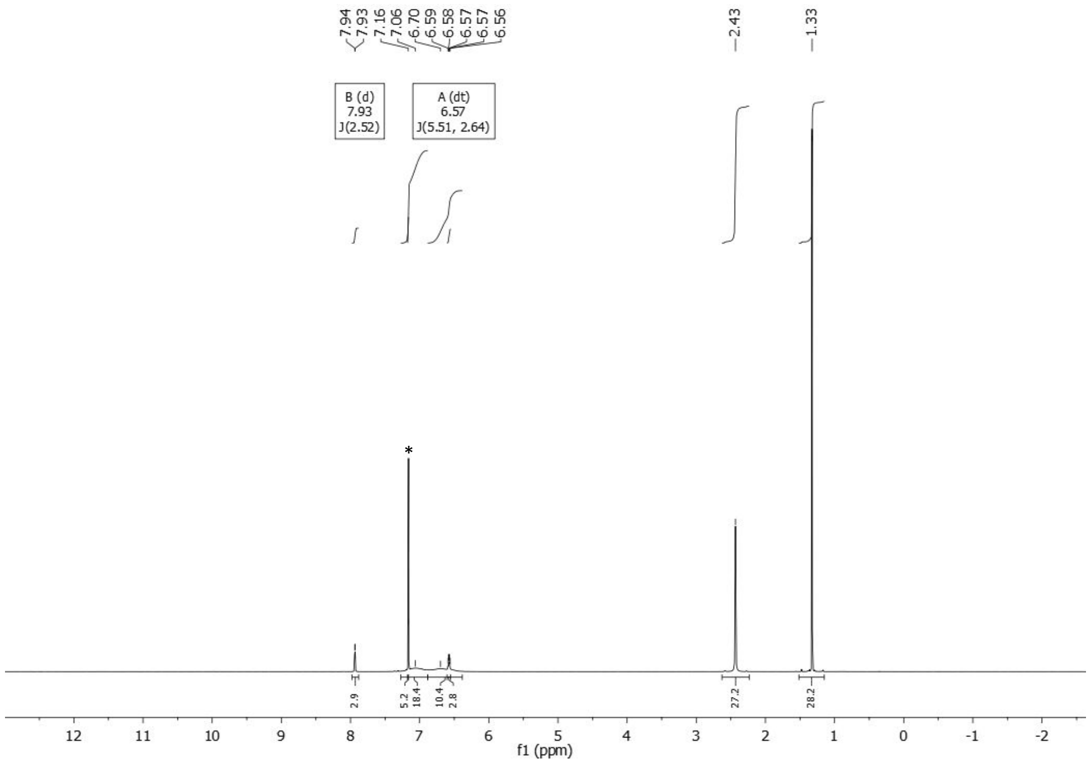

Figure S20. ^{1}H NMR spectrum of **3-Sm** in C_6D_6 recorded at 298 K. NMR solvent is marked with an asterisk.

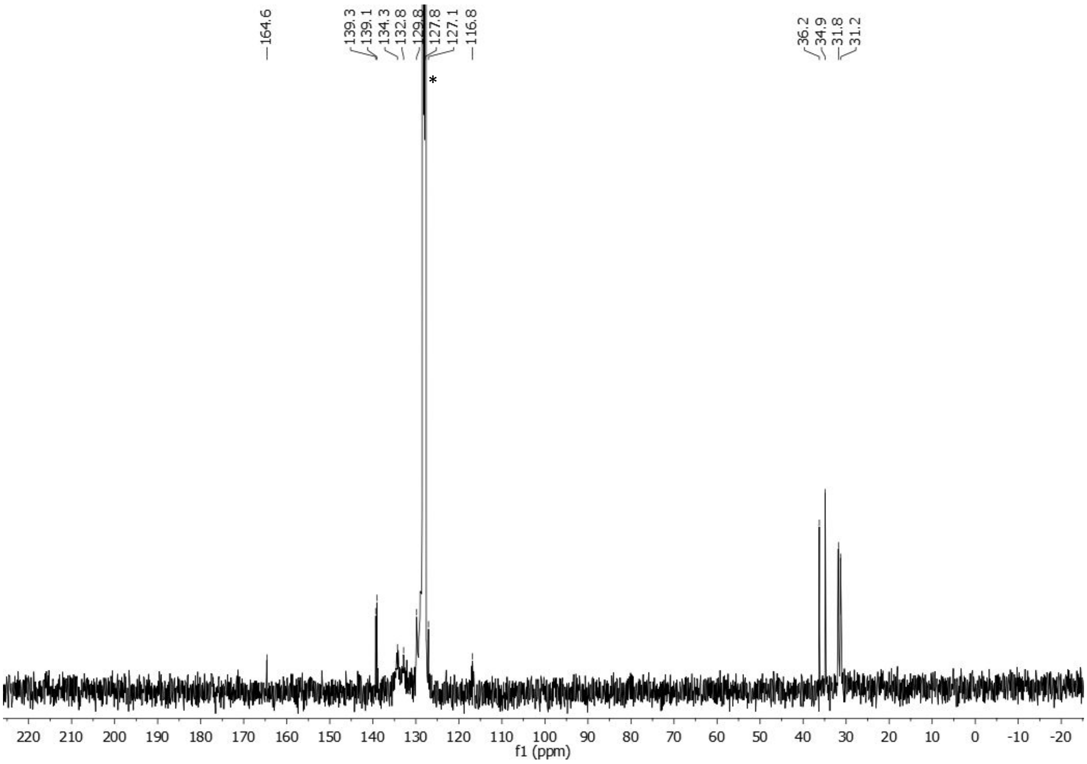

Figure S21. $^{13}C\{^1H\}$ NMR spectrum of **3-Sm** in C_6D_6 recorded at 298 K. NMR solvent is marked with an asterisk.

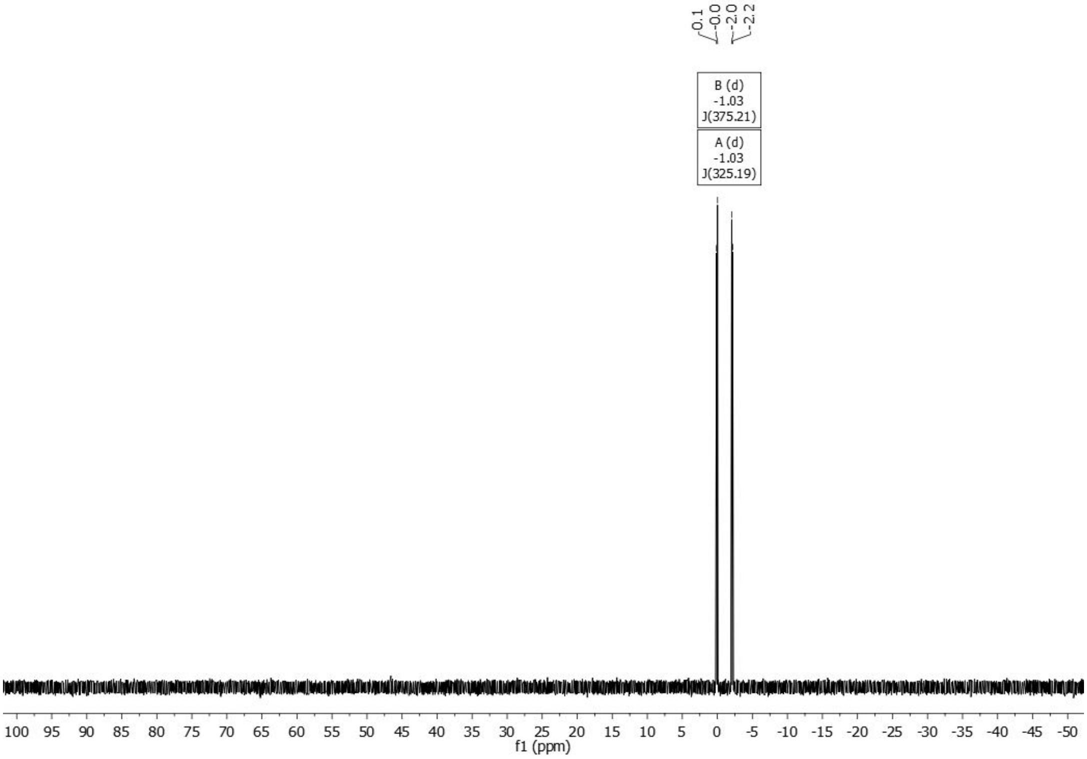

Figure S22. $^{31}P\{^1H\}$ NMR spectrum of **3-Sm** in C_6D_6 recorded at 298 K.

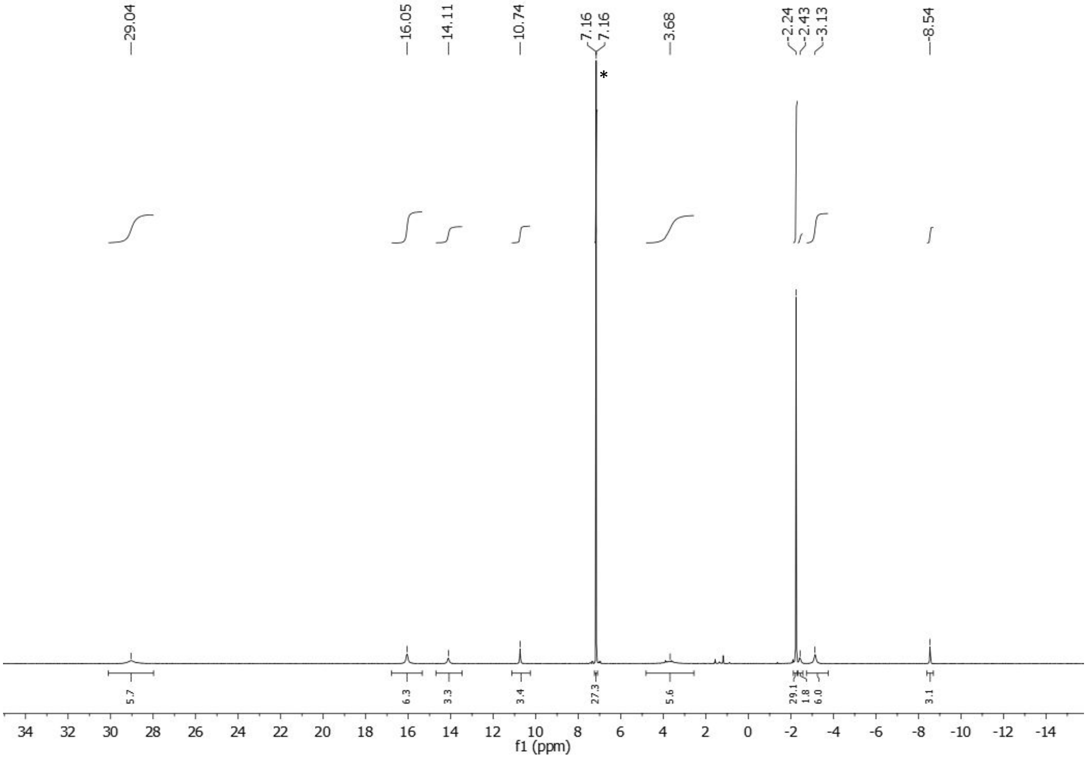

Figure S23. ^{1}H NMR spectrum of **3-Yb** in C_6D_6 recorded at 298 K. NMR solvent is marked with an asterisk.

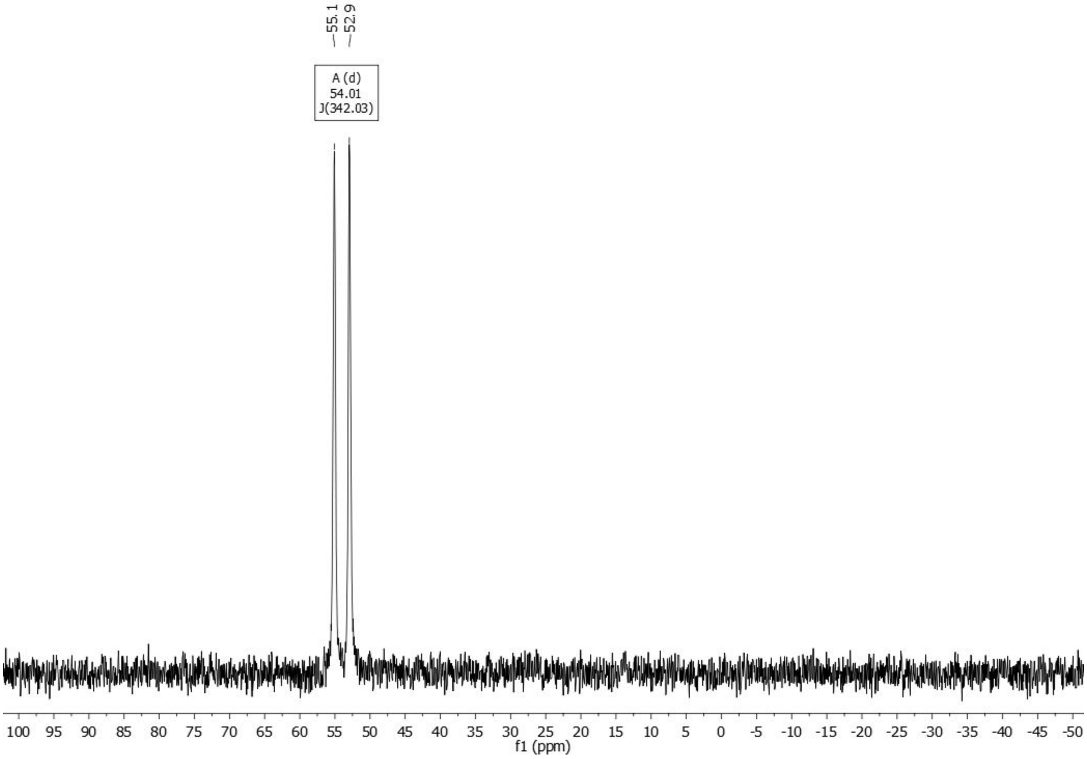

Figure S24. $^{31}P\{^{1}H\}$ NMR spectrum of **3-Yb** in C_6D_6 recorded at 298 K.

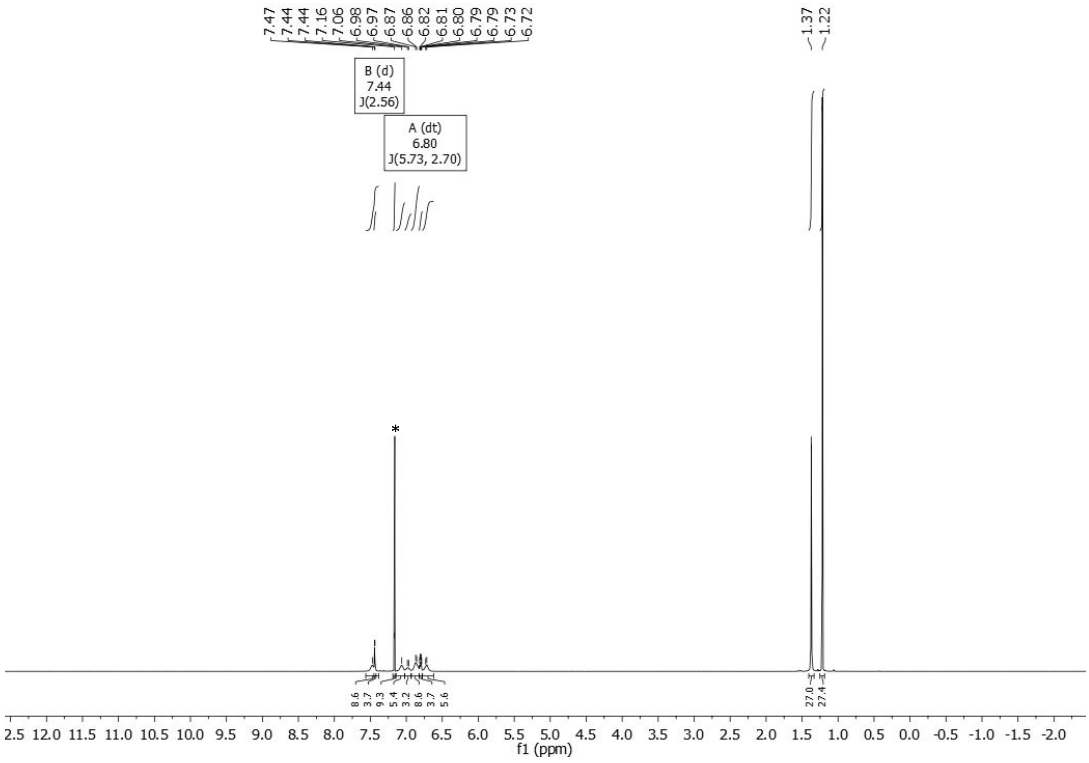

Figure S25. ^{1}H NMR spectrum of **3-Y** in C_6D_6 recorded at 298 K. NMR solvent is marked with an asterisk.

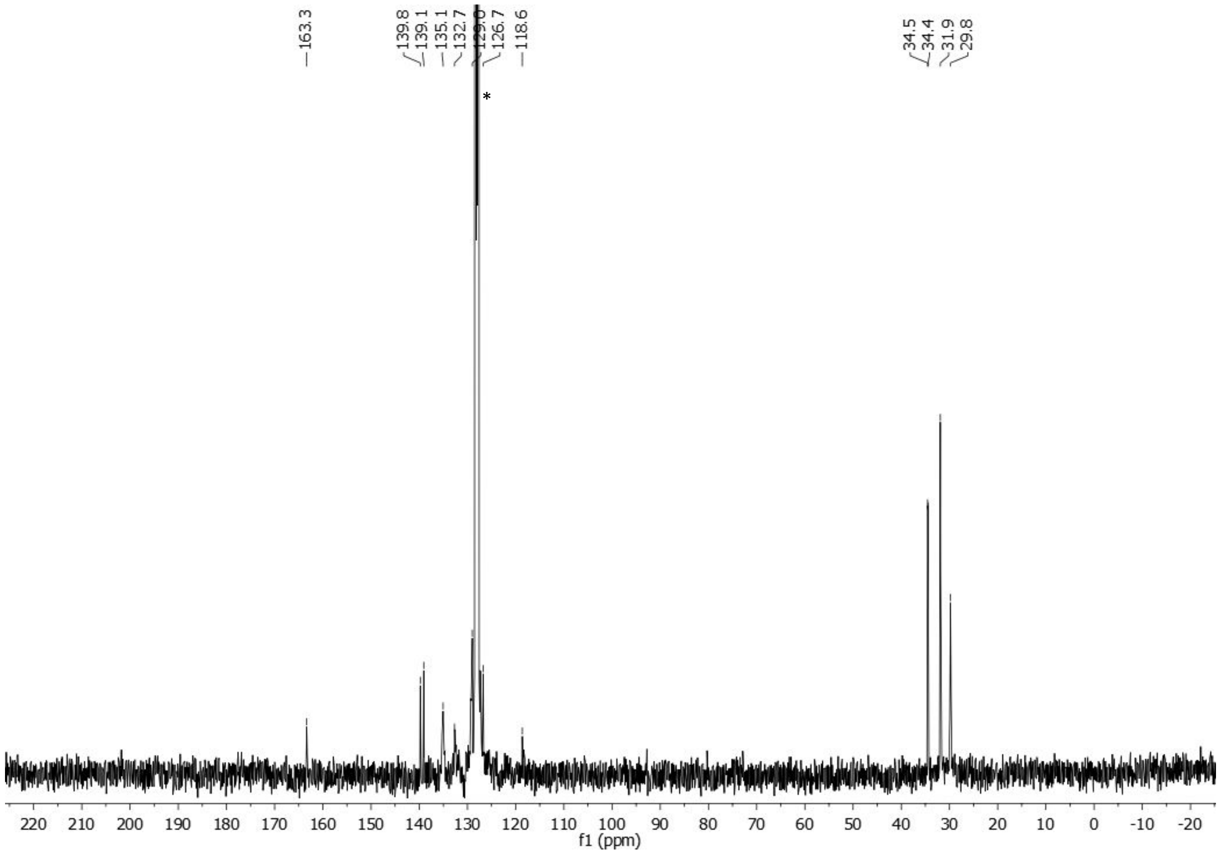

Figure S26. $^{13}C\{^{1}H\}$ NMR spectrum of **3-Y** in C_6D_6 recorded at 298 K. NMR solvent is marked with an asterisk.

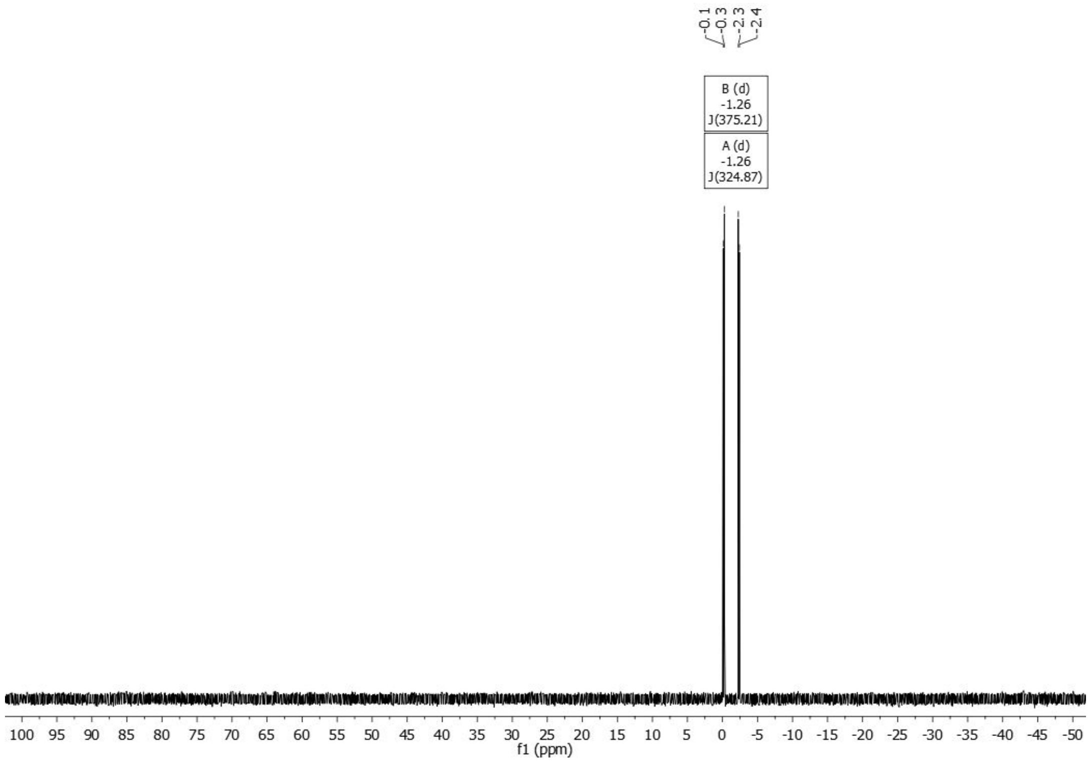

Figure S27. $^{31}P\{^1H\}$ NMR spectrum of **3-Y** in C_6D_6 recorded at 298 K.

UV-vis Spectroscopy:

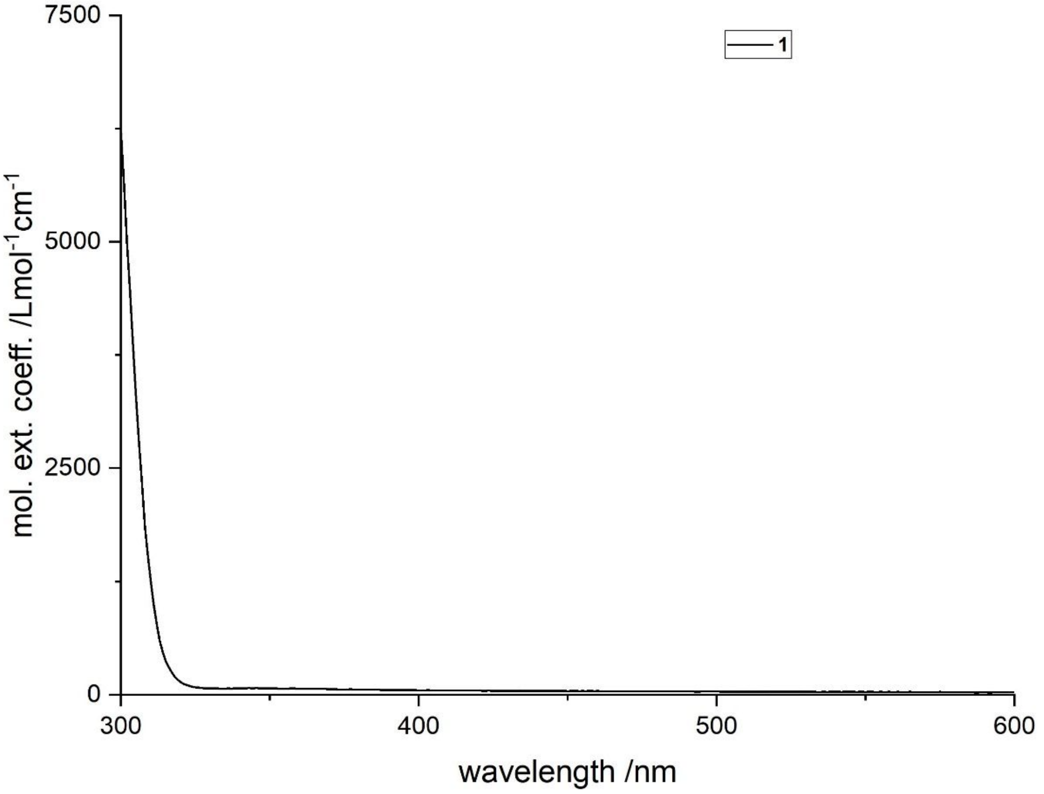

Figure S28. UV-vis spectrum of **1** in toluene.

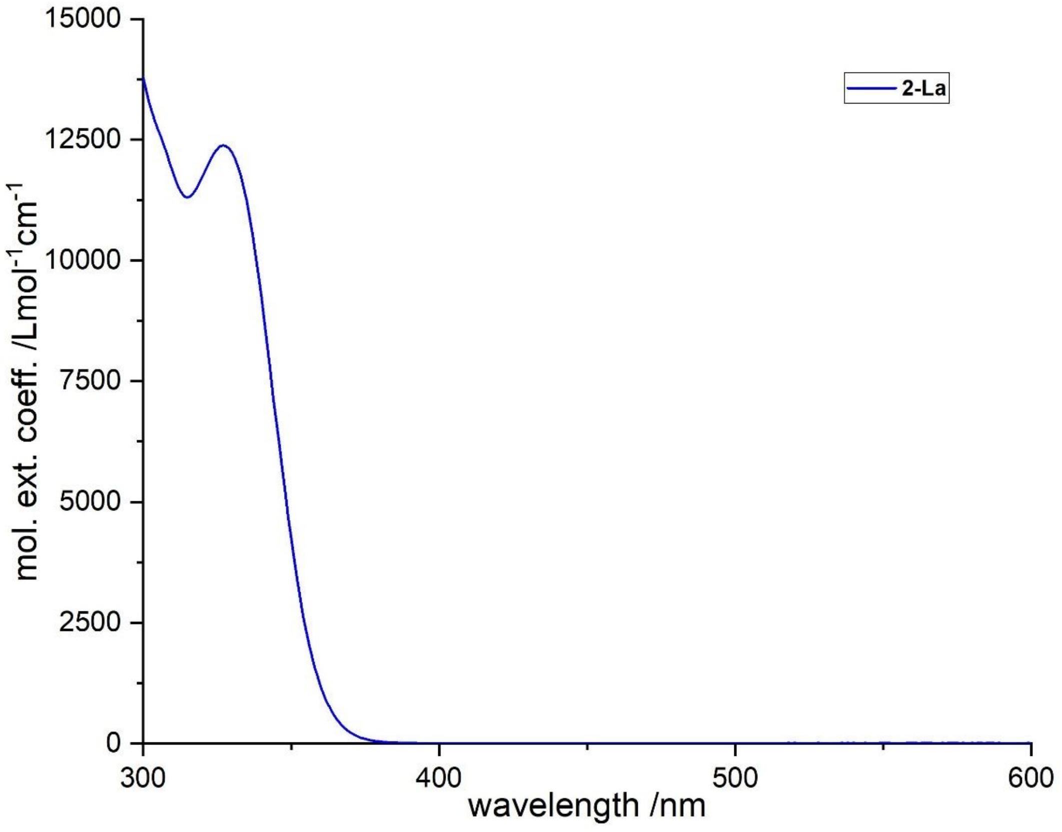

Figure S29. UV-vis spectrum of **2-La** in toluene.

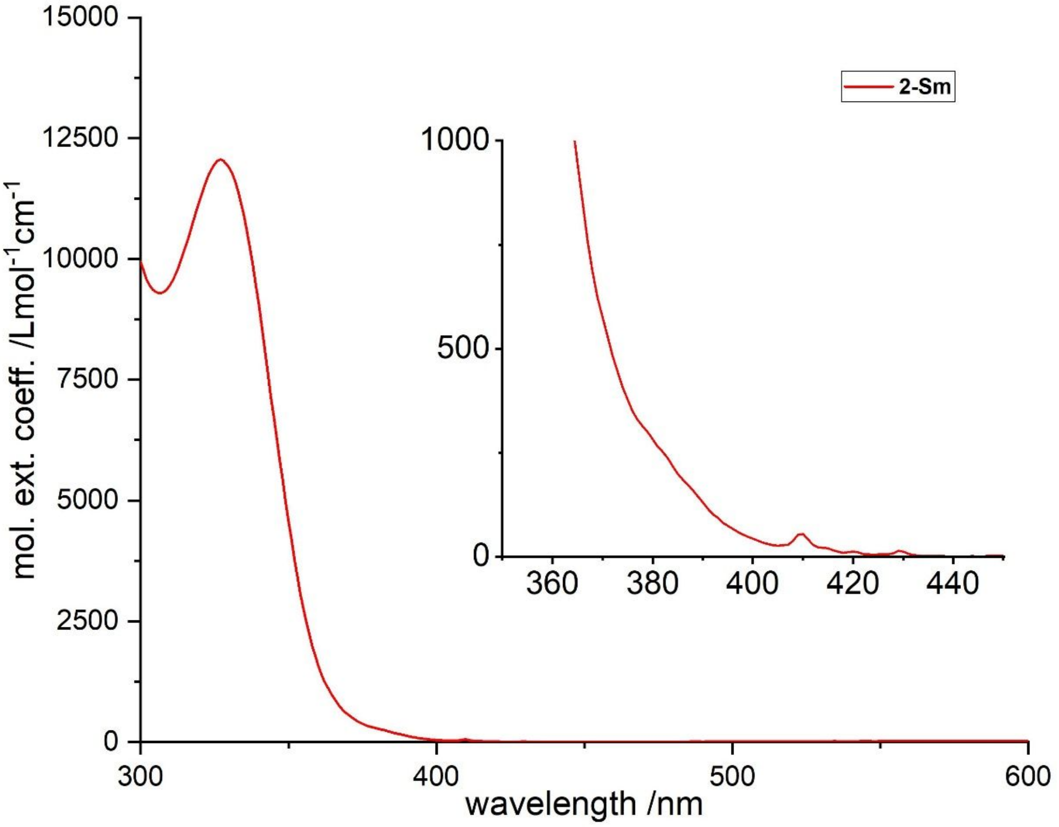

Figure S30. UV-vis spectrum of **2-Sm** in toluene.

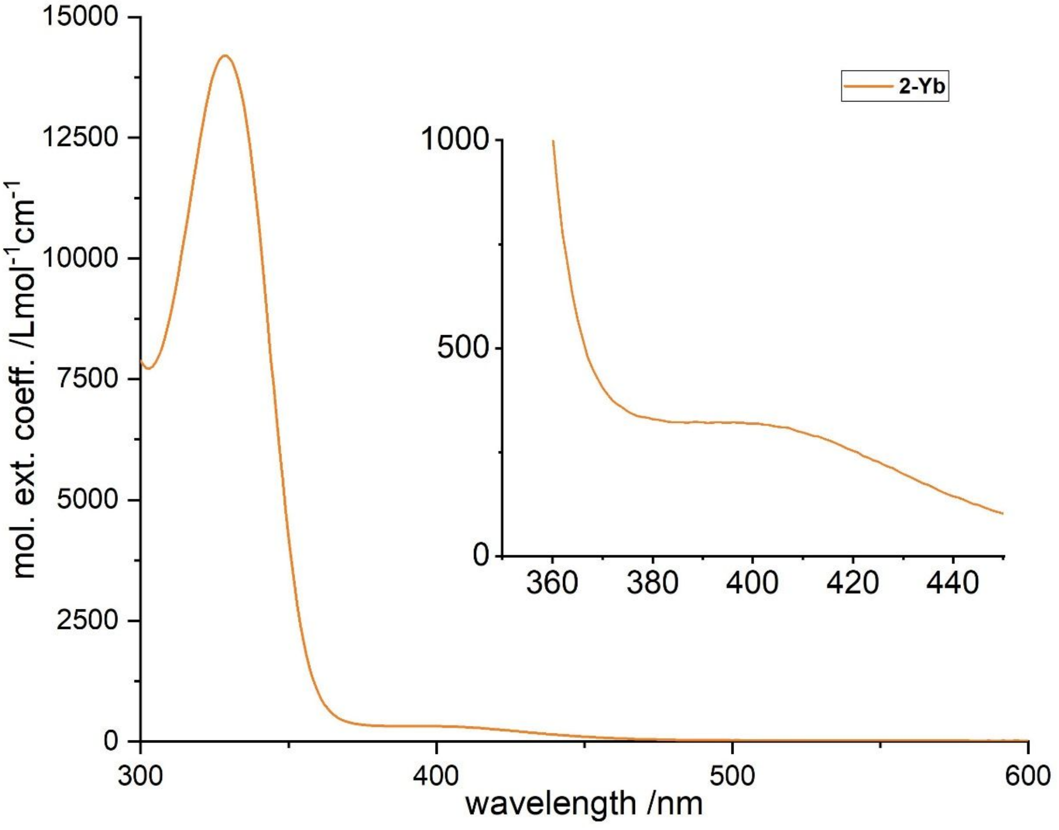

Figure S31. UV-vis spectrum of **2-Yb** in toluene.

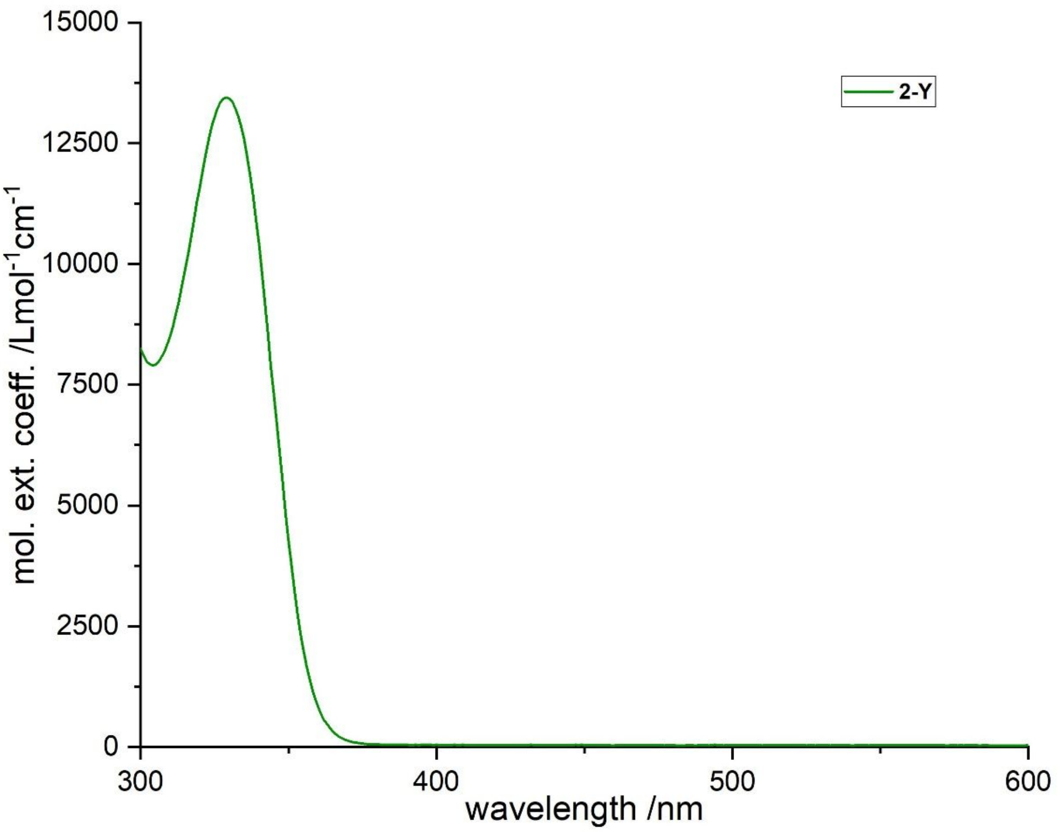

Figure S32. UV-vis spectrum of **2-Y** in toluene.

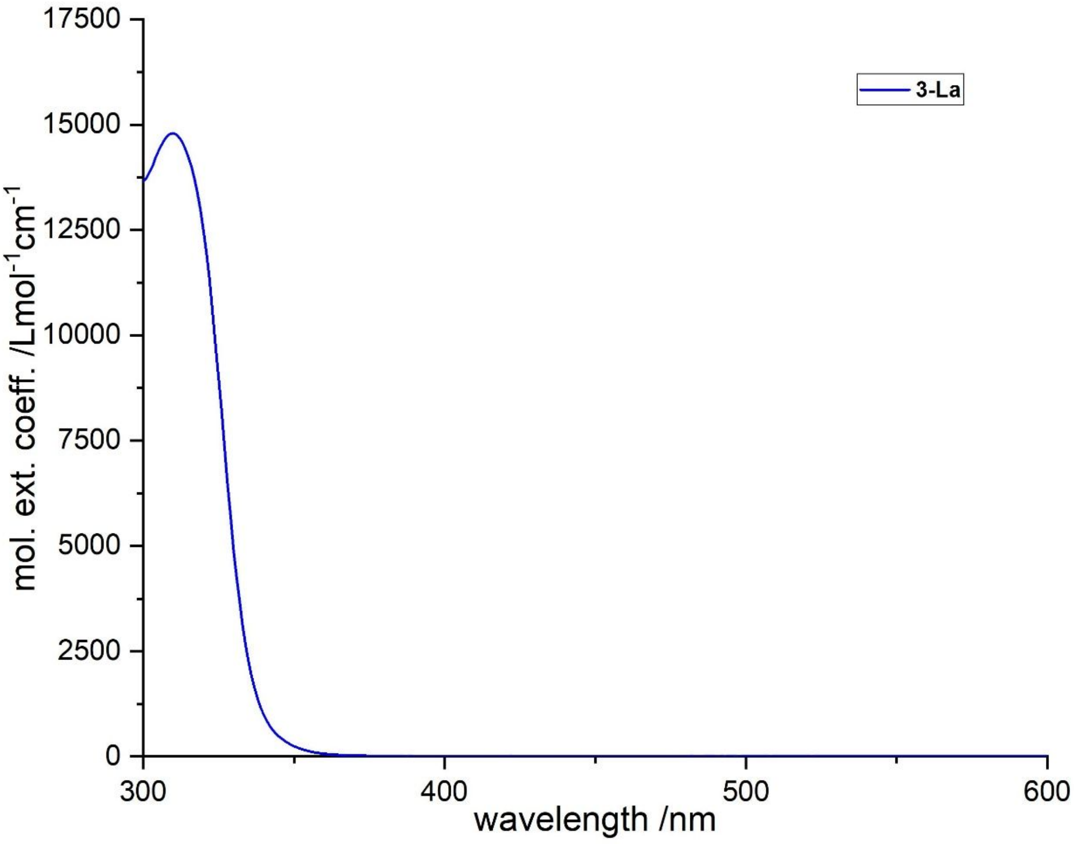

Figure S33. UV-vis spectrum of **3-La** in toluene.

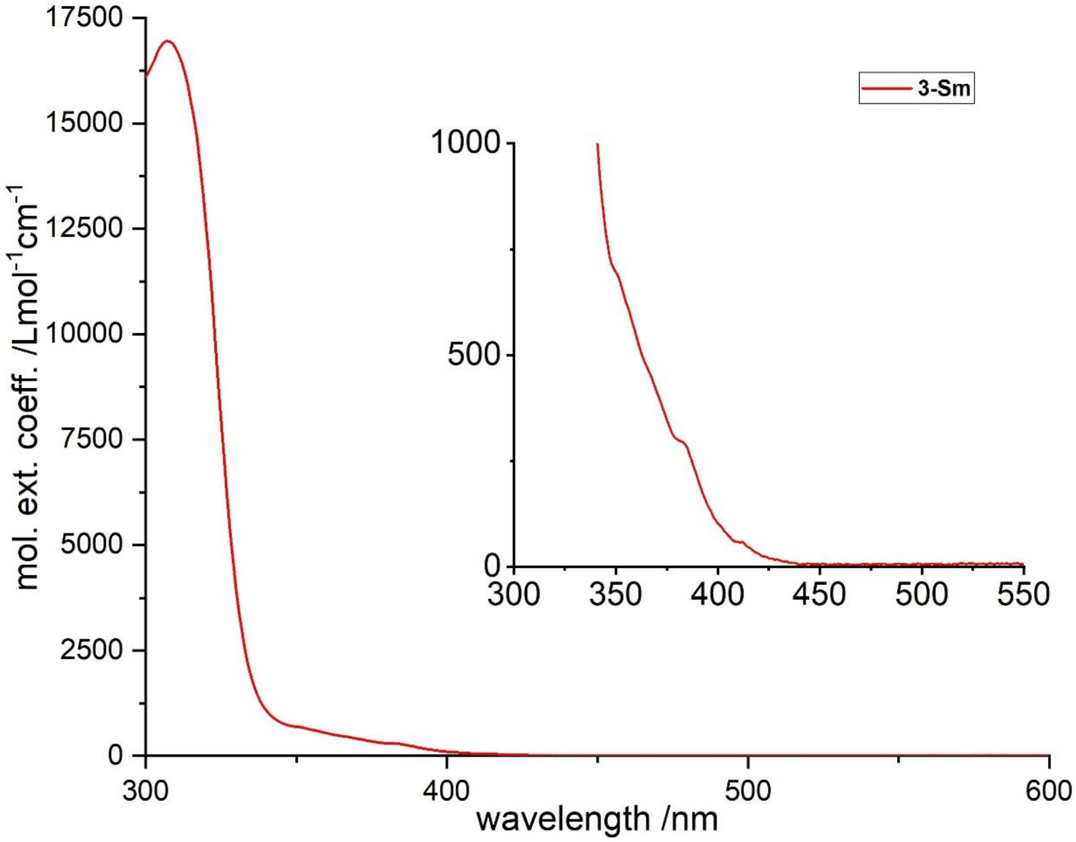

Figure S34. UV-vis spectrum of **3-Sm** in toluene.

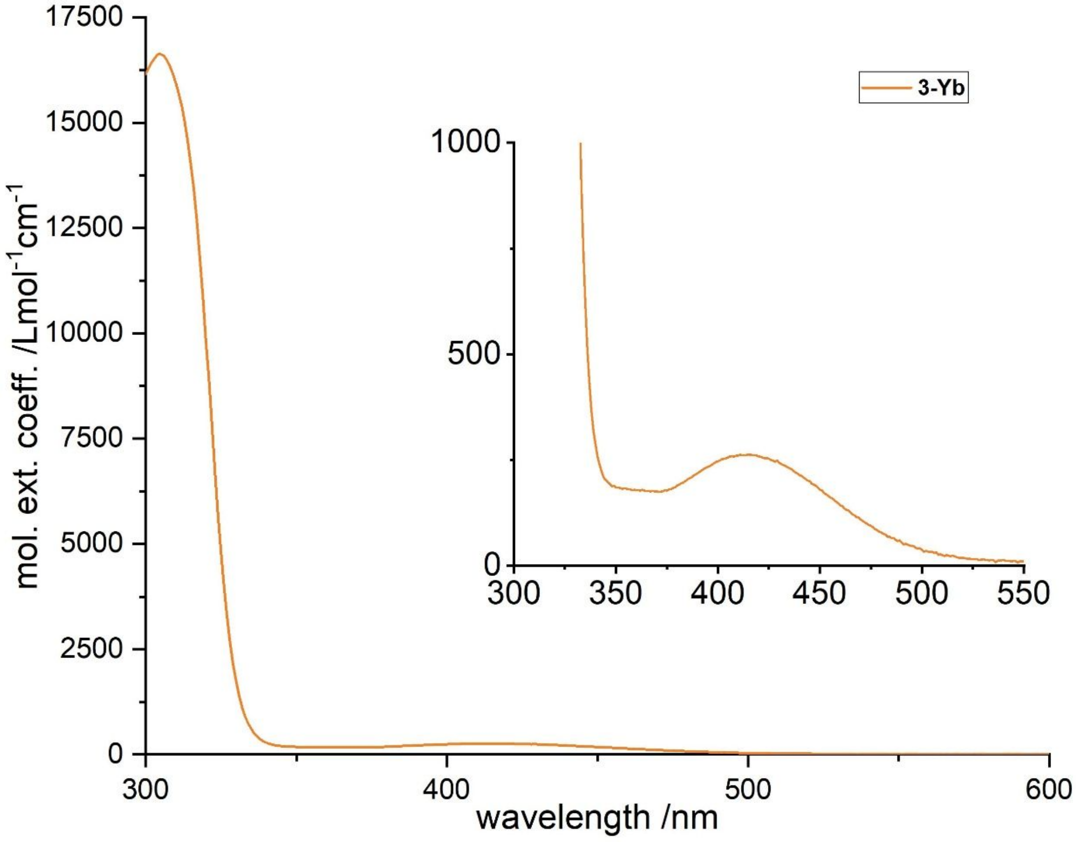

Figure S35. UV-vis spectrum of **3-Yb** in toluene.

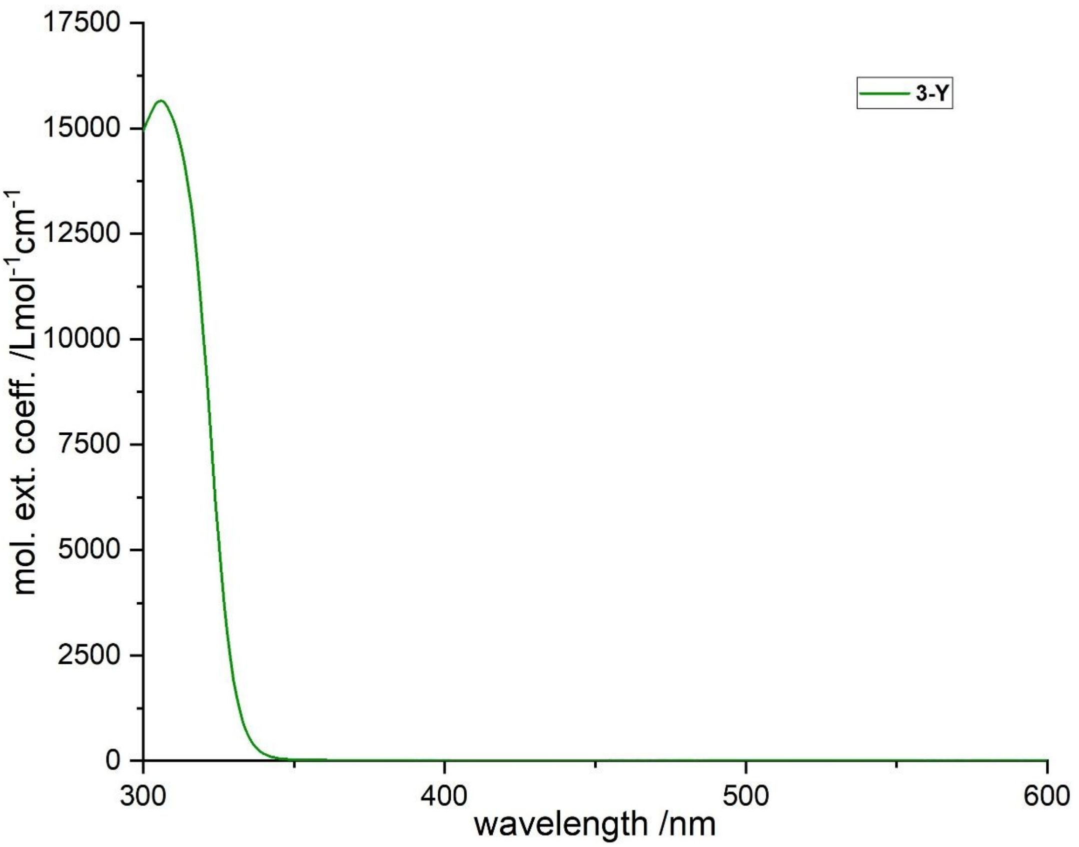

Figure S36. UV-vis spectrum of **3-Y** in toluene.

IR Spectroscopy:

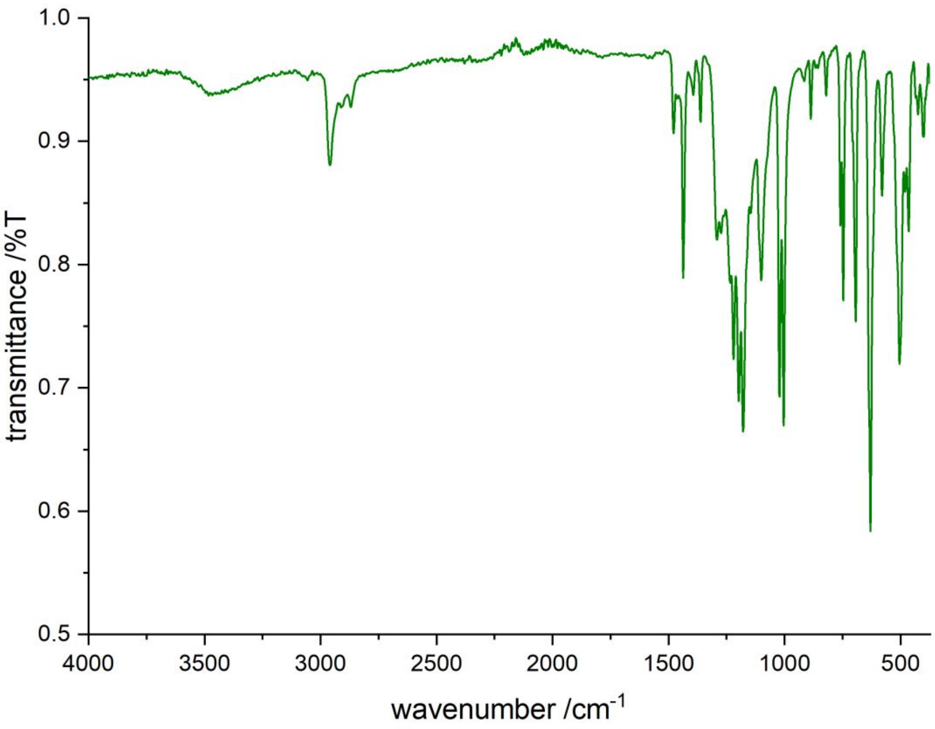

Figure S37. IR spectrum of **1** recorded neat from crystalline material using ATR.

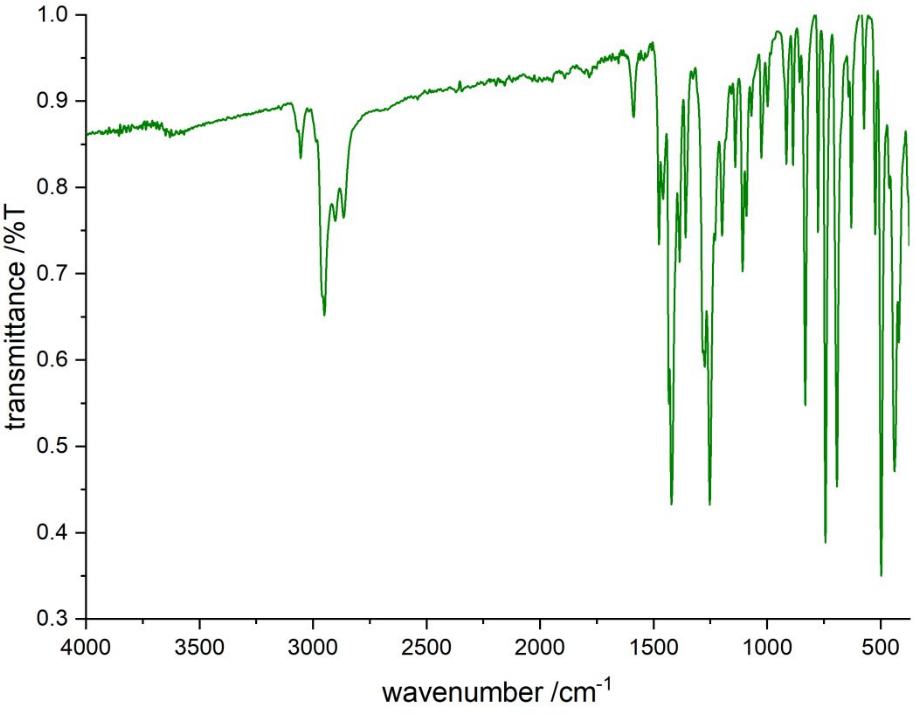

Figure S38. IR spectrum of **2-La** recorded neat from crystalline material using ATR.

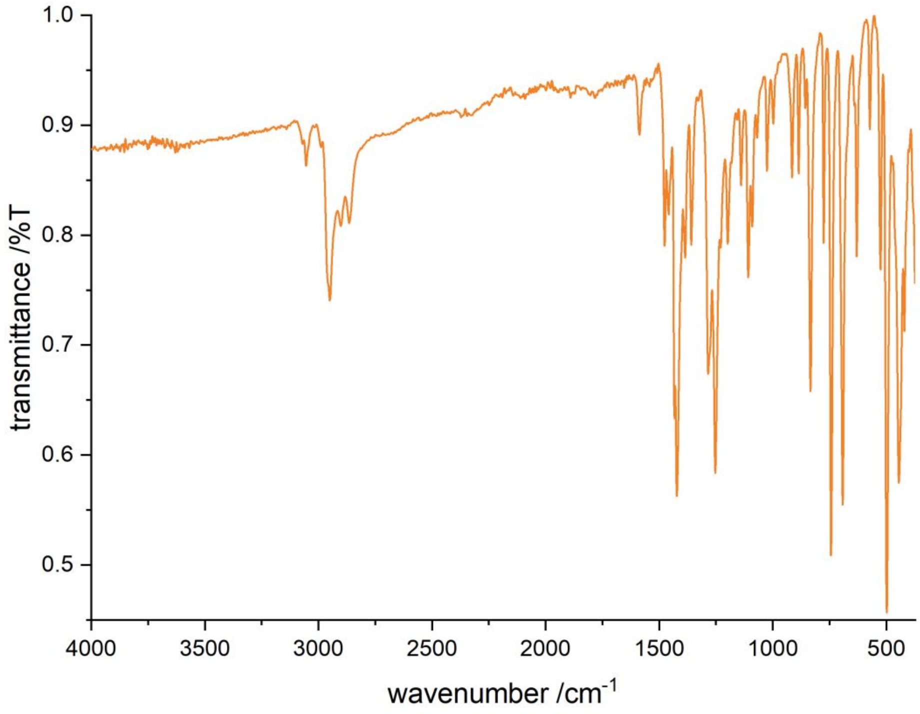

Figure S39. IR spectrum of **2-Sm** recorded neat from crystalline material using ATR.

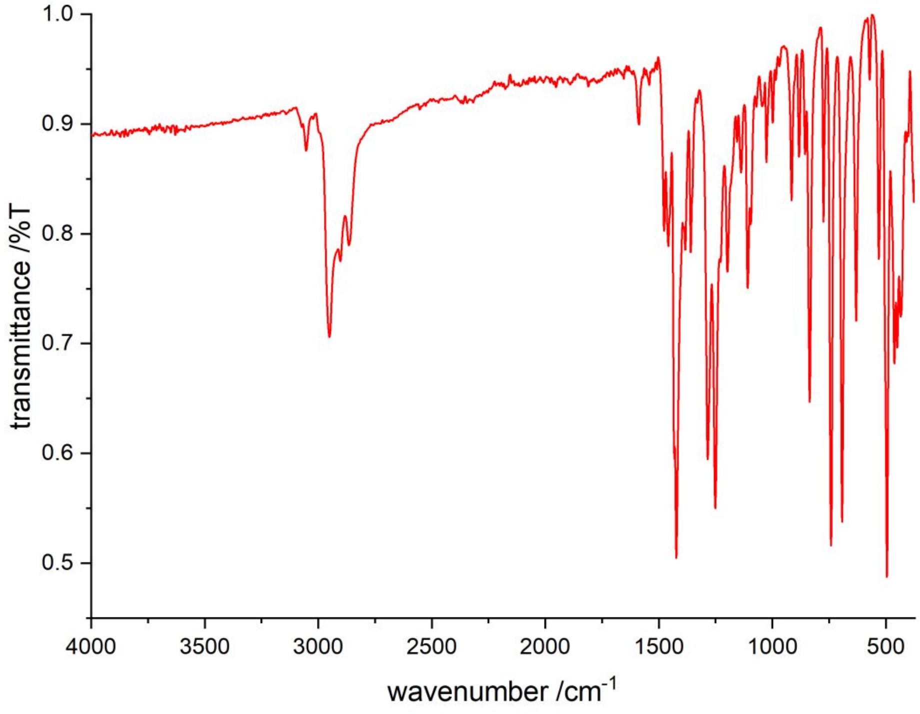

Figure S40. IR spectrum of **2-Yb** recorded neat from crystalline material using ATR.

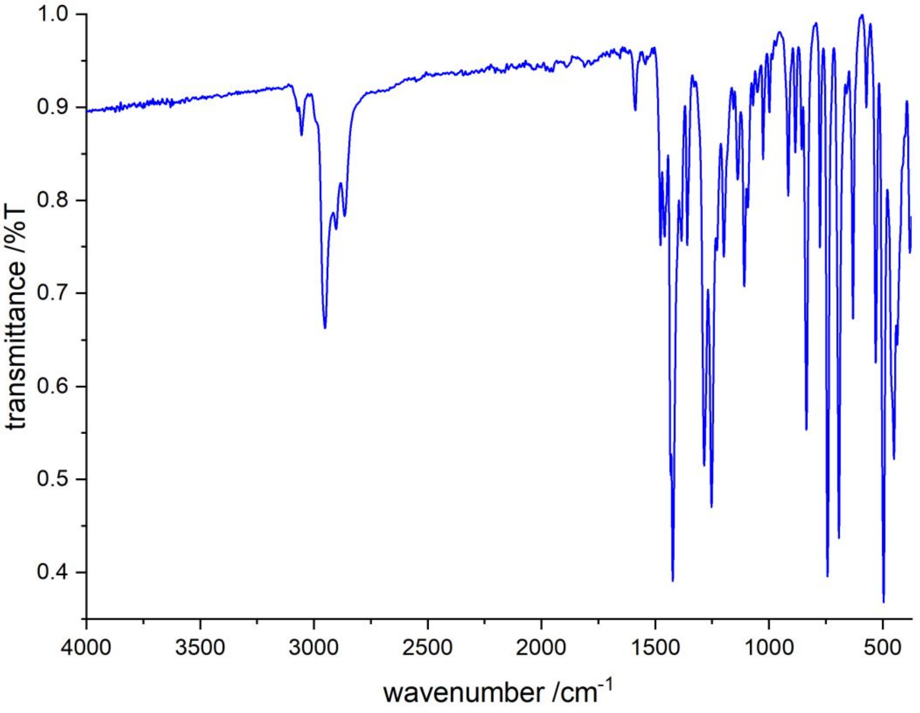

Figure S41. IR spectrum of **2-Y** recorded neat from crystalline material using ATR.

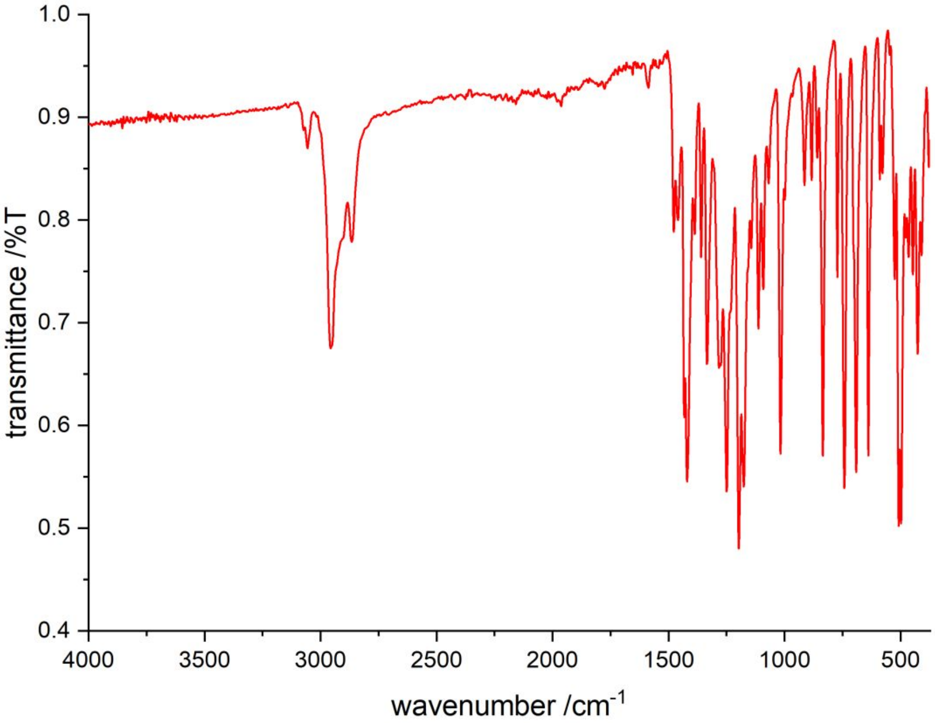

Figure S42. IR spectrum of **3-La** recorded neat from crystalline material using ATR.

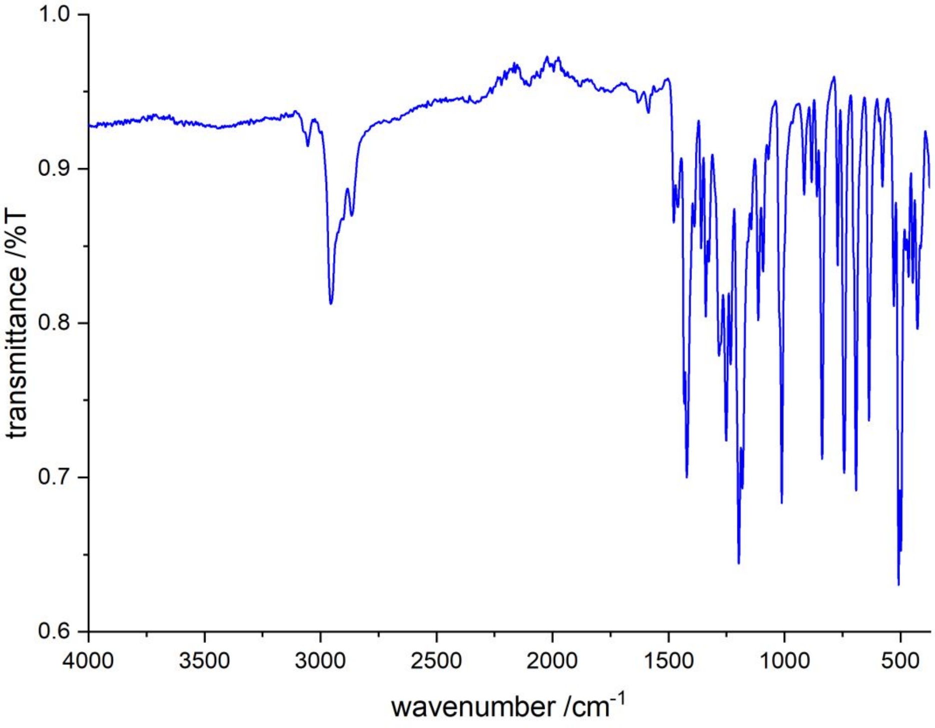

Figure S43. IR spectrum of **3-Sm** recorded neat from crystalline material using ATR.

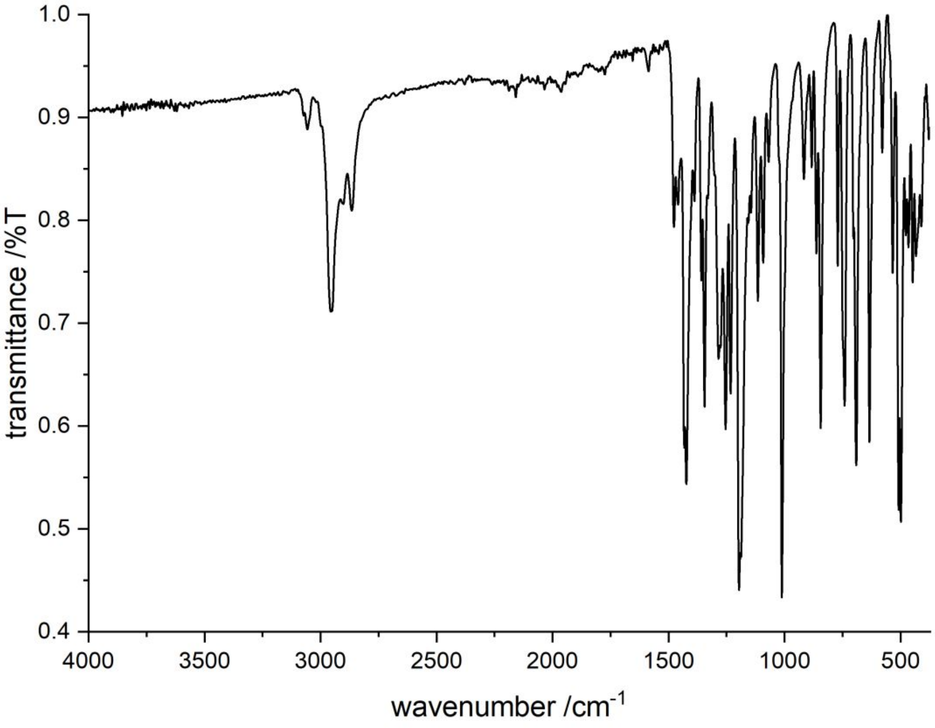

Figure S44. IR spectrum of **3-Yb** recorded neat from crystalline material using ATR.

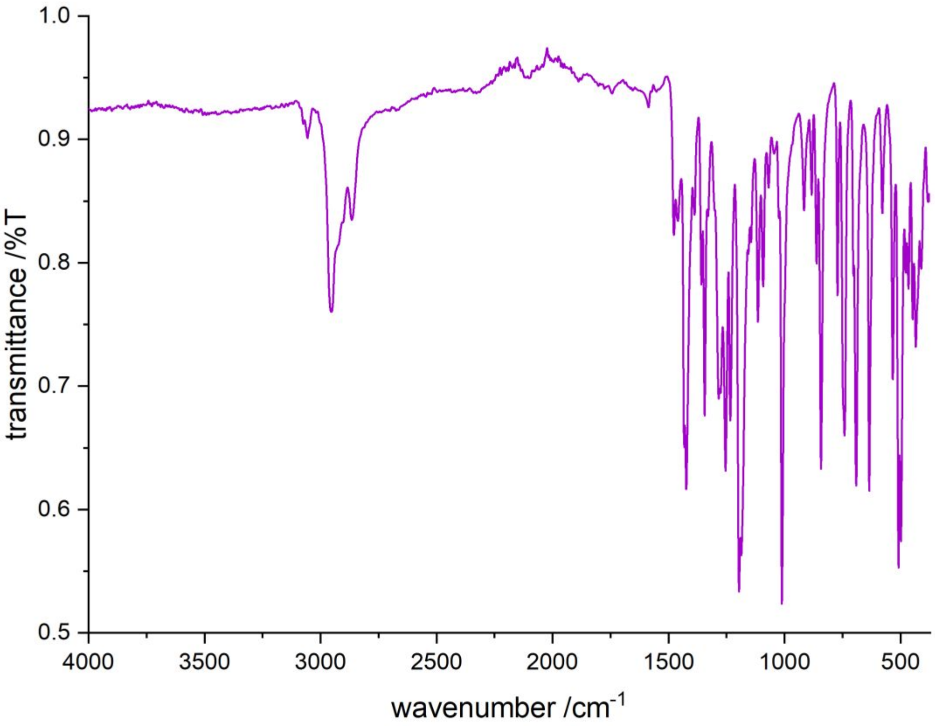

Figure S45. IR spectrum of **3-Y** recorded neat from crystalline material using ATR.

Computational Studies:

The computational study was performed in ORCA 5.0.3. Geometries of the crystal structures were used. The following input was used for **3-Y**, for **3-La** the basis section was changed accordingly.

```
! RKS PBE SP ZORA ZORA-def2-SVP def2/J D3 RIJCOSX KDIIS VerySlowConv
LARGEPRINT NOTRAH
%scf
      MaxIter 1000
      CNVDIIS 1
      CNVSOSCF 1
end

%basis
      newgto Y "SARC-ZORA-TZVPP" end
      newauxgto Y "SARC/J" end
      newgto Ag "SARC-ZORA-TZVPP" end
      newauxgto Ag "SARC/J" end
      delECP Y
      delECP Ag
end
```

Localized molecular orbitals were calculated using the orca_loc tool included in ORCA 5.0.3. The NEW-BOYS algorithm was used to calculate Foster-Boys orbitals and IAO-IBO was used for IBOs. Core orbitals were excluded from the analysis, otherwise all occupied orbitals were included. Below is an example input file for the Pipek-Mezey analysis, analysis for NEW-BOYS and IAO-IBO was conducted by changing the localization method while keeping the other settings.

```
input.gbw # input orbitals
output.loc.gbw # output orbitals
127 # orbital window: first orbital to be localized e.g. first
active
392 # orbital window: last orbital to be localized e.g. last active
1 # localization method: 1=PIPEK-MEZEY,2=FOSTER-BOYS,3=IAO-
IBO,4=IAO-BOYS,5=NEW-BOYS,6=AHFB
0 # operator: 0 for alpha, 1 for beta
1000 # maximum number of iterations
1e-6 # convergence tolerance of the localization functional value
0.0 # relative convergence tolerance of the localization functional
value
0.95 # printing thresh to call an orbital strongly localized
0.85 # printing thresh to call an orbital bond-like
1 # printlevel
1 # use Cholesky Decomposition (0=false, 1=true)
1 # randomize seed for localization (0=false, 1=true)
```

Following are the results of the localized molecular orbitals. Bonds between Ag, La, or Y and other atoms are highlighted in bold. ORCA counts atoms and orbitals starting at 0.

Pipek-Mezey analysis for 3-La

```
------------------------------------------------------------------------------
                           ORCA ORBITAL LOCALIZATION
------------------------------------------------------------------------------

Input orbitals are from                  ... .\La.gbw
Output orbitals are to                   ... .\La.loc
Max. number of iterations                ... 1000
Localizations seeded randomly            ... on
Convergence tolerance                    ... 1.000e-06
Treshold for strong local MOs            ... 9.500e-01
Treshold for bond MOs                    ... 8.500e-01
Operator                                 ... 0
Orbital range for localization           ... 131 to 401
Localization criterion                   ... PIPEK-MEZEY
Entering Jacobi type localization:
Using Cholesky decomposition as initial guess.
Initial value of the localization sum :   111.087994
ITERATION   0 : L=  157.8267982398 DL=  4.67e+01 (MAX-T)=  44.798
ITERATION   1 : L=  159.4578793160 DL=  1.63e+00 (MAX-T)=  40.829
ITERATION   2 : L=  159.4690706296 DL=  1.12e-02 (MAX-T)=  16.605
ITERATION   3 : L=  159.4718035100 DL=  2.73e-03 (MAX-T)=   5.800
ITERATION   4 : L=  159.4746674723 DL=  2.86e-03 (MAX-T)=   2.248
ITERATION   5 : L=  159.4811494882 DL=  6.48e-03 (MAX-T)=   4.245
ITERATION   6 : L=  159.4891274002 DL=  7.98e-03 (MAX-T)=   4.656
ITERATION   7 : L=  159.5013230582 DL=  1.22e-02 (MAX-T)=   5.158
ITERATION   8 : L=  159.5095534756 DL=  8.23e-03 (MAX-T)=   3.560
ITERATION   9 : L=  159.5139354250 DL=  4.38e-03 (MAX-T)=   2.538
ITERATION  10 : L=  159.5168568917 DL=  2.92e-03 (MAX-T)=   2.390
ITERATION  11 : L=  159.5193396178 DL=  2.48e-03 (MAX-T)=   1.898
ITERATION  12 : L=  159.5214193966 DL=  2.08e-03 (MAX-T)=   1.745
ITERATION  13 : L=  159.5228940236 DL=  1.47e-03 (MAX-T)=   1.902
ITERATION  14 : L=  159.5243827963 DL=  1.49e-03 (MAX-T)=   1.752
ITERATION  15 : L=  159.5256094822 DL=  1.23e-03 (MAX-T)=   1.445
ITERATION  16 : L=  159.5266300438 DL=  1.02e-03 (MAX-T)=   1.278
ITERATION  17 : L=  159.5271947025 DL=  5.65e-04 (MAX-T)=   1.124
ITERATION  18 : L=  159.5275334923 DL=  3.39e-04 (MAX-T)=   1.236
ITERATION  19 : L=  159.5279410112 DL=  4.08e-04 (MAX-T)=   0.850
ITERATION  20 : L=  159.5281423052 DL=  2.01e-04 (MAX-T)=   0.932
ITERATION  21 : L=  159.5284119966 DL=  2.70e-04 (MAX-T)=   0.676
ITERATION  22 : L=  159.5285861793 DL=  1.74e-04 (MAX-T)=   0.665
ITERATION  23 : L=  159.5286701379 DL=  8.40e-05 (MAX-T)=   0.499
ITERATION  24 : L=  159.5287757693 DL=  1.06e-04 (MAX-T)=   0.482
ITERATION  25 : L=  159.5288463594 DL=  7.06e-05 (MAX-T)=   0.389
ITERATION  26 : L=  159.5288926986 DL=  4.63e-05 (MAX-T)=   0.297
ITERATION  27 : L=  159.5289314123 DL=  3.87e-05 (MAX-T)=   0.249
ITERATION  28 : L=  159.5289489714 DL=  1.76e-05 (MAX-T)=   0.240
ITERATION  29 : L=  159.5289661232 DL=  1.72e-05 (MAX-T)=   0.212
ITERATION  30 : L=  159.5289790770 DL=  1.30e-05 (MAX-T)=   0.142
ITERATION  31 : L=  159.5289857053 DL=  6.63e-06 (MAX-T)=   0.118
ITERATION  32 : L=  159.5289915052 DL=  5.80e-06 (MAX-T)=   0.120
ITERATION  33 : L=  159.5289940114 DL=  2.51e-06 (MAX-T)=   0.105
ITERATION  34 : L=  159.5289970485 DL=  3.04e-06 (MAX-T)=   0.090
ITERATION  35 : L=  159.5289991425 DL=  2.09e-06 (MAX-T)=   0.060
ITERATION  36 : L=  159.5290007229 DL=  1.58e-06 (MAX-T)=   0.056
ITERATION  37 : L=  159.5290014481 DL=  7.25e-07 (MAX-T)=   0.046
LOCALIZATION SUM CONVERGED


-------------------------------------------------------------------------------
                    LOCALIZED MOLECULAR ORBITAL COMPOSITIONS
-------------------------------------------------------------------------------

The Mulliken populations for each LMO on each atom are computed
The LMO`s will be ordered according to atom index and type
  (A) Strongly localized MO`s have populations of >=0.950 on one atom
  (B) Two center bond orbitals have populations of >=0.850 on two atoms
  (C) Other MO`s are considered to be `delocalized`

FOUND  -  36 strongly local MO`s
       - 208 two center bond MO`s
       -  27 significantly delocalized MO`s

Rather strongly localized orbitals:
MO 166:  14O  -   0.960740
MO 165:  13O  -   0.960293
MO 164:  12O  -   0.960899
MO 163:  11O  -   1.022721
MO 162:  10O  -   1.000450
MO 161:   9O  -   1.000195
```

```
MO 160:   8F  -   1.013699
MO 159:   7F  -   1.015288
MO 158:   6F  -   1.016592
MO 157:   4P  -   0.999906
MO 156:   4P  -   1.000101
MO 155:   4P  -   1.003762
MO 154:   3P  -   1.002731
MO 153:   3P  -   1.000073
MO 152:   3P  -   0.999825
MO 151:   2P  -   1.000177
MO 150:   2P  -   0.999853
MO 149:   2P  -   1.002626
MO 148:   1La -   0.999643
MO 147:   1La -   1.000001
MO 146:   1La -   0.999934
MO 145:   1La -   0.999843
MO 144:   1La -   0.999632
MO 143:   1La -   1.043911
MO 142:   1La -   1.007243
MO 141:   1La -   1.006379
MO 140:   1La -   1.002335
MO 139:   0Ag -   0.977633
MO 138:   0Ag -   1.002556
MO 137:   0Ag -   1.029897
MO 136:   0Ag -   0.977778
MO 135:   0Ag -   0.992374
MO 134:   0Ag -   0.998164
MO 133:   0Ag -   1.001878
MO 132:   0Ag -   1.002731
MO 131:   0Ag -   0.992347
Bond-like localized orbitals:
MO 374: 183H  -   0.496792  and  37C  -   0.544565
MO 373: 182H  -   0.555165  and 175C  -   0.424648
MO 372: 181H  -   0.491693  and 178C  -   0.505568
MO 371: 180H  -   0.480992  and 178C  -   0.523067
MO 370: 179H  -   0.489631  and 178C  -   0.514835
MO 369: 178C  -   0.507102  and  79C  -   0.536771
MO 368: 177H  -   0.493377  and 175C  -   0.502760
MO 367: 176H  -   0.488551  and 175C  -   0.507589
MO 366: 175C  -   0.511960  and  79C  -   0.535492
MO 365: 174H  -   0.490933  and 171C  -   0.510496
MO 364: 173H  -   0.486302  and 171C  -   0.514861
MO 363: 172H  -   0.485396  and 171C  -   0.516928
MO 362: 171C  -   0.497226  and  79C  -   0.541913
MO 361: 170H  -   0.487412  and 167C  -   0.509896
MO 360: 169H  -   0.488838  and 167C  -   0.504996
MO 359: 168H  -   0.486008  and 167C  -   0.510691
MO 358: 167C  -   0.505800  and 117C  -   0.535198
MO 357: 166H  -   0.475982  and 163C  -   0.515797
MO 356: 165H  -   0.458685  and 163C  -   0.526816
MO 355: 164H  -   0.487598  and 163C  -   0.504021
MO 354: 163C  -   0.475939  and 117C  -   0.557705
MO 353: 162H  -   0.452548  and 159C  -   0.524616
MO 352: 161H  -   0.469666  and 159C  -   0.515238
MO 351: 160H  -   0.484842  and 159C  -   0.497047
MO 350: 159C  -   0.469168  and 124C  -   0.560564
MO 349: 158H  -   0.491234  and 155C  -   0.509840
MO 348: 157H  -   0.482938  and 155C  -   0.521762
MO 347: 156H  -   0.487166  and 155C  -   0.514970
MO 346: 155C  -   0.502716  and 133C  -   0.541036
MO 345: 154H  -   0.488058  and 151C  -   0.507618
MO 344: 153H  -   0.482918  and 151C  -   0.514016
MO 343: 152H  -   0.491388  and 151C  -   0.505046
MO 342: 151C  -   0.499439  and 133C  -   0.547905
MO 341: 150H  -   0.511048  and 149C  -   0.526139
MO 340: 149C  -   0.516331  and  95C  -   0.503893
MO 339: 149C  -   0.518605  and  91C  -   0.502290
MO 338: 148H  -   0.485936  and 145C  -   0.510978
MO 337: 147H  -   0.484602  and 145C  -   0.511948
MO 336: 146H  -   0.491667  and 145C  -   0.506037
MO 335: 145C  -   0.501729  and 108C  -   0.547102
MO 334: 144H  -   0.512644  and 143C  -   0.523224
MO 333: 143C  -   0.508321  and 139C  -   0.512927
MO 332: 143C  -   0.527560  and 106C  -   0.487792
MO 331: 142H  -   0.512474  and 141C  -   0.525109
MO 330: 141C  -   0.505008  and 139C  -   0.515321
MO 329: 141C  -   0.518151  and 122C  -   0.497274
MO 328: 140H  -   0.512784  and 139C  -   0.527321
MO 327: 138C  -   0.347599  and   8F  -   0.664015
MO 326: 138C  -   0.336203  and   7F  -   0.667131
MO 325: 138C  -   0.337478  and   6F  -   0.666781
MO 324: 138C  -   0.489989  and   5S  -   0.625740
```

MO 323:	137H	-	0.489728	and	134C	-	0.513047
MO 322:	136H	-	0.490408	and	134C	-	0.510165
MO 321:	135H	-	0.485174	and	134C	-	0.516080
MO 320:	134C	-	0.505949	and	133C	-	0.534170
MO 319:	133C	-	0.543128	and	85C	-	0.496466
MO 318:	132H	-	0.489769	and	129C	-	0.505633
MO 317:	131H	-	0.486282	and	129C	-	0.509892
MO 316:	130H	-	0.488552	and	129C	-	0.506839
MO 315:	129C	-	0.506856	and	124C	-	0.532969
MO 314:	128H	-	0.490138	and	125C	-	0.502411
MO 313:	127H	-	0.468636	and	125C	-	0.525513
MO 312:	126H	-	0.445638	and	125C	-	0.541685
MO 311:	125C	-	0.468493	and	124C	-	0.563104
MO 310:	124C	-	0.524011	and	86C	-	0.512830
MO 309:	123H	-	0.519956	and	122C	-	0.517717
MO 308:	122C	-	0.489109	and	105C	-	0.523162
MO 307:	121H	-	0.486144	and	118C	-	0.498239
MO 306:	120H	-	0.451036	and	118C	-	0.525471
MO 305:	119H	-	0.464522	and	118C	-	0.521104
MO 304:	118C	-	0.465373	and	117C	-	0.561777
MO 303:	117C	-	0.523608	and	104C	-	0.513732
MO 302:	116H	-	0.490956	and	113C	-	0.507422
MO 301:	115H	-	0.482651	and	113C	-	0.520224
MO 300:	114H	-	0.486563	and	113C	-	0.516854
MO 299:	113C	-	0.505549	and	108C	-	0.532934
MO 298:	112H	-	0.484264	and	109C	-	0.519730
MO 297:	111H	-	0.491058	and	109C	-	0.510002
MO 296:	110H	-	0.487886	and	109C	-	0.514536
MO 295:	109C	-	0.502339	and	108C	-	0.540799
MO 294:	108C	-	0.544306	and	103C	-	0.495641
MO 293:	107H	-	0.502900	and	106C	-	0.542039
MO 292:	106C	-	0.498262	and	105C	-	0.517483
MO 291:	105C	-	0.551549	and	2P	-	0.451455
MO 290:	104C	-	0.471797	and	101C	-	0.545377
MO 289:	104C	-	0.523811	and	97C	-	0.514752
MO 288:	103C	-	0.529230	and	101C	-	0.494587
MO 287:	103C	-	0.504969	and	99C	-	0.526873
MO 286:	102H	-	0.527199	and	101C	-	0.526900
MO 285:	100H	-	0.513953	and	99C	-	0.537858
MO 284:	99C	-	0.481917	and	98C	-	0.513063
MO 283:	98C	-	0.563632	and	97C	-	0.466852
MO 282:	98C	-	0.506214	and	2P	-	0.492272
MO 281:	97C	-	0.379271	and	14O	-	0.606069
MO 280:	96H	-	0.511279	and	95C	-	0.528047
MO 279:	95C	-	0.521343	and	93C	-	0.495406
MO 278:	94H	-	0.502816	and	93C	-	0.540078
MO 277:	93C	-	0.505476	and	88C	-	0.507666
MO 276:	92H	-	0.512743	and	91C	-	0.525196
MO 275:	91C	-	0.523222	and	89C	-	0.489122
MO 274:	90H	-	0.518618	and	89C	-	0.525481
MO 273:	89C	-	0.499277	and	88C	-	0.518911
MO 272:	88C	-	0.556457	and	3P	-	0.454180
MO 271:	87C	-	0.535290	and	86C	-	0.506838
MO 270:	87C	-	0.454391	and	80C	-	0.574461
MO 269:	87C	-	0.375109	and	13O	-	0.608076
MO 268:	86C	-	0.491668	and	83C	-	0.523951
MO 267:	85C	-	0.536849	and	83C	-	0.490891
MO 266:	85C	-	0.505430	and	81C	-	0.522435
MO 265:	84H	-	0.523924	and	83C	-	0.530654
MO 264:	82H	-	0.519680	and	81C	-	0.531318
MO 263:	81C	-	0.503499	and	80C	-	0.494039
MO 262:	80C	-	0.511842	and	4P	-	0.491539
MO 261:	79C	-	0.541902	and	74C	-	0.498699
MO 260:	78C	-	0.457516	and	77C	-	0.576068
MO 259:	78C	-	0.522407	and	71C	-	0.520383
MO 258:	78C	-	0.381953	and	12O	-	0.604836
MO 257:	77C	-	0.514900	and	75C	-	0.480860
MO 256:	77C	-	0.509901	and	3P	-	0.490422
MO 255:	76H	-	0.537047	and	75C	-	0.514534
MO 254:	75C	-	0.528215	and	74C	-	0.505402
MO 253:	74C	-	0.527678	and	72C	-	0.496929
MO 252:	73H	-	0.525802	and	72C	-	0.528797
MO 251:	72C	-	0.539412	and	71C	-	0.477033
MO 250:	71C	-	0.510328	and	58C	-	0.529150
MO 249:	70H	-	0.490037	and	67C	-	0.506070
MO 248:	69H	-	0.487958	and	67C	-	0.508912
MO 247:	68H	-	0.486622	and	67C	-	0.507784
MO 246:	67C	-	0.506636	and	58C	-	0.536711
MO 245:	66H	-	0.450302	and	63C	-	0.532479
MO 244:	65H	-	0.477848	and	63C	-	0.509130
MO 243:	64H	-	0.475973	and	63C	-	0.507565
MO 242:	63C	-	0.476633	and	58C	-	0.552390

MO 241:	62H	-	0.487803	and	59C	-	0.503510
MO 240:	61H	-	0.482924	and	59C	-	0.509945
MO 239:	60H	-	0.431439	and	59C	-	0.554602
MO 238:	59C	-	0.467431	and	58C	-	0.563156
MO 237:	57H	-	0.511408	and	56C	-	0.526565
MO 236:	56C	-	0.523536	and	54C	-	0.496073
MO 235:	56C	-	0.511654	and	52C	-	0.508675
MO 234:	55H	-	0.514262	and	54C	-	0.527787
MO 233:	54C	-	0.527607	and	50C	-	0.484938
MO 232:	53H	-	0.511425	and	52C	-	0.524686
MO 231:	52C	-	0.517481	and	48C	-	0.498618
MO 230:	51H	-	0.524244	and	50C	-	0.529003
MO 229:	50C	-	0.491101	and	47C	-	0.534296
MO 228:	49H	-	0.510869	and	48C	-	0.532537
MO 227:	48C	-	0.504576	and	47C	-	0.503584
MO 226:	47C	-	0.537017	and	2P	-	0.470988
MO 225:	46H	-	0.509613	and	45C	-	0.526276
MO 224:	45C	-	0.505896	and	43C	-	0.514877
MO 223:	45C	-	0.528941	and	37C	-	0.487358
MO 222:	44H	-	0.512145	and	43C	-	0.525888
MO 221:	43C	-	0.517711	and	41C	-	0.502661
MO 220:	42H	-	0.518819	and	41C	-	0.519104
MO 219:	41C	-	0.522095	and	39C	-	0.489907
MO 218:	40H	-	0.506567	and	39C	-	0.537955
MO 217:	39C	-	0.498843	and	38C	-	0.520983
MO 216:	38C	-	0.517791	and	37C	-	0.494528
MO 215:	38C	-	0.535367	and	3P	-	0.477237
MO 214:	36H	-	0.510981	and	35C	-	0.527141
MO 213:	35C	-	0.517950	and	33C	-	0.503282
MO 212:	35C	-	0.515170	and	29C	-	0.506001
MO 211:	34H	-	0.511356	and	33C	-	0.525231
MO 210:	33C	-	0.521309	and	27C	-	0.493074
MO 209:	32H	-	0.499163	and	31C	-	0.541009
MO 208:	31C	-	0.489941	and	29C	-	0.526643
MO 207:	31C	-	0.504863	and	26C	-	0.511133
MO 206:	30H	-	0.510626	and	29C	-	0.526183
MO 205:	28H	-	0.509285	and	27C	-	0.523349
MO 204:	27C	-	0.493908	and	26C	-	0.521524
MO 203:	26C	-	0.562284	and	4P	-	0.445349
MO 202:	25H	-	0.494468	and	24C	-	0.540977
MO 201:	24C	-	0.488581	and	22C	-	0.525909
MO 200:	24C	-	0.487157	and	15C	-	0.525061
MO 199:	23H	-	0.508164	and	22C	-	0.526190
MO 198:	22C	-	0.505276	and	20C	-	0.515138
MO 197:	21H	-	0.511022	and	20C	-	0.528106
MO 196:	20C	-	0.513917	and	18C	-	0.505913
MO 195:	19H	-	0.516802	and	18C	-	0.522977
MO 194:	18C	-	0.517043	and	16C	-	0.495227
MO 193:	17H	-	0.518551	and	16C	-	0.528403
MO 192:	16C	-	0.495169	and	15C	-	0.520804
MO 191:	15C	-	0.536464	and	4P	-	0.467248
**MO 190:**	**14O**	**-**	**0.872151**	**and**	**1La**	**-**	**0.082376**
**MO 189:**	**14O**	**-**	**0.837468**	**and**	**0Ag**	**-**	**0.051846**
**MO 188:**	**13O**	**-**	**0.875432**	**and**	**1La**	**-**	**0.088432**
**MO 187:**	**13O**	**-**	**0.835694**	**and**	**0Ag**	**-**	**0.057367**
**MO 186:**	**12O**	**-**	**0.872986**	**and**	**1La**	**-**	**0.086055**
**MO 185:**	**12O**	**-**	**0.837280**	**and**	**0Ag**	**-**	**0.057524**
MO 184:	11O	-	0.513879	and	5S	-	0.487294
MO 183:	11O	-	0.824200	and	5S	-	0.177955
MO 182:	11O	-	0.821090	and	5S	-	0.150456
MO 181:	10O	-	0.561417	and	5S	-	0.436259
MO 180:	10O	-	0.833179	and	5S	-	0.122731
MO 179:	10O	-	0.837567	and	5S	-	0.113469
MO 178:	9O	-	0.835811	and	5S	-	0.117018
MO 177:	9O	-	0.845505	and	5S	-	0.109761
MO 176:	9O	-	0.560591	and	5S	-	0.436532
**MO 175:**	**8F**	**-**	**0.932658**	**and**	**1La**	**-**	**0.024637**
**MO 174:**	**8F**	**-**	**0.932896**	**and**	**1La**	**-**	**0.016205**
**MO 173:**	**7F**	**-**	**0.930857**	**and**	**1La**	**-**	**0.010537**
**MO 172:**	**7F**	**-**	**0.930823**	**and**	**1La**	**-**	**0.003322**
**MO 171:**	**6F**	**-**	**0.930925**	**and**	**1La**	**-**	**0.008658**
**MO 170:**	**6F**	**-**	**0.931498**	**and**	**1La**	**-**	**0.002266**
**MO 169:**	**4P**	**-**	**0.906810**	**and**	**0Ag**	**-**	**0.124300**
**MO 168:**	**3P**	**-**	**0.893605**	**and**	**0Ag**	**-**	**0.152441**
**MO 167:**	**2P**	**-**	**0.900092**	**and**	**0Ag**	**-**	**0.138256**

More delocalized orbitals:

MO 401: 20C - 0.301 22C - 0.483 24C - 0.152

MO 400: 81C - 0.259 83C - 0.187 85C - 0.497

MO 399: 105C - 0.466 106C - 0.350 122C - 0.091

MO 398: 83C - 0.268 86C - 0.493 87C - 0.186

MO 397: 122C - 0.362 139C - 0.098 141C - 0.454

MO 396: 93C - 0.184 95C - 0.492 149C - 0.270

```
MO 395:  71C - 0.482 72C - 0.330 78C - 0.135
MO 394:  80C - 0.555 81C - 0.193 87C - 0.178
MO 393:  97C - 0.111 101C - 0.365 104C - 0.469
MO 392:  15C - 0.510 16C - 0.141 24C - 0.278
MO 391:  27C - 0.376 33C - 0.451 35C - 0.083
MO 390:  72C - 0.129 74C - 0.482 75C - 0.323
MO 389:  50C - 0.142 54C - 0.476 56C - 0.315
MO 388:  37C - 0.301 43C - 0.146 45C - 0.487
MO 387:  88C - 0.510 89C - 0.169 93C - 0.248
MO 386:  99C - 0.355 101C - 0.098 103C - 0.471
MO 385:  89C - 0.271 91C - 0.493 149C - 0.174
MO 384:  48C - 0.316 52C - 0.482 56C - 0.136
MO 383:  37C - 0.138 38C - 0.509 39C - 0.281
MO 382:  106C - 0.101 139C - 0.359 143C - 0.459
MO 381:  39C - 0.154 41C - 0.481 43C - 0.302
MO 380:  75C - 0.138 77C - 0.559 78C - 0.222
MO 379:  29C - 0.453 31C - 0.087 35C - 0.377
MO 378:  97C - 0.242 98C - 0.555 99C - 0.115
MO 377:  16C - 0.303 18C - 0.481 20C - 0.148
MO 376:  26C - 0.468 27C - 0.079 31C - 0.360
MO 375:  47C - 0.507 48C - 0.125 50C - 0.289
```

Foster-Boys analysis for 3-La

```
------------------------------------------------------------------------------
                          ORCA ORBITAL LOCALIZATION
------------------------------------------------------------------------------

Input orbitals are from                  ... La.gbw
Output orbitals are to                   ... La_NewBoys.loc.gbw
Max. number of iterations                ... 1000
Localizations seeded randomly            ... on
Convergence tolerance                    ... 1.000e-06
Treshold for strong local MOs            ... 9.500e-01
Treshold for bond MOs                    ... 8.500e-01
Operator                                 ... 0
Orbital range for localization           ... 131 to 401
Localization criterion                   ... NEW-BOYS
Warning: cannot retrieve the overlap matrix S
        ... Overlap was succesfully recalculated
Doing the dipole integrals     ... o.k.
Initial value of the localization sum : 149840.880741
ITERATION   0 : L=151639.0303245130 DL=  1.80e+03 (AVERGE_DL)=    0.2216977976
ITERATION   1 : L=151935.8787487881 DL=  2.97e+02 (AVERGE_DL)=    0.0900774004
ITERATION   2 : L=151956.2711299208 DL=  2.04e+01 (AVERGE_DL)=    0.0236092635
ITERATION   3 : L=151959.4416235796 DL=  3.17e+00 (AVERGE_DL)=    0.0093091904
ITERATION   4 : L=151961.3216775948 DL=  1.88e+00 (AVERGE_DL)=    0.0071685882
ITERATION   5 : L=151962.1087508772 DL=  7.87e-01 (AVERGE_DL)=    0.0046382703
ITERATION   6 : L=151962.3469613846 DL=  2.38e-01 (AVERGE_DL)=    0.0025516960
ITERATION   7 : L=151962.4076152994 DL=  6.07e-02 (AVERGE_DL)=    0.0012875909
ITERATION   8 : L=151962.4261954622 DL=  1.86e-02 (AVERGE_DL)=    0.0007126450
ITERATION   9 : L=151962.4349819671 DL=  8.79e-03 (AVERGE_DL)=    0.0004900682
ITERATION  10 : L=151962.4416309882 DL=  6.65e-03 (AVERGE_DL)=    0.0004263118
ITERATION  11 : L=151962.4481332460 DL=  6.50e-03 (AVERGE_DL)=    0.0004215805
ITERATION  12 : L=151962.4551043013 DL=  6.97e-03 (AVERGE_DL)=    0.0004365136
ITERATION  13 : L=151962.4627949574 DL=  7.69e-03 (AVERGE_DL)=    0.0004584903
ITERATION  14 : L=151962.4713017459 DL=  8.51e-03 (AVERGE_DL)=    0.0004822045
ITERATION  15 : L=151962.4805575811 DL=  9.26e-03 (AVERGE_DL)=    0.0005029864
ITERATION  16 : L=151962.4902784426 DL=  9.72e-03 (AVERGE_DL)=    0.0005154670
ITERATION  17 : L=151962.4999834877 DL=  9.71e-03 (AVERGE_DL)=    0.0005150475
ITERATION  18 : L=151962.5091442912 DL=  9.16e-03 (AVERGE_DL)=    0.0005003976
ITERATION  19 : L=151962.5173783138 DL=  8.23e-03 (AVERGE_DL)=    0.0004744107
ITERATION  20 : L=151962.5245452656 DL=  7.17e-03 (AVERGE_DL)=    0.0004426044
ITERATION  21 : L=151962.5307075700 DL=  6.16e-03 (AVERGE_DL)=    0.0004104120
ITERATION  22 : L=151962.5360250050 DL=  5.32e-03 (AVERGE_DL)=    0.0003812410
ITERATION  23 : L=151962.5406660886 DL=  4.64e-03 (AVERGE_DL)=    0.0003561707
ITERATION  24 : L=151962.5447672589 DL=  4.10e-03 (AVERGE_DL)=    0.0003348130
ITERATION  25 : L=151962.5484264541 DL=  3.66e-03 (AVERGE_DL)=    0.0003162578
ITERATION  26 : L=151962.5517105493 DL=  3.28e-03 (AVERGE_DL)=    0.0002996100
ITERATION  27 : L=151962.5546653695 DL=  2.95e-03 (AVERGE_DL)=    0.0002841934
ITERATION  28 : L=151962.5573238969 DL=  2.66e-03 (AVERGE_DL)=    0.0002695684
ITERATION  29 : L=151962.5597119852 DL=  2.39e-03 (AVERGE_DL)=    0.0002554899
ITERATION  30 : L=151962.5618517490 DL=  2.14e-03 (AVERGE_DL)=    0.0002418418
ITERATION  31 : L=151962.5637634319 DL=  1.91e-03 (AVERGE_DL)=    0.0002285896
ITERATION  32 : L=151962.5654662714 DL=  1.70e-03 (AVERGE_DL)=    0.0002157423
ITERATION  33 : L=151962.5669788003 DL=  1.51e-03 (AVERGE_DL)=    0.0002033295
ITERATION  34 : L=151962.5683188977 DL=  1.34e-03 (AVERGE_DL)=    0.0001913889
ITERATION  35 : L=151962.5695036507 DL=  1.18e-03 (AVERGE_DL)=    0.0001799544
ITERATION  36 : L=151962.5705492209 DL=  1.05e-03 (AVERGE_DL)=    0.0001690539
ITERATION  37 : L=151962.5714707460 DL=  9.22e-04 (AVERGE_DL)=    0.0001587092
```

```
ITERATION  38 : L=151962.5722822586 DL=  8.12e-04 (AVERGE_DL)=    0.0001489348
ITERATION  39 : L=151962.5729966051 DL=  7.14e-04 (AVERGE_DL)=    0.0001397343
ITERATION  40 : L=151962.5736254849 DL=  6.29e-04 (AVERGE_DL)=    0.0001311089
ITERATION  41 : L=151962.5741795153 DL=  5.54e-04 (AVERGE_DL)=    0.0001230595
ITERATION  42 : L=151962.5746681754 DL=  4.89e-04 (AVERGE_DL)=    0.0001155718
ITERATION  43 : L=151962.5750999834 DL=  4.32e-04 (AVERGE_DL)=    0.0001086410
ITERATION  44 : L=151962.5754824826 DL=  3.82e-04 (AVERGE_DL)=    0.0001022501
ITERATION  45 : L=151962.5758224184 DL=  3.40e-04 (AVERGE_DL)=    0.0000963933
ITERATION  46 : L=151962.5761257132 DL=  3.03e-04 (AVERGE_DL)=    0.0000910502
ITERATION  47 : L=151962.5763976073 DL=  2.72e-04 (AVERGE_DL)=    0.0000862082
ITERATION  48 : L=151962.5766427172 DL=  2.45e-04 (AVERGE_DL)=    0.0000818519
ITERATION  49 : L=151962.5768651001 DL=  2.22e-04 (AVERGE_DL)=    0.0000779649
ITERATION  50 : L=151962.5770683111 DL=  2.03e-04 (AVERGE_DL)=    0.0000745284
ITERATION  51 : L=151962.5772555006 DL=  1.87e-04 (AVERGE_DL)=    0.0000715302
ITERATION  52 : L=151962.5774293754 DL=  1.74e-04 (AVERGE_DL)=    0.0000689393
ITERATION  53 : L=151962.5775923480 DL=  1.63e-04 (AVERGE_DL)=    0.0000667430
ITERATION  54 : L=151962.5777464974 DL=  1.54e-04 (AVERGE_DL)=    0.0000649112
ITERATION  55 : L=151962.5778936444 DL=  1.47e-04 (AVERGE_DL)=    0.0000634197
ITERATION  56 : L=151962.5780353545 DL=  1.42e-04 (AVERGE_DL)=    0.0000622370
ITERATION  57 : L=151962.5781730081 DL=  1.38e-04 (AVERGE_DL)=    0.0000613398
ITERATION  58 : L=151962.5783077561 DL=  1.35e-04 (AVERGE_DL)=    0.0000606890
ITERATION  59 : L=151962.5784406099 DL=  1.33e-04 (AVERGE_DL)=    0.0000602609
ITERATION  60 : L=151962.5785724060 DL=  1.32e-04 (AVERGE_DL)=    0.0000600205
ITERATION  61 : L=151962.5787038371 DL=  1.31e-04 (AVERGE_DL)=    0.0000599373
ITERATION  62 : L=151962.5788354791 DL=  1.32e-04 (AVERGE_DL)=    0.0000599854
ITERATION  63 : L=151962.5789677583 DL=  1.32e-04 (AVERGE_DL)=    0.0000601304
ITERATION  64 : L=151962.5791010219 DL=  1.33e-04 (AVERGE_DL)=    0.0000603537
ITERATION  65 : L=151962.5792354922 DL=  1.34e-04 (AVERGE_DL)=    0.0000606264
ITERATION  66 : L=151962.5793713079 DL=  1.36e-04 (AVERGE_DL)=    0.0000609289
ITERATION  67 : L=151962.5795085209 DL=  1.37e-04 (AVERGE_DL)=    0.0000612415
ITERATION  68 : L=151962.5796470834 DL=  1.39e-04 (AVERGE_DL)=    0.0000615420
ITERATION  69 : L=151962.5797869056 DL=  1.40e-04 (AVERGE_DL)=    0.0000618211
ITERATION  70 : L=151962.5799278041 DL=  1.41e-04 (AVERGE_DL)=    0.0000620586
ITERATION  71 : L=151962.5800695549 DL=  1.42e-04 (AVERGE_DL)=    0.0000622460
ITERATION  72 : L=151962.5802118834 DL=  1.42e-04 (AVERGE_DL)=    0.0000623727
ITERATION  73 : L=151962.5803544695 DL=  1.43e-04 (AVERGE_DL)=    0.0000624291
ITERATION  74 : L=151962.5804969521 DL=  1.42e-04 (AVERGE_DL)=    0.0000624064
ITERATION  75 : L=151962.5806389537 DL=  1.42e-04 (AVERGE_DL)=    0.0000623010
ITERATION  76 : L=151962.5807800930 DL=  1.41e-04 (AVERGE_DL)=    0.0000621116
ITERATION  77 : L=151962.5809199676 DL=  1.40e-04 (AVERGE_DL)=    0.0000618326
ITERATION  78 : L=151962.5810581846 DL=  1.38e-04 (AVERGE_DL)=    0.0000614652
ITERATION  79 : L=151962.5811943485 DL=  1.36e-04 (AVERGE_DL)=    0.0000610070
ITERATION  80 : L=151962.5813280862 DL=  1.34e-04 (AVERGE_DL)=    0.0000604610
ITERATION  81 : L=151962.5814590416 DL=  1.31e-04 (AVERGE_DL)=    0.0000598288
ITERATION  82 : L=151962.5815868933 DL=  1.28e-04 (AVERGE_DL)=    0.0000591156
ITERATION  83 : L=151962.5817113421 DL=  1.24e-04 (AVERGE_DL)=    0.0000583235
ITERATION  84 : L=151962.5818321278 DL=  1.21e-04 (AVERGE_DL)=    0.0000574588
ITERATION  85 : L=151962.5819490217 DL=  1.17e-04 (AVERGE_DL)=    0.0000565255
ITERATION  86 : L=151962.5820618384 DL=  1.13e-04 (AVERGE_DL)=    0.0000555310
ITERATION  87 : L=151962.5821704235 DL=  1.09e-04 (AVERGE_DL)=    0.0000544796
ITERATION  88 : L=151962.5822746655 DL=  1.04e-04 (AVERGE_DL)=    0.0000533789
ITERATION  89 : L=151962.5823744878 DL=  9.98e-05 (AVERGE_DL)=    0.0000522351
ITERATION  90 : L=151962.5824698465 DL=  9.54e-05 (AVERGE_DL)=    0.0000510539
ITERATION  91 : L=151962.5825607291 DL=  9.09e-05 (AVERGE_DL)=    0.0000498412
ITERATION  92 : L=151962.5826471530 DL=  8.64e-05 (AVERGE_DL)=    0.0000486033
ITERATION  93 : L=151962.5827291693 DL=  8.20e-05 (AVERGE_DL)=    0.0000473477
ITERATION  94 : L=151962.5828068412 DL=  7.77e-05 (AVERGE_DL)=    0.0000460766
ITERATION  95 : L=151962.5828802635 DL=  7.34e-05 (AVERGE_DL)=    0.0000447984
ITERATION  96 : L=151962.5829495414 DL=  6.93e-05 (AVERGE_DL)=    0.0000435157
ITERATION  97 : L=151962.5830148000 DL=  6.53e-05 (AVERGE_DL)=    0.0000422345
ITERATION  98 : L=151962.5830761768 DL=  6.14e-05 (AVERGE_DL)=    0.0000409591
ITERATION  99 : L=151962.5831338115 DL=  5.76e-05 (AVERGE_DL)=    0.0000396908
ITERATION 100 : L=151962.5831878583 DL=  5.40e-05 (AVERGE_DL)=    0.0000384356
ITERATION 101 : L=151962.5832384703 DL=  5.06e-05 (AVERGE_DL)=    0.0000371942
ITERATION 102 : L=151962.5832858127 DL=  4.73e-05 (AVERGE_DL)=    0.0000359727
ITERATION 103 : L=151962.5833300411 DL=  4.42e-05 (AVERGE_DL)=    0.0000347696
ITERATION 104 : L=151962.5833713184 DL=  4.13e-05 (AVERGE_DL)=    0.0000335895
ITERATION 105 : L=151962.5834098013 DL=  3.85e-05 (AVERGE_DL)=    0.0000324327
ITERATION 106 : L=151962.5834456471 DL=  3.58e-05 (AVERGE_DL)=    0.0000313017
ITERATION 107 : L=151962.5834790078 DL=  3.34e-05 (AVERGE_DL)=    0.0000301972
ITERATION 108 : L=151962.5835100304 DL=  3.10e-05 (AVERGE_DL)=    0.0000291198
ITERATION 109 : L=151962.5835388538 DL=  2.88e-05 (AVERGE_DL)=    0.0000280686
ITERATION 110 : L=151962.5835656162 DL=  2.68e-05 (AVERGE_DL)=    0.0000270465
ITERATION 111 : L=151962.5835904480 DL=  2.48e-05 (AVERGE_DL)=    0.0000260527
ITERATION 112 : L=151962.5836134804 DL=  2.30e-05 (AVERGE_DL)=    0.0000250910
ITERATION 113 : L=151962.5836348251 DL=  2.13e-05 (AVERGE_DL)=    0.0000241542
ITERATION 114 : L=151962.5836545990 DL=  1.98e-05 (AVERGE_DL)=    0.0000232485
ITERATION 115 : L=151962.5836729088 DL=  1.83e-05 (AVERGE_DL)=    0.0000223713
ITERATION 116 : L=151962.5836898505 DL=  1.69e-05 (AVERGE_DL)=    0.0000215192
ITERATION 117 : L=151962.5837055287 DL=  1.57e-05 (AVERGE_DL)=    0.0000207012
ITERATION 118 : L=151962.5837200264 DL=  1.45e-05 (AVERGE_DL)=    0.0000199067
ITERATION 119 : L=151962.5837334275 DL=  1.34e-05 (AVERGE_DL)=    0.0000191390
```

```
ITERATION 120 : L=151962.5837458155 DL=  1.24e-05 (AVERGE_DL)=    0.0000184013
ITERATION 121 : L=151962.5837572597 DL=  1.14e-05 (AVERGE_DL)=    0.0000176865
ITERATION 122 : L=151962.5837678284 DL=  1.06e-05 (AVERGE_DL)=    0.0000169964
ITERATION 123 : L=151962.5837775905 DL=  9.76e-06 (AVERGE_DL)=    0.0000163350
ITERATION 124 : L=151962.5837866015 DL=  9.01e-06 (AVERGE_DL)=    0.0000156941
ITERATION 125 : L=151962.5837949172 DL=  8.32e-06 (AVERGE_DL)=    0.0000150764
ITERATION 126 : L=151962.5838025917 DL=  7.67e-06 (AVERGE_DL)=    0.0000144834
ITERATION 127 : L=151962.5838096727 DL=  7.08e-06 (AVERGE_DL)=    0.0000139123
ITERATION 128 : L=151962.5838162059 DL=  6.53e-06 (AVERGE_DL)=    0.0000133632
ITERATION 129 : L=151962.5838222319 DL=  6.03e-06 (AVERGE_DL)=    0.0000128340
ITERATION 130 : L=151962.5838277908 DL=  5.56e-06 (AVERGE_DL)=    0.0000123266
ITERATION 131 : L=151962.5838329147 DL=  5.12e-06 (AVERGE_DL)=    0.0000118344
ITERATION 132 : L=151962.5838376393 DL=  4.72e-06 (AVERGE_DL)=    0.0000113641
ITERATION 133 : L=151962.5838419932 DL=  4.35e-06 (AVERGE_DL)=    0.0000109090
ITERATION 134 : L=151962.5838460083 DL=  4.02e-06 (AVERGE_DL)=    0.0000104762
ITERATION 135 : L=151962.5838497097 DL=  3.70e-06 (AVERGE_DL)=    0.0000100585
ITERATION 136 : L=151962.5838531199 DL=  3.41e-06 (AVERGE_DL)=    0.0000096546
ITERATION 137 : L=151962.5838562630 DL=  3.14e-06 (AVERGE_DL)=    0.0000092689
ITERATION 138 : L=151962.5838591615 DL=  2.90e-06 (AVERGE_DL)=    0.0000089009
ITERATION 139 : L=151962.5838618303 DL=  2.67e-06 (AVERGE_DL)=    0.0000085409
ITERATION 140 : L=151962.5838642906 DL=  2.46e-06 (AVERGE_DL)=    0.0000082006
ITERATION 141 : L=151962.5838665564 DL=  2.27e-06 (AVERGE_DL)=    0.0000078698
ITERATION 142 : L=151962.5838686448 DL=  2.09e-06 (AVERGE_DL)=    0.0000075553
ITERATION 143 : L=151962.5838705673 DL=  1.92e-06 (AVERGE_DL)=    0.0000072490
ITERATION 144 : L=151962.5838723391 DL=  1.77e-06 (AVERGE_DL)=    0.0000069592
ITERATION 145 : L=151962.5838739712 DL=  1.63e-06 (AVERGE_DL)=    0.0000066793
ITERATION 146 : L=151962.5838754751 DL=  1.50e-06 (AVERGE_DL)=    0.0000064113
ITERATION 147 : L=151962.5838768606 DL=  1.39e-06 (AVERGE_DL)=    0.0000061540
ITERATION 148 : L=151962.5838781356 DL=  1.27e-06 (AVERGE_DL)=    0.0000059034
ITERATION 149 : L=151962.5838793115 DL=  1.18e-06 (AVERGE_DL)=    0.0000056693
ITERATION 150 : L=151962.5838803930 DL=  1.08e-06 (AVERGE_DL)=    0.0000054370
ITERATION 151 : L=151962.5838813898 DL=  9.97e-07 (AVERGE_DL)=    0.0000052197
LOCALIZATION SUM CONVERGED


-------------------------------------------------------------------------------
                    LOCALIZED MOLECULAR ORBITAL COMPOSITIONS
-------------------------------------------------------------------------------

The Mulliken populations for each LMO on each atom are computed
The LMO`s will be ordered according to atom index and type
  (A) Strongly localized MO`s have populations of >=0.950 on one atom
  (B) Two center bond orbitals have populations of >=0.850 on two atoms
  (C) Other MO`s are considered to be `delocalized`

FOUND  -  37 strongly local MO`s
       - 234 two center bond MO`s
       -   0 significantly delocalized MO`s

Rather strongly localized orbitals:
MO 167:  11O  -   0.956048
MO 166:   8F  -   0.954359
MO 165:   8F  -   0.956314
MO 164:   8F  -   0.956090
MO 163:   7F  -   0.959190
MO 162:   7F  -   0.957072
MO 161:   7F  -   0.954005
MO 160:   6F  -   0.953789
MO 159:   6F  -   0.959796
MO 158:   6F  -   0.957451
MO 157:   4P  -   0.999557
MO 156:   4P  -   0.999872
MO 155:   4P  -   1.000005
MO 154:   3P  -   1.000041
MO 153:   3P  -   0.999330
MO 152:   3P  -   0.999756
MO 151:   2P  -   0.999302
MO 150:   2P  -   0.999767
MO 149:   2P  -   1.000085
MO 148:   1La -   1.000377
MO 147:   1La -   1.001167
MO 146:   1La -   1.001205
MO 145:   1La -   1.000166
MO 144:   1La -   1.000966
MO 143:   1La -   0.999479
MO 142:   1La -   0.999126
MO 141:   1La -   1.000924
MO 140:   1La -   1.022381
MO 139:   0Ag -   0.996441
MO 138:   0Ag -   0.988764
MO 137:   0Ag -   0.992613
MO 136:   0Ag -   0.992954
```

```
MO 135:   0Ag -   0.991358
MO 134:   0Ag -   0.989355
MO 133:   0Ag -   0.995518
MO 132:   0Ag -   0.995628
MO 131:   0Ag -   0.987089
Bond-like localized orbitals:
MO 401: 183H  -   0.496248  and  37C  -   0.530661
MO 400: 182H  -   0.547104  and 175C  -   0.439460
MO 399: 181H  -   0.492270  and 178C  -   0.504491
MO 398: 180H  -   0.481590  and 178C  -   0.521494
MO 397: 179H  -   0.490412  and 178C  -   0.513478
MO 396: 178C  -   0.507424  and  79C  -   0.533979
MO 395: 177H  -   0.500910  and 175C  -   0.485961
MO 394: 176H  -   0.490200  and 175C  -   0.504258
MO 393: 175C  -   0.515532  and  79C  -   0.529837
MO 392: 174H  -   0.491123  and 171C  -   0.509878
MO 391: 173H  -   0.487452  and 171C  -   0.512957
MO 390: 172H  -   0.486080  and 171C  -   0.515448
MO 389: 171C  -   0.496297  and  79C  -   0.541155
MO 388: 170H  -   0.488390  and 167C  -   0.508458
MO 387: 169H  -   0.490200  and 167C  -   0.503159
MO 386: 168H  -   0.486537  and 167C  -   0.509549
MO 385: 167C  -   0.508665  and 117C  -   0.530702
MO 384: 166H  -   0.476163  and 163C  -   0.514524
MO 383: 165H  -   0.457267  and 163C  -   0.527022
MO 382: 164H  -   0.487949  and 163C  -   0.502405
MO 381: 163C  -   0.477021  and 117C  -   0.554322
MO 380: 162H  -   0.450767  and 159C  -   0.525547
MO 379: 161H  -   0.469562  and 159C  -   0.513660
MO 378: 160H  -   0.485230  and 159C  -   0.495471
MO 377: 159C  -   0.469965  and 124C  -   0.557923
MO 376: 158H  -   0.491397  and 155C  -   0.509224
MO 375: 157H  -   0.483790  and 155C  -   0.520056
MO 374: 156H  -   0.487791  and 155C  -   0.513788
MO 373: 155C  -   0.501986  and 133C  -   0.539779
MO 372: 154H  -   0.488944  and 151C  -   0.506194
MO 371: 153H  -   0.484012  and 151C  -   0.512243
MO 370: 152H  -   0.492007  and 151C  -   0.503886
MO 369: 151C  -   0.503040  and 133C  -   0.541876
MO 368: 150H  -   0.510064  and 149C  -   0.516763
MO 367: 149C  -   0.514489  and  95C  -   0.503676
MO 366: 149C  -   0.455785  and  91C  -   0.467579
MO 365: 149C  -   0.457605  and  91C  -   0.464933
MO 364: 148H  -   0.487078  and 145C  -   0.509265
MO 363: 147H  -   0.485828  and 145C  -   0.510184
MO 362: 146H  -   0.492300  and 145C  -   0.504870
MO 361: 145C  -   0.504985  and 108C  -   0.541422
MO 360: 144H  -   0.511514  and 143C  -   0.513875
MO 359: 143C  -   0.476823  and 139C  -   0.452653
MO 358: 143C  -   0.457018  and 139C  -   0.470673
MO 357: 143C  -   0.522249  and 106C  -   0.491500
MO 356: 142H  -   0.511719  and 141C  -   0.514855
MO 355: 141C  -   0.504155  and 139C  -   0.514736
MO 354: 141C  -   0.467313  and 122C  -   0.453144
MO 353: 141C  -   0.466198  and 122C  -   0.457865
MO 352: 140H  -   0.511977  and 139C  -   0.516775
MO 351: 138C  -   0.344612  and   8F  -   0.672004
MO 350: 138C  -   0.336118  and   7F  -   0.668760
MO 349: 138C  -   0.336936  and   6F  -   0.668793
MO 348: 138C  -   0.513429  and   5S  -   0.568023
MO 347: 137H  -   0.490133  and 134C  -   0.511749
MO 346: 136H  -   0.490585  and 134C  -   0.509361
MO 345: 135H  -   0.486190  and 134C  -   0.514299
MO 344: 134C  -   0.504170  and 133C  -   0.534766
MO 343: 133C  -   0.538409  and  85C  -   0.499082
MO 342: 132H  -   0.490807  and 129C  -   0.504094
MO 341: 131H  -   0.486738  and 129C  -   0.508753
MO 340: 130H  -   0.489934  and 129C  -   0.504910
MO 339: 129C  -   0.510027  and 124C  -   0.528291
MO 338: 128H  -   0.490396  and 125C  -   0.501240
MO 337: 127H  -   0.468562  and 125C  -   0.524825
MO 336: 126H  -   0.444242  and 125C  -   0.541539
MO 335: 125C  -   0.468822  and 124C  -   0.561053
MO 334: 124C  -   0.521965  and  86C  -   0.511520
MO 333: 123H  -   0.517024  and 122C  -   0.505749
MO 332: 122C  -   0.491007  and 105C  -   0.516015
MO 331: 121H  -   0.486614  and 118C  -   0.496691
MO 330: 120H  -   0.449733  and 118C  -   0.525814
MO 329: 119H  -   0.464550  and 118C  -   0.519602
MO 328: 118C  -   0.466463  and 117C  -   0.559139
MO 327: 117C  -   0.522238  and 104C  -   0.512996
MO 326: 116H  -   0.491346  and 113C  -   0.506442
```

MO 325:	115H	-	0.483368	and	113C	-	0.518573
MO 324:	114H	-	0.487187	and	113C	-	0.515569
MO 323:	113C	-	0.504341	and	108C	-	0.532603
MO 322:	112H	-	0.485042	and	109C	-	0.518062
MO 321:	111H	-	0.491427	and	109C	-	0.509202
MO 320:	110H	-	0.488637	and	109C	-	0.513174
MO 319:	109C	-	0.501802	and	108C	-	0.539566
MO 318:	108C	-	0.539462	and	103C	-	0.498376
MO 317:	107H	-	0.502331	and	106C	-	0.528063
MO 316:	106C	-	0.448405	and	105C	-	0.472282
MO 315:	106C	-	0.458496	and	105C	-	0.466171
MO 314:	105C	-	0.543957	and	2P	-	0.453051
MO 313:	104C	-	0.448392	and	101C	-	0.483642
MO 312:	104C	-	0.458781	and	101C	-	0.475372
MO 311:	104C	-	0.519621	and	97C	-	0.508338
MO 310:	103C	-	0.517745	and	101C	-	0.503941
MO 309:	103C	-	0.465805	and	99C	-	0.478273
MO 308:	103C	-	0.471664	and	99C	-	0.466645
MO 307:	102H	-	0.528247	and	101C	-	0.505735
MO 306:	100H	-	0.514931	and	99C	-	0.516259
MO 305:	99C	-	0.486346	and	98C	-	0.506289
MO 304:	98C	-	0.550272	and	97C	-	0.376453
MO 303:	98C	-	0.536971	and	97C	-	0.380428
MO 302:	98C	-	0.502410	and	2P	-	0.491318
MO 301:	97C	-	0.367960	and	14O	-	0.623000
MO 300:	96H	-	0.510182	and	95C	-	0.518145
MO 299:	95C	-	0.472825	and	93C	-	0.449017
MO 298:	95C	-	0.470928	and	93C	-	0.449844
MO 297:	94H	-	0.502200	and	93C	-	0.526336
MO 296:	93C	-	0.506538	and	88C	-	0.502925
MO 295:	92H	-	0.512253	and	91C	-	0.514569
MO 294:	91C	-	0.519066	and	89C	-	0.492239
MO 293:	90H	-	0.514367	and	89C	-	0.512273
MO 292:	89C	-	0.438510	and	88C	-	0.481984
MO 291:	89C	-	0.445754	and	88C	-	0.473418
MO 290:	88C	-	0.545240	and	3P	-	0.459994
MO 289:	87C	-	0.524355	and	86C	-	0.505160
MO 288:	87C	-	0.386084	and	80C	-	0.530373
MO 287:	87C	-	0.360667	and	80C	-	0.555220
MO 286:	87C	-	0.369810	and	13O	-	0.621204
MO 285:	86C	-	0.455171	and	83C	-	0.464558
MO 284:	86C	-	0.467041	and	83C	-	0.458532
MO 283:	85C	-	0.526575	and	83C	-	0.497631
MO 282:	85C	-	0.469612	and	81C	-	0.458894
MO 281:	85C	-	0.465940	and	81C	-	0.467343
MO 280:	84H	-	0.525080	and	83C	-	0.509319
MO 279:	82H	-	0.519826	and	81C	-	0.511181
MO 278:	81C	-	0.504718	and	80C	-	0.487537
MO 277:	80C	-	0.506999	and	4P	-	0.492302
MO 276:	79C	-	0.536024	and	74C	-	0.502176
MO 275:	78C	-	0.381898	and	77C	-	0.542515
MO 274:	78C	-	0.369574	and	77C	-	0.551985
MO 273:	78C	-	0.513258	and	71C	-	0.517952
MO 272:	78C	-	0.371175	and	12O	-	0.620896
MO 271:	77C	-	0.507740	and	75C	-	0.484647
MO 270:	77C	-	0.504520	and	3P	-	0.489516
MO 269:	76H	-	0.536749	and	75C	-	0.494643
MO 268:	75C	-	0.471201	and	74C	-	0.465544
MO 267:	75C	-	0.467803	and	74C	-	0.470641
MO 266:	74C	-	0.516302	and	72C	-	0.505769
MO 265:	73H	-	0.526973	and	72C	-	0.507528
MO 264:	72C	-	0.470943	and	71C	-	0.465207
MO 263:	72C	-	0.472865	and	71C	-	0.447633
MO 262:	71C	-	0.509473	and	58C	-	0.526886
MO 261:	70H	-	0.491148	and	67C	-	0.504504
MO 260:	69H	-	0.488493	and	67C	-	0.507784
MO 259:	68H	-	0.488097	and	67C	-	0.505766
MO 258:	67C	-	0.509780	and	58C	-	0.531797
MO 257:	66H	-	0.448550	and	63C	-	0.533256
MO 256:	65H	-	0.477892	and	63C	-	0.507704
MO 255:	64H	-	0.476628	and	63C	-	0.505581
MO 254:	63C	-	0.477187	and	58C	-	0.549910
MO 253:	62H	-	0.488162	and	59C	-	0.502108
MO 252:	61H	-	0.482664	and	59C	-	0.509445
MO 251:	60H	-	0.430570	and	59C	-	0.554291
MO 250:	59C	-	0.467440	and	58C	-	0.561368
MO 249:	57H	-	0.510408	and	56C	-	0.516605
MO 248:	56C	-	0.463506	and	54C	-	0.460925
MO 247:	56C	-	0.460387	and	54C	-	0.465516
MO 246:	56C	-	0.510196	and	52C	-	0.508136
MO 245:	55H	-	0.513565	and	54C	-	0.515399
MO 244:	54C	-	0.523891	and	50C	-	0.488481

MO 243:	53H	-	0.510255	and	52C	-	0.515793
MO 242:	52C	-	0.472458	and	48C	-	0.449044
MO 241:	52C	-	0.471776	and	48C	-	0.455629
MO 240:	51H	-	0.520600	and	50C	-	0.513592
MO 239:	50C	-	0.427255	and	47C	-	0.504151
MO 238:	50C	-	0.446470	and	47C	-	0.478229
MO 237:	49H	-	0.509680	and	48C	-	0.519148
MO 236:	48C	-	0.507302	and	47C	-	0.497090
MO 235:	47C	-	0.529622	and	2P	-	0.472405
MO 234:	46H	-	0.508531	and	45C	-	0.516305
MO 233:	45C	-	0.504906	and	43C	-	0.513959
MO 232:	45C	-	0.481306	and	37C	-	0.444200
MO 231:	45C	-	0.475537	and	37C	-	0.446156
MO 230:	44H	-	0.511254	and	43C	-	0.515805
MO 229:	43C	-	0.455636	and	41C	-	0.473510
MO 228:	43C	-	0.463423	and	41C	-	0.458256
MO 227:	42H	-	0.517442	and	41C	-	0.509011
MO 226:	41C	-	0.518552	and	39C	-	0.492504
MO 225:	40H	-	0.505053	and	39C	-	0.523848
MO 224:	39C	-	0.435812	and	38C	-	0.489116
MO 223:	39C	-	0.447430	and	38C	-	0.481080
MO 222:	38C	-	0.511907	and	37C	-	0.496498
MO 221:	38C	-	0.528051	and	3P	-	0.478630
MO 220:	36H	-	0.509974	and	35C	-	0.517315
MO 219:	35C	-	0.516697	and	33C	-	0.503029
MO 218:	35C	-	0.462352	and	29C	-	0.468683
MO 217:	35C	-	0.463075	and	29C	-	0.470239
MO 216:	34H	-	0.510767	and	33C	-	0.514941
MO 215:	33C	-	0.471757	and	27C	-	0.460185
MO 214:	33C	-	0.473472	and	27C	-	0.445718
MO 213:	32H	-	0.498488	and	31C	-	0.528236
MO 212:	31C	-	0.494015	and	29C	-	0.521261
MO 211:	31C	-	0.444792	and	26C	-	0.484008
MO 210:	31C	-	0.459926	and	26C	-	0.464559
MO 209:	30H	-	0.509570	and	29C	-	0.516635
MO 208:	28H	-	0.506294	and	27C	-	0.512469
MO 207:	27C	-	0.494564	and	26C	-	0.515287
MO 206:	26C	-	0.551632	and	4P	-	0.452499
MO 205:	25H	-	0.493994	and	24C	-	0.528145
MO 204:	24C	-	0.493272	and	22C	-	0.520035
MO 203:	24C	-	0.440158	and	15C	-	0.481482
MO 202:	24C	-	0.432051	and	15C	-	0.489622
MO 201:	23H	-	0.507116	and	22C	-	0.516642
MO 200:	22C	-	0.458900	and	20C	-	0.468244
MO 199:	22C	-	0.476062	and	20C	-	0.449917
MO 198:	21H	-	0.510089	and	20C	-	0.517489
MO 197:	20C	-	0.512815	and	18C	-	0.505461
MO 196:	19H	-	0.515562	and	18C	-	0.512227
MO 195:	18C	-	0.472593	and	16C	-	0.451843
MO 194:	18C	-	0.466563	and	16C	-	0.449481
MO 193:	17H	-	0.516284	and	16C	-	0.513987
MO 192:	16C	-	0.497588	and	15C	-	0.514021
MO 191:	15C	-	0.529244	and	4P	-	0.468455
**MO 190:**	**14O**	**-**	**0.895880**	**and**	**1La**	**-**	**0.080339**
**MO 189:**	**14O**	**-**	**0.903299**	**and**	**1La**	**-**	**0.071614**
**MO 188:**	**14O**	**-**	**0.841622**	**and**	**0Ag**	**-**	**0.051141**
**MO 187:**	**13O**	**-**	**0.898358**	**and**	**1La**	**-**	**0.101116**
**MO 186:**	**13O**	**-**	**0.900844**	**and**	**1La**	**-**	**0.077049**
**MO 185:**	**13O**	**-**	**0.847470**	**and**	**0Ag**	**-**	**0.065021**
**MO 184:**	**12O**	**-**	**0.899085**	**and**	**1La**	**-**	**0.075734**
**MO 183:**	**12O**	**-**	**0.900603**	**and**	**1La**	**-**	**0.095129**
**MO 182:**	**12O**	**-**	**0.843709**	**and**	**0Ag**	**-**	**0.061461**
MO 181:	11O	-	0.719544	and	5S	-	0.274568
MO 180:	11O	-	0.753467	and	5S	-	0.243658
MO 179:	11O	-	0.747244	and	5S	-	0.248865
MO 178:	10O	-	0.834492	and	5S	-	0.131635
MO 177:	10O	-	0.590981	and	5S	-	0.403792
MO 176:	10O	-	0.884162	and	5S	-	0.063651
**MO 175:**	**10O**	**-**	**0.917864**	**and**	**1La**	**-**	**0.050837**
MO 174:	9O	-	0.849906	and	5S	-	0.115901
MO 173:	9O	-	0.885148	and	5S	-	0.062187
MO 172:	9O	-	0.583780	and	5S	-	0.410658
**MO 171:**	**9O**	**-**	**0.918486**	**and**	**1La**	**-**	**0.046784**
**MO 170:**	**4P**	**-**	**0.881404**	**and**	**0Ag**	**-**	**0.123284**
**MO 169:**	**3P**	**-**	**0.869611**	**and**	**0Ag**	**-**	**0.147803**
**MO 168:**	**2P**	**-**	**0.879976**	**and**	**0Ag**	**-**	**0.125032**

Pipek-Mezey analysis for 3-Y

```
------------------------------------------------------------------------------
                           ORCA ORBITAL LOCALIZATION
------------------------------------------------------------------------------

Input orbitals are from                  ... .\Y_Ag.gbw
Output orbitals are to                   ... .\Y_Ag.loc
Max. number of iterations                ... 1000
Localizations seeded randomly            ... on
Convergence tolerance                    ... 1.000e-06
Treshold for strong local MOs            ... 9.500e-01
Treshold for bond MOs                    ... 8.500e-01
Operator                                 ... 0
Orbital range for localization           ... 127 to 392
Localization criterion                   ... PIPEK-MEZEY
Entering Jacobi type localization:
Using Cholesky decomposition as initial guess.
Initial value of the localization sum :   107.908842
ITERATION   0 : L=  153.2820619761 DL=  4.54e+01 (MAX-T)=  44.733
ITERATION   1 : L=  154.4721407053 DL=  1.19e+00 (MAX-T)=  32.699
ITERATION   2 : L=  154.5017171605 DL=  2.96e-02 (MAX-T)=   8.304
ITERATION   3 : L=  154.5183659184 DL=  1.66e-02 (MAX-T)=   3.917
ITERATION   4 : L=  154.5245687045 DL=  6.20e-03 (MAX-T)=   3.534
ITERATION   5 : L=  154.5299956200 DL=  5.43e-03 (MAX-T)=   2.310
ITERATION   6 : L=  154.5323207756 DL=  2.33e-03 (MAX-T)=   1.171
ITERATION   7 : L=  154.5333746783 DL=  1.05e-03 (MAX-T)=   1.301
ITERATION   8 : L=  154.5344121941 DL=  1.04e-03 (MAX-T)=   1.338
ITERATION   9 : L=  154.5350450582 DL=  6.33e-04 (MAX-T)=   1.368
ITERATION  10 : L=  154.5359407261 DL=  8.96e-04 (MAX-T)=   1.271
ITERATION  11 : L=  154.5367436009 DL=  8.03e-04 (MAX-T)=   1.241
ITERATION  12 : L=  154.5377063278 DL=  9.63e-04 (MAX-T)=   1.229
ITERATION  13 : L=  154.5383833678 DL=  6.77e-04 (MAX-T)=   1.244
ITERATION  14 : L=  154.5389055632 DL=  5.22e-04 (MAX-T)=   1.156
ITERATION  15 : L=  154.5392382184 DL=  3.33e-04 (MAX-T)=   0.962
ITERATION  16 : L=  154.5398148895 DL=  5.77e-04 (MAX-T)=   0.959
ITERATION  17 : L=  154.5401618272 DL=  3.47e-04 (MAX-T)=   0.853
ITERATION  18 : L=  154.5404944018 DL=  3.33e-04 (MAX-T)=   0.769
ITERATION  19 : L=  154.5407115795 DL=  2.17e-04 (MAX-T)=   0.635
ITERATION  20 : L=  154.5408399989 DL=  1.28e-04 (MAX-T)=   0.679
ITERATION  21 : L=  154.5409964415 DL=  1.56e-04 (MAX-T)=   0.580
ITERATION  22 : L=  154.5411447065 DL=  1.48e-04 (MAX-T)=   0.514
ITERATION  23 : L=  154.5412486023 DL=  1.04e-04 (MAX-T)=   0.434
ITERATION  24 : L=  154.5412938725 DL=  4.53e-05 (MAX-T)=   0.401
ITERATION  25 : L=  154.5413594739 DL=  6.56e-05 (MAX-T)=   0.315
ITERATION  26 : L=  154.5413968741 DL=  3.74e-05 (MAX-T)=   0.302
ITERATION  27 : L=  154.5414302056 DL=  3.33e-05 (MAX-T)=   0.294
ITERATION  28 : L=  154.5414511442 DL=  2.09e-05 (MAX-T)=   0.224
ITERATION  29 : L=  154.5414722964 DL=  2.12e-05 (MAX-T)=   0.195
ITERATION  30 : L=  154.5414861408 DL=  1.38e-05 (MAX-T)=   0.172
ITERATION  31 : L=  154.5415028653 DL=  1.67e-05 (MAX-T)=   0.169
ITERATION  32 : L=  154.5415120799 DL=  9.21e-06 (MAX-T)=   0.128
ITERATION  33 : L=  154.5415173876 DL=  5.31e-06 (MAX-T)=   0.134
ITERATION  34 : L=  154.5415233396 DL=  5.95e-06 (MAX-T)=   0.115
ITERATION  35 : L=  154.5415280395 DL=  4.70e-06 (MAX-T)=   0.098
ITERATION  36 : L=  154.5415312628 DL=  3.22e-06 (MAX-T)=   0.085
ITERATION  37 : L=  154.5415345823 DL=  3.32e-06 (MAX-T)=   0.067
ITERATION  38 : L=  154.5415360008 DL=  1.42e-06 (MAX-T)=   0.054
ITERATION  39 : L=  154.5415377063 DL=  1.71e-06 (MAX-T)=   0.064
ITERATION  40 : L=  154.5415392843 DL=  1.58e-06 (MAX-T)=   0.051
ITERATION  41 : L=  154.5415401058 DL=  8.22e-07 (MAX-T)=   0.043
LOCALIZATION SUM CONVERGED


--------------------------------------------------------------------------------
                    LOCALIZED MOLECULAR ORBITAL COMPOSITIONS
--------------------------------------------------------------------------------

The Mulliken populations for each LMO on each atom are computed
The LMO`s will be ordered according to atom index and type
  (A) Strongly localized MO`s have populations of >=0.950 on one atom
  (B) Two center bond orbitals have populations of >=0.850 on two atoms
  (C) Other MO`s are considered to be `delocalized`

FOUND  -  28 strongly local MO`s
       - 211 two center bond MO`s
       -  27 significantly delocalized MO`s

Rather strongly localized orbitals:
MO 154:  74F  -   1.014112
MO 153:  46F  -   1.013932
MO 152:  39O  -   1.014682
```

```
MO 151:  27F  -   1.011253
MO 150:  26O  -   1.021476
MO 149:  24O  -   0.970211
MO 148:  16P  -   0.999857
MO 147:  16P  -   1.000175
MO 146:  16P  -   1.002094
MO 145:  15P  -   0.999970
MO 144:  15P  -   1.000099
MO 143:  15P  -   1.003175
MO 142:  14P  -   1.001911
MO 141:  14P  -   0.999884
MO 140:  14P  -   1.000225
MO 139:  13Y  -   1.006922
MO 138:  13Y  -   1.002899
MO 137:  13Y  -   1.006993
MO 136:  13Y  -   1.024623
MO 135:  12Ag -   0.995442
MO 134:  12Ag -   0.990631
MO 133:  12Ag -   0.977165
MO 132:  12Ag -   0.977915
MO 131:  12Ag -   0.991037
MO 130:  12Ag -   1.000401
MO 129:  12Ag -   1.004119
MO 128:  12Ag -   1.003724
MO 127:  12Ag -   1.008845
Bond-like localized orbitals:
MO 365: 183H  -   0.486320  and 180C  -   0.515079
MO 364: 182H  -   0.489987  and 180C  -   0.510496
MO 363: 181H  -   0.490462  and 180C  -   0.512443
MO 362: 180C  -   0.507064  and  75C  -   0.533896
MO 361: 179H  -   0.493319  and 176C  -   0.503539
MO 360: 178H  -   0.459688  and 176C  -   0.536393
MO 359: 177H  -   0.467003  and 176C  -   0.529218
MO 358: 176C  -   0.469409  and 163C  -   0.563924
MO 357: 175H  -   0.469249  and 172C  -   0.522190
MO 356: 174H  -   0.471165  and 172C  -   0.517540
MO 355: 173H  -   0.487999  and 172C  -   0.499230
MO 354: 172C  -   0.476501  and 163C  -   0.556791
MO 353: 171H  -   0.487016  and 168C  -   0.508842
MO 352: 170H  -   0.490808  and 168C  -   0.504861
MO 351: 169H  -   0.488813  and 168C  -   0.506476
MO 350: 168C  -   0.509086  and 163C  -   0.533843
MO 349: 167H  -   0.482119  and 164C  -   0.509738
MO 348: 166H  -   0.479965  and 164C  -   0.509220
MO 347: 165H  -   0.464683  and 164C  -   0.526964
MO 346: 164C  -   0.476941  and  99C  -   0.555085
MO 345: 163C  -   0.521513  and  51C  -   0.516142
MO 344: 162H  -   0.488142  and 159C  -   0.507472
MO 343: 161H  -   0.491512  and 159C  -   0.505305
MO 342: 160H  -   0.483857  and 159C  -   0.513143
MO 341: 159C  -   0.500313  and  75C  -   0.547266
MO 340: 158H  -   0.486185  and 155C  -   0.510689
MO 339: 157H  -   0.490914  and 155C  -   0.506669
MO 338: 156H  -   0.485543  and 155C  -   0.510092
MO 337: 155C  -   0.501353  and  59C  -   0.546535
MO 336: 154H  -   0.512068  and 153C  -   0.526474
MO 335: 153C  -   0.516149  and 141C  -   0.504336
MO 334: 153C  -   0.513313  and  80C  -   0.507662
MO 333: 152H  -   0.503582  and 151C  -   0.542156
MO 332: 151C  -   0.488536  and 115C  -   0.526802
MO 331: 151C  -   0.494371  and  49C  -   0.520883
MO 330: 150H  -   0.484947  and 147C  -   0.519407
MO 329: 149H  -   0.487933  and 147C  -   0.514343
MO 328: 148H  -   0.490756  and 147C  -   0.510659
MO 327: 147C  -   0.502428  and  59C  -   0.541621
MO 326: 146H  -   0.490760  and 143C  -   0.507153
MO 325: 145H  -   0.484812  and 143C  -   0.518115
MO 324: 144H  -   0.484023  and 143C  -   0.519198
MO 323: 143C  -   0.505735  and  59C  -   0.532814
MO 322: 142H  -   0.517358  and 141C  -   0.518040
MO 321: 141C  -   0.521543  and 129C  -   0.491232
MO 320: 140C  -   0.333307  and  74F  -   0.671928
MO 319: 140C  -   0.327052  and  46F  -   0.675644
MO 318: 140C  -   0.322480  and  27F  -   0.677565
MO 317: 140C  -   0.485781  and  17S  -   0.607562
MO 316: 139H  -   0.512207  and 138C  -   0.524661
MO 315: 138C  -   0.504441  and 125C  -   0.516920
MO 314: 138C  -   0.518433  and  62C  -   0.495975
MO 313: 137H  -   0.511259  and 136C  -   0.528044
MO 312: 136C  -   0.510794  and 103C  -   0.509634
MO 311: 136C  -   0.520327  and  64C  -   0.499960
MO 310: 135H  -   0.512113  and 134C  -   0.528304
```

MO 309:	134C	-	0.515136	and	131C	-	0.505198
MO 308:	134C	-	0.511933	and	115C	-	0.509287
MO 307:	133C	-	0.514169	and	87C	-	0.501665
MO 306:	133C	-	0.521453	and	62C	-	0.494262
MO 305:	133C	-	0.562744	and	15P	-	0.445187
MO 304:	132H	-	0.512509	and	131C	-	0.524959
MO 303:	131C	-	0.517255	and	127C	-	0.498313
MO 302:	130H	-	0.509281	and	129C	-	0.540070
MO 301:	129C	-	0.492233	and	50C	-	0.525861
MO 300:	128H	-	0.522924	and	127C	-	0.514195
MO 299:	127C	-	0.487335	and	49C	-	0.525324
MO 298:	126H	-	0.510041	and	125C	-	0.527422
MO 297:	125C	-	0.515283	and	57C	-	0.506732
MO 296:	124H	-	0.484629	and	121C	-	0.519678
MO 295:	123H	-	0.491209	and	121C	-	0.510116
MO 294:	122H	-	0.487878	and	121C	-	0.514143
MO 293:	121C	-	0.502652	and	75C	-	0.541302
MO 292:	120H	-	0.487567	and	117C	-	0.507628
MO 291:	119H	-	0.488567	and	117C	-	0.508302
MO 290:	118H	-	0.490587	and	117C	-	0.506253
MO 289:	117C	-	0.509451	and	99C	-	0.535956
MO 288:	116H	-	0.512795	and	115C	-	0.522839
MO 287:	114C	-	0.499318	and	75C	-	0.540260
MO 286:	114C	-	0.538407	and	69C	-	0.488427
MO 285:	114C	-	0.506687	and	52C	-	0.520186
MO 284:	113C	-	0.529286	and	72C	-	0.495241
MO 283:	113C	-	0.498292	and	59C	-	0.542524
MO 282:	113C	-	0.503165	and	35C	-	0.528036
MO 281:	112H	-	0.510433	and	111C	-	0.526011
MO 280:	111C	-	0.518710	and	109C	-	0.501601
MO 279:	111C	-	0.515935	and	89C	-	0.504800
MO 278:	110H	-	0.513448	and	109C	-	0.525328
MO 277:	109C	-	0.520359	and	44C	-	0.492734
MO 276:	108H	-	0.489374	and	105C	-	0.507411
MO 275:	107H	-	0.485202	and	105C	-	0.510842
MO 274:	106H	-	0.490357	and	105C	-	0.503378
MO 273:	105C	-	0.507673	and	100C	-	0.533433
MO 272:	104H	-	0.510735	and	103C	-	0.524208
MO 271:	103C	-	0.518923	and	91C	-	0.496848
MO 270:	102H	-	0.525711	and	101C	-	0.525203
MO 269:	101C	-	0.496141	and	97C	-	0.516311
MO 268:	101C	-	0.493471	and	21C	-	0.522116
MO 267:	100C	-	0.563694	and	93C	-	0.469504
MO 266:	100C	-	0.558501	and	83C	-	0.476220
MO 265:	100C	-	0.517810	and	71C	-	0.519308
MO 264:	99C	-	0.525374	and	68C	-	0.513511
MO 263:	99C	-	0.561036	and	40C	-	0.471687
MO 262:	98H	-	0.519474	and	97C	-	0.520278
MO 261:	97C	-	0.505076	and	47C	-	0.515970
MO 260:	96H	-	0.489055	and	93C	-	0.496716
MO 259:	95H	-	0.455041	and	93C	-	0.537606
MO 258:	94H	-	0.477052	and	93C	-	0.509938
MO 257:	92H	-	0.511747	and	91C	-	0.531934
MO 256:	91C	-	0.502585	and	28C	-	0.506732
MO 255:	90H	-	0.509616	and	89C	-	0.527534
MO 254:	89C	-	0.521226	and	66C	-	0.495399
MO 253:	88H	-	0.501400	and	87C	-	0.540346
MO 252:	87C	-	0.490018	and	57C	-	0.525901
MO 251:	86H	-	0.464191	and	83C	-	0.531046
MO 250:	85H	-	0.471506	and	83C	-	0.523292
MO 249:	84H	-	0.490853	and	83C	-	0.504917
MO 248:	82C	-	0.541063	and	31C	-	0.501136
MO 247:	82C	-	0.546635	and	8C	-	0.498413
MO 246:	82C	-	0.533338	and	4C	-	0.509250
MO 245:	82C	-	0.540238	and	0C	-	0.502438
MO 244:	81H	-	0.510408	and	80C	-	0.525083
MO 243:	80C	-	0.527433	and	37C	-	0.489495
MO 242:	79H	-	0.525155	and	78C	-	0.530860
MO 241:	78C	-	0.485461	and	64C	-	0.527511
MO 240:	78C	-	0.489686	and	28C	-	0.534749
MO 239:	77H	-	0.524105	and	76C	-	0.528767
MO 238:	76C	-	0.528553	and	31C	-	0.502918
MO 237:	76C	-	0.476929	and	29C	-	0.519046
MO 236:	74F	-	0.933179	and	13Y	-	0.004686
MO 235:	74F	-	0.933239	and	13Y	-	0.007984
MO 234:	73H	-	0.525411	and	72C	-	0.529704
MO 233:	72C	-	0.543803	and	71C	-	0.473398
MO 232:	71C	-	0.514475	and	34C	-	0.522770
MO 231:	70H	-	0.522656	and	69C	-	0.533319
MO 230:	69C	-	0.520589	and	51C	-	0.494815
MO 229:	68C	-	0.475988	and	55C	-	0.539999
MO 228:	68C	-	0.513441	and	30C	-	0.528116

MO 227:	67H	-	0.503861	and	66C	-	0.538383
MO 226:	66C	-	0.505624	and	54C	-	0.506941
MO 225:	65H	-	0.514868	and	64C	-	0.525103
MO 224:	63H	-	0.511865	and	62C	-	0.522792
MO 223:	61H	-	0.508871	and	60C	-	0.526017
MO 222:	60C	-	0.505952	and	47C	-	0.514865
MO 221:	60C	-	0.527515	and	32C	-	0.488392
MO 220:	58H	-	0.509702	and	57C	-	0.525992
MO 219:	56H	-	0.524185	and	55C	-	0.529021
MO 218:	55C	-	0.500988	and	31C	-	0.524124
MO 217:	54C	-	0.515519	and	44C	-	0.501296
MO 216:	54C	-	0.558316	and	16P	-	0.454897
MO 215:	53H	-	0.520388	and	52C	-	0.532133
MO 214:	52C	-	0.505580	and	23C	-	0.493408
MO 213:	51C	-	0.501346	and	22C	-	0.540898
MO 212:	50C	-	0.518590	and	37C	-	0.494177
MO 211:	50C	-	0.532642	and	16P	-	0.479842
MO 210:	49C	-	0.552557	and	14P	-	0.452589
MO 209:	48H	-	0.511466	and	47C	-	0.528211
MO 208:	46F	-	0.932793	and	42H	-	0.007242
MO 207:	46F	-	0.935611	and	13Y	-	0.004706
MO 206:	45H	-	0.515674	and	44C	-	0.524435
MO 205:	43H	-	0.452688	and	40C	-	0.543310
MO 204:	42H	-	0.479483	and	40C	-	0.517677
MO 203:	41H	-	0.491157	and	40C	-	0.504978
MO 202:	39O	-	0.536261	and	17S	-	0.466205
MO 201:	39O	-	0.836199	and	17S	-	0.170538
MO 200:	39O	-	0.825366	and	17S	-	0.143036
MO 199:	38H	-	0.498481	and	37C	-	0.543434
MO 198:	36H	-	0.514343	and	35C	-	0.536733
MO 197:	35C	-	0.480717	and	25C	-	0.515811
MO 196:	34C	-	0.471265	and	25C	-	0.557913
MO 195:	34C	-	0.381677	and	18O	-	0.603927
MO 194:	33H	-	0.497721	and	32C	-	0.540727
MO 193:	32C	-	0.487663	and	21C	-	0.525265
MO 192:	30C	-	0.465227	and	29C	-	0.569280
MO 191:	30C	-	0.382966	and	19O	-	0.603469
MO 190:	29C	-	0.513152	and	16P	-	0.492977
MO 189:	28C	-	0.534044	and	14P	-	0.475503
MO 188:	27F	-	0.936028	and	17S	-	0.001631
**MO 187:**	**27F**	**-**	**0.934396**	**and**	**13Y**	**-**	**0.000653**
MO 186:	26O	-	0.823001	and	17S	-	0.148813
MO 185:	26O	-	0.831575	and	17S	-	0.173869
MO 184:	26O	-	0.530844	and	17S	-	0.470991
MO 183:	25C	-	0.504907	and	14P	-	0.494909
MO 182:	24O	-	0.616353	and	17S	-	0.364872
MO 181:	24O	-	0.873473	and	17S	-	0.083093
MO 180:	24O	-	0.859511	and	17S	-	0.082285
MO 179:	23C	-	0.571173	and	22C	-	0.456336
MO 178:	23C	-	0.513139	and	15P	-	0.492737
MO 177:	22C	-	0.381061	and	20O	-	0.605169
MO 176:	21C	-	0.535498	and	15P	-	0.469155
**MO 175:**	**20O**	**-**	**0.878234**	**and**	**13Y**	**-**	**0.065070**
**MO 174:**	**20O**	**-**	**0.943656**	**and**	**13Y**	**-**	**0.094645**
**MO 173:**	**20O**	**-**	**0.838911**	**and**	**12Ag**	**-**	**0.066456**
**MO 172:**	**19O**	**-**	**0.879826**	**and**	**13Y**	**-**	**0.068921**
**MO 171:**	**19O**	**-**	**0.942051**	**and**	**13Y**	**-**	**0.090924**
**MO 170:**	**19O**	**-**	**0.839154**	**and**	**12Ag**	**-**	**0.049163**
**MO 169:**	**18O**	**-**	**0.945124**	**and**	**13Y**	**-**	**0.081467**
**MO 168:**	**18O**	**-**	**0.878975**	**and**	**13Y**	**-**	**0.065650**
**MO 167:**	**18O**	**-**	**0.838769**	**and**	**12Ag**	**-**	**0.051072**
**MO 166:**	**16P**	**-**	**0.892860**	**and**	**12Ag**	**-**	**0.136196**
**MO 165:**	**15P**	**-**	**0.907604**	**and**	**12Ag**	**-**	**0.121854**
**MO 164:**	**14P**	**-**	**0.898791**	**and**	**12Ag**	**-**	**0.117981**
MO 163:	11H	-	0.485932	and	8C	-	0.508478
MO 162:	10H	-	0.491269	and	8C	-	0.505523
MO 161:	9H	-	0.484115	and	8C	-	0.511737
MO 160:	7H	-	0.490998	and	4C	-	0.507466
MO 159:	6H	-	0.488856	and	4C	-	0.515023
MO 158:	5H	-	0.483173	and	4C	-	0.520591
MO 157:	3H	-	0.486943	and	0C	-	0.515940
MO 156:	2H	-	0.487549	and	0C	-	0.514678
MO 155:	1H	-	0.490505	and	0C	-	0.512030

More delocalized orbitals:

MO 392: 22C - 0.189 51C - 0.491 69C - 0.268

MO 391: 49C - 0.445 151C - 0.376

MO 390: 21C - 0.519 32C - 0.225 101C - 0.184

MO 389: 52C - 0.260 69C - 0.186 114C - 0.497

MO 388: 127C - 0.386 131C - 0.436 134C - 0.078

MO 387: 34C - 0.119 71C - 0.468 72C - 0.358

MO 386: 62C - 0.155 87C - 0.265 133C - 0.506

MO 385: 66C - 0.330 89C - 0.474 111C - 0.125

```
MO 384:  22C - 0.179 23C - 0.554 52C - 0.195
MO 383:  29C - 0.557 30C - 0.225 76C - 0.138
MO 382:  28C - 0.507 78C - 0.287 91C - 0.124
MO 381:  44C - 0.131 109C - 0.477 111C - 0.325
MO 380:  129C - 0.251 141C - 0.492 153C - 0.200
MO 379:  62C - 0.285 125C - 0.165 138C - 0.488
MO 378:  31C - 0.481 55C - 0.132 76C - 0.321
MO 377:  32C - 0.206 47C - 0.246 60C - 0.494
MO 376:  35C - 0.350 72C - 0.103 113C - 0.472
MO 375:  64C - 0.478 78C - 0.145 136C - 0.313
MO 374:  37C - 0.205 80C - 0.498 153C - 0.242
MO 373:  115C - 0.440 134C - 0.386 151C - 0.080
MO 372:  44C - 0.307 54C - 0.495 66C - 0.115
MO 371:  91C - 0.318 103C - 0.481 136C - 0.135
MO 370:  37C - 0.227 50C - 0.518 129C - 0.184
MO 369:  57C - 0.491 87C - 0.172 125C - 0.279
MO 368:  47C - 0.195 97C - 0.492 101C - 0.257
MO 367:  25C - 0.555 34C - 0.240 35C - 0.118
MO 366:  30C - 0.140 55C - 0.324 68C - 0.481
```

Foster-Boys analysis for 3-Y

```
------------------------------------------------------------------------------
                           ORCA ORBITAL LOCALIZATION
------------------------------------------------------------------------------

Input orbitals are from                   ... Y_Ag.gbw
Output orbitals are to                    ... Y_NewBoys.loc.gbw
Max. number of iterations                 ... 1000
Localizations seeded randomly             ... on
Convergence tolerance                     ... 1.000e-06
Treshold for strong local MOs             ... 9.500e-01
Treshold for bond MOs                     ... 8.500e-01
Operator                                  ... 0
Orbital range for localization            ... 127 to 392
Localization criterion                    ... NEW-BOYS
Warning: cannot retrieve the overlap matrix S
        ... Overlap was succesfully recalculated
Doing the dipole integrals     ... o.k.
Initial value of the localization sum : 333441.416025
ITERATION   0 : L=334949.5979261469 DL=  1.51e+03 (AVERGE_DL)=    0.2068607547
ITERATION   1 : L=335251.4266978782 DL=  3.02e+02 (AVERGE_DL)=    0.0925404401
ITERATION   2 : L=335273.4974089620 DL=  2.21e+01 (AVERGE_DL)=    0.0250241541
ITERATION   3 : L=335275.5387146467 DL=  2.04e+00 (AVERGE_DL)=    0.0076103611
ITERATION   4 : L=335276.8598142565 DL=  1.32e+00 (AVERGE_DL)=    0.0061223623
ITERATION   5 : L=335277.6214364558 DL=  7.62e-01 (AVERGE_DL)=    0.0046485878
ITERATION   6 : L=335278.1098091388 DL=  4.88e-01 (AVERGE_DL)=    0.0037224333
ITERATION   7 : L=335278.6742850984 DL=  5.64e-01 (AVERGE_DL)=    0.0040019713
ITERATION   8 : L=335279.1406819595 DL=  4.66e-01 (AVERGE_DL)=    0.0036377182
ITERATION   9 : L=335279.3649455038 DL=  2.24e-01 (AVERGE_DL)=    0.0025224966
ITERATION  10 : L=335279.4567130532 DL=  9.18e-02 (AVERGE_DL)=    0.0016135997
ITERATION  11 : L=335279.5299654542 DL=  7.33e-02 (AVERGE_DL)=    0.0014416578
ITERATION  12 : L=335279.6847697399 DL=  1.55e-01 (AVERGE_DL)=    0.0020957657
ITERATION  13 : L=335280.0296040284 DL=  3.45e-01 (AVERGE_DL)=    0.0031279260
ITERATION  14 : L=335280.4420713024 DL=  4.12e-01 (AVERGE_DL)=    0.0034209442
ITERATION  15 : L=335280.6776781610 DL=  2.36e-01 (AVERGE_DL)=    0.0025855040
ITERATION  16 : L=335280.7621641666 DL=  8.45e-02 (AVERGE_DL)=    0.0015482591
ITERATION  17 : L=335280.7889934619 DL=  2.68e-02 (AVERGE_DL)=    0.0008724808
ITERATION  18 : L=335280.7989271886 DL=  9.93e-03 (AVERGE_DL)=    0.0005308934
ITERATION  19 : L=335280.8041534270 DL=  5.23e-03 (AVERGE_DL)=    0.0003850755
ITERATION  20 : L=335280.8078848952 DL=  3.73e-03 (AVERGE_DL)=    0.0003253802
ITERATION  21 : L=335280.8109542977 DL=  3.07e-03 (AVERGE_DL)=    0.0002951061
ITERATION  22 : L=335280.8135968175 DL=  2.64e-03 (AVERGE_DL)=    0.0002738170
ITERATION  23 : L=335280.8158983636 DL=  2.30e-03 (AVERGE_DL)=    0.0002555413
ITERATION  24 : L=335280.8179057057 DL=  2.01e-03 (AVERGE_DL)=    0.0002386503
ITERATION  25 : L=335280.8196539750 DL=  1.75e-03 (AVERGE_DL)=    0.0002227181
ITERATION  26 : L=335280.8211732839 DL=  1.52e-03 (AVERGE_DL)=    0.0002076224
ITERATION  27 : L=335280.8224907448 DL=  1.32e-03 (AVERGE_DL)=    0.0001933393
ITERATION  28 : L=335280.8236308392 DL=  1.14e-03 (AVERGE_DL)=    0.0001798546
ITERATION  29 : L=335280.8246156437 DL=  9.85e-04 (AVERGE_DL)=    0.0001671576
ITERATION  30 : L=335280.8254650049 DL=  8.49e-04 (AVERGE_DL)=    0.0001552378
ITERATION  31 : L=335280.8261965543 DL=  7.32e-04 (AVERGE_DL)=    0.0001440698
ITERATION  32 : L=335280.8268259355 DL=  6.29e-04 (AVERGE_DL)=    0.0001336313
ITERATION  33 : L=335280.8273669389 DL=  5.41e-04 (AVERGE_DL)=    0.0001238943
ITERATION  34 : L=335280.8278316139 DL=  4.65e-04 (AVERGE_DL)=    0.0001148222
ITERATION  35 : L=335280.8282305294 DL=  3.99e-04 (AVERGE_DL)=    0.0001063878
ITERATION  36 : L=335280.8285728027 DL=  3.42e-04 (AVERGE_DL)=    0.0000985457
ITERATION  37 : L=335280.8288663979 DL=  2.94e-04 (AVERGE_DL)=    0.0000912695
ITERATION  38 : L=335280.8291181704 DL=  2.52e-04 (AVERGE_DL)=    0.0000845192
```

```
ITERATION  39 : L=335280.8293340456 DL=  2.16e-04 (AVERGE_DL)=    0.0000782623
ITERATION  40 : L=335280.8295191241 DL=  1.85e-04 (AVERGE_DL)=    0.0000724651
ITERATION  41 : L=335280.8296777876 DL=  1.59e-04 (AVERGE_DL)=    0.0000670949
ITERATION  42 : L=335280.8298138064 DL=  1.36e-04 (AVERGE_DL)=    0.0000621228
ITERATION  43 : L=335280.8299304349 DL=  1.17e-04 (AVERGE_DL)=    0.0000575246
ITERATION  44 : L=335280.8300304192 DL=  1.00e-04 (AVERGE_DL)=    0.0000532620
ITERATION  45 : L=335280.8301161586 DL=  8.57e-05 (AVERGE_DL)=    0.0000493221
ITERATION  46 : L=335280.8301896931 DL=  7.35e-05 (AVERGE_DL)=    0.0000456769
ITERATION  47 : L=335280.8302527540 DL=  6.31e-05 (AVERGE_DL)=    0.0000422991
ITERATION  48 : L=335280.8303068489 DL=  5.41e-05 (AVERGE_DL)=    0.0000391769
ITERATION  49 : L=335280.8303532602 DL=  4.64e-05 (AVERGE_DL)=    0.0000362880
ITERATION  50 : L=335280.8303930887 DL=  3.98e-05 (AVERGE_DL)=    0.0000336162
ITERATION  51 : L=335280.8304272684 DL=  3.42e-05 (AVERGE_DL)=    0.0000311412
ITERATION  52 : L=335280.8304566045 DL=  2.93e-05 (AVERGE_DL)=    0.0000288505
ITERATION  53 : L=335280.8304817911 DL=  2.52e-05 (AVERGE_DL)=    0.0000267322
ITERATION  54 : L=335280.8305034162 DL=  2.16e-05 (AVERGE_DL)=    0.0000247703
ITERATION  55 : L=335280.8305219944 DL=  1.86e-05 (AVERGE_DL)=    0.0000229590
ITERATION  56 : L=335280.8305379431 DL=  1.59e-05 (AVERGE_DL)=    0.0000212723
ITERATION  57 : L=335280.8305516518 DL=  1.37e-05 (AVERGE_DL)=    0.0000197219
ITERATION  58 : L=335280.8305634219 DL=  1.18e-05 (AVERGE_DL)=    0.0000182744
ITERATION  59 : L=335280.8305735411 DL=  1.01e-05 (AVERGE_DL)=    0.0000169443
ITERATION  60 : L=335280.8305822351 DL=  8.69e-06 (AVERGE_DL)=    0.0000157058
ITERATION  61 : L=335280.8305897095 DL=  7.47e-06 (AVERGE_DL)=    0.0000145627
ITERATION  62 : L=335280.8305961317 DL=  6.42e-06 (AVERGE_DL)=    0.0000134987
ITERATION  63 : L=335280.8306016574 DL=  5.53e-06 (AVERGE_DL)=    0.0000125212
ITERATION  64 : L=335280.8306064056 DL=  4.75e-06 (AVERGE_DL)=    0.0000116069
ITERATION  65 : L=335280.8306104926 DL=  4.09e-06 (AVERGE_DL)=    0.0000107685
ITERATION  66 : L=335280.8306140062 DL=  3.51e-06 (AVERGE_DL)=    0.0000099846
ITERATION  67 : L=335280.8306170325 DL=  3.03e-06 (AVERGE_DL)=    0.0000092663
ITERATION  68 : L=335280.8306196358 DL=  2.60e-06 (AVERGE_DL)=    0.0000085943
ITERATION  69 : L=335280.8306218760 DL=  2.24e-06 (AVERGE_DL)=    0.0000079724
ITERATION  70 : L=335280.8306238009 DL=  1.92e-06 (AVERGE_DL)=    0.0000073902
ITERATION  71 : L=335280.8306254587 DL=  1.66e-06 (AVERGE_DL)=    0.0000068584
ITERATION  72 : L=335280.8306268871 DL=  1.43e-06 (AVERGE_DL)=    0.0000063660
ITERATION  73 : L=335280.8306281155 DL=  1.23e-06 (AVERGE_DL)=    0.0000059035
ITERATION  74 : L=335280.8306291743 DL=  1.06e-06 (AVERGE_DL)=    0.0000054812
ITERATION  75 : L=335280.8306300869 DL=  9.13e-07 (AVERGE_DL)=    0.0000050885
LOCALIZATION SUM CONVERGED


--------------------------------------------------------------------------------
                    LOCALIZED MOLECULAR ORBITAL COMPOSITIONS
--------------------------------------------------------------------------------

The Mulliken populations for each LMO on each atom are computed
The LMO`s will be ordered according to atom index and type
  (A) Strongly localized MO`s have populations of >=0.950 on one atom
  (B) Two center bond orbitals have populations of >=0.850 on two atoms
  (C) Other MO`s are considered to be `delocalized`

FOUND  -  32 strongly local MO`s
       - 234 two center bond MO`s
       -   0 significantly delocalized MO`s

Rather strongly localized orbitals:
MO 158:  74F  -   0.955090
MO 157:  74F  -   0.957258
MO 156:  74F  -   0.957562
MO 155:  46F  -   0.959126
MO 154:  46F  -   0.954459
MO 153:  46F  -   0.958777
MO 152:  27F  -   0.955435
MO 151:  27F  -   0.958968
MO 150:  27F  -   0.959657
MO 149:  26O  -   0.952793
MO 148:  16P  -   0.999366
MO 147:  16P  -   0.999779
MO 146:  16P  -   1.000129
MO 145:  15P  -   0.999629
MO 144:  15P  -   0.999898
MO 143:  15P  -   1.000027
MO 142:  14P  -   0.999400
MO 141:  14P  -   1.000163
MO 140:  14P  -   0.999807
MO 139:  13Y  -   1.016658
MO 138:  13Y  -   1.002456
MO 137:  13Y  -   1.002689
MO 136:  13Y  -   1.002242
MO 135:  12Ag -   0.986133
MO 134:  12Ag -   0.989990
MO 133:  12Ag -   0.996584
MO 132:  12Ag -   0.990275
```

```
MO 131:  12Ag -   0.988763
MO 130:  12Ag -   0.989071
MO 129:  12Ag -   0.995593
MO 128:  12Ag -   0.995429
MO 127:  12Ag -   0.988754
Bond-like localized orbitals:
MO 392: 183H  -   0.487340  and 180C  -   0.513273
MO 391: 182H  -   0.490189  and 180C  -   0.509718
MO 390: 181H  -   0.490859  and 180C  -   0.511155
MO 389: 180C  -   0.505423  and  75C  -   0.534436
MO 388: 179H  -   0.493540  and 176C  -   0.502423
MO 387: 178H  -   0.459094  and 176C  -   0.535865
MO 386: 177H  -   0.467081  and 176C  -   0.528428
MO 385: 176C  -   0.469321  and 163C  -   0.562327
MO 384: 175H  -   0.469509  and 172C  -   0.520712
MO 383: 174H  -   0.468287  and 172C  -   0.519311
MO 382: 173H  -   0.488682  and 172C  -   0.497243
MO 381: 172C  -   0.477305  and 163C  -   0.554121
MO 380: 171H  -   0.487722  and 168C  -   0.507444
MO 379: 170H  -   0.491890  and 168C  -   0.503300
MO 378: 169H  -   0.490162  and 168C  -   0.504521
MO 377: 168C  -   0.512411  and 163C  -   0.528800
MO 376: 167H  -   0.482577  and 164C  -   0.508073
MO 375: 166H  -   0.480349  and 164C  -   0.507771
MO 374: 165H  -   0.462837  and 164C  -   0.527969
MO 373: 164C  -   0.477478  and  99C  -   0.552500
MO 372: 163C  -   0.519263  and  51C  -   0.515211
MO 371: 162H  -   0.489156  and 159C  -   0.505895
MO 370: 161H  -   0.492124  and 159C  -   0.504185
MO 369: 160H  -   0.484894  and 159C  -   0.511421
MO 368: 159C  -   0.503823  and  75C  -   0.541354
MO 367: 158H  -   0.487291  and 155C  -   0.508959
MO 366: 157H  -   0.491544  and 155C  -   0.505472
MO 365: 156H  -   0.486853  and 155C  -   0.508146
MO 364: 155C  -   0.504675  and  59C  -   0.540684
MO 363: 154H  -   0.511357  and 153C  -   0.516259
MO 362: 153C  -   0.515514  and 141C  -   0.502741
MO 361: 153C  -   0.454846  and  80C  -   0.469828
MO 360: 153C  -   0.455254  and  80C  -   0.471689
MO 359: 152H  -   0.503487  and 151C  -   0.527675
MO 358: 151C  -   0.492535  and 115C  -   0.521390
MO 357: 151C  -   0.448152  and  49C  -   0.473968
MO 356: 151C  -   0.456620  and  49C  -   0.464094
MO 355: 150H  -   0.485729  and 147C  -   0.517727
MO 354: 149H  -   0.488774  and 147C  -   0.512887
MO 353: 148H  -   0.491122  and 147C  -   0.509872
MO 352: 147C  -   0.501971  and  59C  -   0.540218
MO 351: 146H  -   0.491165  and 143C  -   0.506168
MO 350: 145H  -   0.485452  and 143C  -   0.516796
MO 349: 144H  -   0.484742  and 143C  -   0.517604
MO 348: 143C  -   0.504556  and  59C  -   0.532556
MO 347: 142H  -   0.515909  and 141C  -   0.508979
MO 346: 141C  -   0.475200  and 129C  -   0.446970
MO 345: 141C  -   0.468350  and 129C  -   0.447079
MO 344: 140C  -   0.333648  and  74F  -   0.675020
MO 343: 140C  -   0.327339  and  46F  -   0.678052
MO 342: 140C  -   0.323532  and  27F  -   0.678188
MO 341: 140C  -   0.505418  and  17S  -   0.550191
MO 340: 139H  -   0.511513  and 138C  -   0.514694
MO 339: 138C  -   0.463361  and 125C  -   0.457513
MO 338: 138C  -   0.467681  and 125C  -   0.458879
MO 337: 138C  -   0.515047  and  62C  -   0.497761
MO 336: 137H  -   0.510566  and 136C  -   0.517427
MO 335: 136C  -   0.510189  and 103C  -   0.508465
MO 334: 136C  -   0.461306  and  64C  -   0.462975
MO 333: 136C  -   0.459986  and  64C  -   0.466862
MO 332: 135H  -   0.511333  and 134C  -   0.517781
MO 331: 134C  -   0.514627  and 131C  -   0.504228
MO 330: 134C  -   0.454432  and 115C  -   0.476297
MO 329: 134C  -   0.469443  and 115C  -   0.456542
MO 328: 133C  -   0.509102  and  87C  -   0.502912
MO 327: 133C  -   0.484332  and  62C  -   0.438128
MO 326: 133C  -   0.472352  and  62C  -   0.441248
MO 325: 133C  -   0.551596  and  15P  -   0.453699
MO 324: 132H  -   0.511731  and 131C  -   0.514899
MO 323: 131C  -   0.463148  and 127C  -   0.455393
MO 322: 131C  -   0.466828  and 127C  -   0.458824
MO 321: 130H  -   0.508414  and 129C  -   0.524718
MO 320: 129C  -   0.495378  and  50C  -   0.518538
MO 319: 128H  -   0.519975  and 127C  -   0.502450
MO 318: 127C  -   0.489272  and  49C  -   0.517759
MO 317: 126H  -   0.509155  and 125C  -   0.517869
```

```
MO 316: 125C  -   0.513779  and  57C  -   0.505847
MO 315: 124H  -   0.485442  and 121C  -   0.517995
MO 314: 123H  -   0.491341  and 121C  -   0.509507
MO 313: 122H  -   0.488609  and 121C  -   0.512805
MO 312: 121C  -   0.501733  and  75C  -   0.540049
MO 311: 120H  -   0.489068  and 117C  -   0.505585
MO 310: 119H  -   0.489195  and 117C  -   0.507028
MO 309: 118H  -   0.491651  and 117C  -   0.504741
MO 308: 117C  -   0.512437  and  99C  -   0.530969
MO 307: 116H  -   0.511540  and 115C  -   0.513728
MO 306: 114C  -   0.501966  and  75C  -   0.535596
MO 305: 114C  -   0.527685  and  69C  -   0.496139
MO 304: 114C  -   0.468612  and  52C  -   0.460622
MO 303: 114C  -   0.465250  and  52C  -   0.467723
MO 302: 113C  -   0.517728  and  72C  -   0.504398
MO 301: 113C  -   0.500990  and  59C  -   0.537655
MO 300: 113C  -   0.464015  and  35C  -   0.477356
MO 299: 113C  -   0.470546  and  35C  -   0.466979
MO 298: 112H  -   0.509447  and 111C  -   0.516730
MO 297: 111C  -   0.457324  and 109C  -   0.470993
MO 296: 111C  -   0.461778  and 109C  -   0.465533
MO 295: 111C  -   0.514366  and  89C  -   0.504509
MO 294: 110H  -   0.512937  and 109C  -   0.514402
MO 293: 109C  -   0.517130  and  44C  -   0.495252
MO 292: 108H  -   0.490494  and 105C  -   0.505719
MO 291: 107H  -   0.485890  and 105C  -   0.509443
MO 290: 106H  -   0.491609  and 105C  -   0.501571
MO 289: 105C  -   0.510755  and 100C  -   0.528598
MO 288: 104H  -   0.509580  and 103C  -   0.515646
MO 287: 103C  -   0.470646  and  91C  -   0.452401
MO 286: 103C  -   0.474549  and  91C  -   0.452618
MO 285: 102H  -   0.523290  and 101C  -   0.510475
MO 284: 101C  -   0.451897  and  97C  -   0.471998
MO 283: 101C  -   0.450283  and  97C  -   0.466446
MO 282: 101C  -   0.496414  and  21C  -   0.515008
MO 281: 100C  -   0.560648  and  93C  -   0.470644
MO 280: 100C  -   0.556114  and  83C  -   0.476793
MO 279: 100C  -   0.516412  and  71C  -   0.518373
MO 278:  99C  -   0.522855  and  68C  -   0.512897
MO 277:  99C  -   0.560069  and  40C  -   0.471243
MO 276:  98H  -   0.517859  and  97C  -   0.510321
MO 275:  97C  -   0.503891  and  47C  -   0.515422
MO 274:  96H  -   0.489770  and  93C  -   0.494717
MO 273:  95H  -   0.455411  and  93C  -   0.535933
MO 272:  94H  -   0.473743  and  93C  -   0.512058
MO 271:  92H  -   0.510594  and  91C  -   0.518796
MO 270:  91C  -   0.505387  and  28C  -   0.500001
MO 269:  90H  -   0.508771  and  89C  -   0.517874
MO 268:  89C  -   0.473735  and  66C  -   0.452235
MO 267:  89C  -   0.471717  and  66C  -   0.452099
MO 266:  88H  -   0.500841  and  87C  -   0.527652
MO 265:  87C  -   0.445236  and  57C  -   0.477563
MO 264:  87C  -   0.450586  and  57C  -   0.469149
MO 263:  86H  -   0.462661  and  83C  -   0.531279
MO 262:  85H  -   0.471602  and  83C  -   0.522471
MO 261:  84H  -   0.491203  and  83C  -   0.503614
MO 260:  82C  -   0.536464  and  31C  -   0.503556
MO 259:  82C  -   0.540451  and   8C  -   0.502008
MO 258:  82C  -   0.532355  and   4C  -   0.508624
MO 257:  82C  -   0.539799  and   0C  -   0.501190
MO 256:  81H  -   0.509346  and  80C  -   0.515530
MO 255:  80C  -   0.521801  and  37C  -   0.493804
MO 254:  79H  -   0.522260  and  78C  -   0.514365
MO 253:  78C  -   0.489407  and  64C  -   0.523138
MO 252:  78C  -   0.428422  and  28C  -   0.501908
MO 251:  78C  -   0.445205  and  28C  -   0.479455
MO 250:  77H  -   0.524657  and  76C  -   0.507584
MO 249:  76C  -   0.470554  and  31C  -   0.465872
MO 248:  76C  -   0.468760  and  31C  -   0.466669
MO 247:  76C  -   0.481470  and  29C  -   0.511426
MO 246:  73H  -   0.526617  and  72C  -   0.508281
MO 245:  72C  -   0.482848  and  71C  -   0.449153
MO 244:  72C  -   0.471581  and  71C  -   0.456701
MO 243:  71C  -   0.511556  and  34C  -   0.514927
MO 242:  70H  -   0.524282  and  69C  -   0.511012
MO 241:  69C  -   0.467161  and  51C  -   0.456057
MO 240:  69C  -   0.455719  and  51C  -   0.466167
MO 239:  68C  -   0.444022  and  55C  -   0.476109
MO 238:  68C  -   0.466935  and  55C  -   0.465561
MO 237:  68C  -   0.511473  and  30C  -   0.518640
MO 236:  67H  -   0.503346  and  66C  -   0.524752
MO 235:  66C  -   0.506563  and  54C  -   0.502491
```

MO 234:	65H	-	0.513873	and	64C	-	0.513926
MO 233:	63H	-	0.509229	and	62C	-	0.511476
MO 232:	61H	-	0.507873	and	60C	-	0.516615
MO 231:	60C	-	0.471239	and	47C	-	0.452632
MO 230:	60C	-	0.464459	and	47C	-	0.460860
MO 229:	60C	-	0.521865	and	32C	-	0.492486
MO 228:	58H	-	0.508752	and	57C	-	0.516762
MO 227:	56H	-	0.525356	and	55C	-	0.508347
MO 226:	55C	-	0.509412	and	31C	-	0.512931
MO 225:	54C	-	0.475894	and	44C	-	0.446006
MO 224:	54C	-	0.479538	and	44C	-	0.443341
MO 223:	54C	-	0.546765	and	16P	-	0.461617
MO 222:	53H	-	0.520571	and	52C	-	0.511356
MO 221:	52C	-	0.506709	and	23C	-	0.486443
MO 220:	51C	-	0.499895	and	22C	-	0.529378
MO 219:	50C	-	0.474878	and	37C	-	0.445007
MO 218:	50C	-	0.486444	and	37C	-	0.434088
MO 217:	50C	-	0.525613	and	16P	-	0.480815
MO 216:	49C	-	0.544811	and	14P	-	0.455748
MO 215:	48H	-	0.510887	and	47C	-	0.517222
MO 214:	45H	-	0.511384	and	44C	-	0.512118
MO 213:	43H	-	0.452230	and	40C	-	0.542665
MO 212:	42H	-	0.478999	and	40C	-	0.517576
MO 211:	41H	-	0.491719	and	40C	-	0.503251
MO 210:	39O	-	0.831140	and	17S	-	0.169967
MO 209:	39O	-	0.700960	and	17S	-	0.292534
MO 208:	39O	-	0.735132	and	17S	-	0.266564
**MO 207:**	**39O**	**-**	**0.939355**	**and**	**13Y**	**-**	**0.035521**
MO 206:	38H	-	0.498178	and	37C	-	0.529623
MO 205:	36H	-	0.515272	and	35C	-	0.515710
MO 204:	35C	-	0.484351	and	25C	-	0.509770
MO 203:	34C	-	0.378828	and	25C	-	0.548022
MO 202:	34C	-	0.383190	and	25C	-	0.533204
MO 201:	34C	-	0.374495	and	18O	-	0.620181
MO 200:	33H	-	0.497145	and	32C	-	0.528062
MO 199:	32C	-	0.427428	and	21C	-	0.492268
MO 198:	32C	-	0.440678	and	21C	-	0.478801
MO 197:	30C	-	0.392851	and	29C	-	0.533291
MO 196:	30C	-	0.369939	and	29C	-	0.553737
MO 195:	30C	-	0.379249	and	19O	-	0.619994
MO 194:	29C	-	0.507760	and	16P	-	0.492830
MO 193:	28C	-	0.526347	and	14P	-	0.477646
MO 192:	26O	-	0.784844	and	17S	-	0.213127
MO 191:	26O	-	0.764055	and	17S	-	0.235295
MO 190:	26O	-	0.698443	and	17S	-	0.300029
MO 189:	25C	-	0.501242	and	14P	-	0.494092
MO 188:	24O	-	0.624772	and	17S	-	0.368204
MO 187:	24O	-	0.906707	and	17S	-	0.050850
**MO 186:**	**24O**	**-**	**0.880511**	**and**	**13Y**	**-**	**0.060852**
**MO 185:**	**24O**	**-**	**0.894880**	**and**	**13Y**	**-**	**0.062139**
MO 184:	23C	-	0.526637	and	22C	-	0.388394
MO 183:	23C	-	0.553510	and	22C	-	0.364070
MO 182:	23C	-	0.506828	and	15P	-	0.496704
MO 181:	22C	-	0.376941	and	20O	-	0.619468
MO 180:	21C	-	0.527444	and	15P	-	0.471181
**MO 179:**	**20O**	**-**	**0.895969**	**and**	**13Y**	**-**	**0.088320**
**MO 178:**	**20O**	**-**	**0.892828**	**and**	**13Y**	**-**	**0.086745**
**MO 177:**	**20O**	**-**	**0.846582**	**and**	**12Ag**	**-**	**0.066096**
**MO 176:**	**19O**	**-**	**0.855620**	**and**	**13Y**	**-**	**0.069074**
**MO 175:**	**19O**	**-**	**0.885735**	**and**	**13Y**	**-**	**0.066429**
**MO 174:**	**19O**	**-**	**0.892051**	**and**	**13Y**	**-**	**0.074295**
**MO 173:**	**18O**	**-**	**0.888742**	**and**	**13Y**	**-**	**0.086736**
**MO 172:**	**18O**	**-**	**0.901163**	**and**	**13Y**	**-**	**0.074354**
**MO 171:**	**18O**	**-**	**0.845373**	**and**	**12Ag**	**-**	**0.052467**
**MO 170:**	**16P**	**-**	**0.862726**	**and**	**12Ag**	**-**	**0.147324**
**MO 169:**	**15P**	**-**	**0.877824**	**and**	**12Ag**	**-**	**0.132705**
**MO 168:**	**14P**	**-**	**0.872933**	**and**	**12Ag**	**-**	**0.123595**
MO 167:	11H	-	0.487254	and	8C	-	0.506381
MO 166:	10H	-	0.491838	and	8C	-	0.504283
MO 165:	9H	-	0.485177	and	8C	-	0.510001
MO 164:	7H	-	0.491422	and	4C	-	0.506543
MO 163:	6H	-	0.489523	and	4C	-	0.513770
MO 162:	5H	-	0.484019	and	4C	-	0.518847
MO 161:	3H	-	0.487936	and	0C	-	0.514066
MO 160:	2H	-	0.488312	and	0C	-	0.513222
MO 159:	1H	-	0.490822	and	0C	-	0.511315

IBO analysis for 3-Y

```
--------------------------------------------------------------------------------
                           ORCA ORBITAL LOCALIZATION
--------------------------------------------------------------------------------

Input orbitals are from                  ... Y_Ag.gbw
Output orbitals are to                   ... Y_IBO.loc.gbw
Max. number of iterations                ... 1000
Localizations seeded randomly            ... on
Convergence tolerance                    ... 1.000e-06
Treshold for strong local MOs            ... 9.500e-01
Treshold for bond MOs                    ... 8.500e-01
Operator                                 ... 0
Orbital range for localization           ... 127 to 392
Localization criterion                   ... IAO-IBO
Warning: cannot retrieve the overlap matrix S
        ... Overlap was succesfully recalculated
Entering Jacobi type localization:
Using Cholesky decomposition as initial guess.
Your calculation utilizes the IAOIBO localization method
Cite in your paper:
G. KNIZIA, INTRISIC ATOMIC ORBITALS: AN UNBIASED BRIDGE BETWEEN
QUANTUM THEORY AND CHEMICAL CONCEPTS
J. CHEM. THEOTY COMPUT. 2013, 9, 4834~4843.

-------------------
IAO PARTIAL CHARGES
-------------------
Warning!!! IAOs HAVE MEANING, only when all occupied MOs are involved.
   0 C :    1.282982
   1 H :    0.233611
   2 H :    0.235593
   3 H :    0.240856
   4 C :    1.288716
   5 H :    0.233624
   6 H :    0.230914
   7 H :    0.235053
   8 C :    1.276061
   9 H :    0.238918
  10 H :    0.239778
  11 H :    0.235119
  12 Ag:   28.525258
  13 Y :   29.174244
  14 P :    4.494059
  15 P :    4.500671
  16 P :    4.509575
  17 S :   12.103462
  18 O :    1.490259
  19 O :    1.495279
  20 O :    1.488672
  21 C :    1.827792
  22 C :    2.198134
  23 C :    1.761732
  24 O :    1.171242
  25 C :    1.750353
  26 O :    1.147825
  27 F :    1.759944
  28 C :    1.824759
  29 C :    1.754957
  30 C :    2.202678
  31 C :    1.990097
  32 C :    1.755914
  33 H :    0.262883
  34 C :    2.209016
  35 C :    1.728010
  36 H :    0.234149
  37 C :    1.750284
  38 H :    0.260841
  39 O :    1.137382
  40 C :    1.286600
  41 H :    0.237436
  42 H :    0.234505
  43 H :    0.230858
  44 C :    1.759962
  45 H :    0.236432
  46 F :    1.754298
  47 C :    1.746568
  48 H :    0.262074
  49 C :    1.823002
  50 C :    1.822942
```

```
 51 C :    1.975171
 52 C :    1.725729
 53 H :    0.237623
 54 C :    1.821612
 55 C :    1.737828
 56 H :    0.227728
 57 C :    1.743719
 58 H :    0.262810
 59 C :    2.065806
 60 C :    1.747713
 61 H :    0.265115
 62 C :    1.754790
 63 H :    0.245856
 64 C :    1.757044
 65 H :    0.255624
 66 C :    1.750809
 67 H :    0.260439
 68 C :    1.974147
 69 C :    1.735864
 70 H :    0.231264
 71 C :    1.977593
 72 C :    1.741289
 73 H :    0.227349
 74 F :    1.754666
 75 C :    2.066043
 76 C :    1.731493
 77 H :    0.236931
 78 C :    1.765646
 79 H :    0.250820
 80 C :    1.744717
 81 H :    0.263675
 82 C :    2.065951
 83 C :    1.289194
 84 H :    0.236297
 85 H :    0.241598
 86 H :    0.218723
 87 C :    1.745085
 88 H :    0.261542
 89 C :    1.744910
 90 H :    0.264012
 91 C :    1.754786
 92 H :    0.256623
 93 C :    1.305133
 94 H :    0.197465
 95 H :    0.249411
 96 H :    0.230672
 97 C :    1.759054
 98 H :    0.252476
 99 C :    2.065382
100 C :    2.067046
101 C :    1.761734
102 H :    0.246030
103 C :    1.744188
104 H :    0.262641
105 C :    1.281648
106 H :    0.226637
107 H :    0.246952
108 H :    0.232502
109 C :    1.745064
110 H :    0.262940
111 C :    1.747380
112 H :    0.263285
113 C :    1.988296
114 C :    1.988738
115 C :    1.741741
116 H :    0.260969
117 C :    1.281397
118 H :    0.230688
119 H :    0.242581
120 H :    0.230151
121 C :    1.282132
122 H :    0.232888
123 H :    0.235287
124 H :    0.242690
125 C :    1.743883
126 H :    0.261283
127 C :    1.754410
128 H :    0.244280
129 C :    1.759877
130 H :    0.256076
131 C :    1.753157
132 H :    0.261226
```

```
 133 C :    1.823356
 134 C :    1.739490
 135 H :    0.259890
 136 C :    1.748029
 137 H :    0.263099
 138 C :    1.746809
 139 H :    0.264375
 140 C :    2.551422
 141 C :    1.762741
 142 H :    0.249383
 143 C :    1.287575
 144 H :    0.234123
 145 H :    0.230079
 146 H :    0.236771
 147 C :    1.282321
 148 H :    0.234857
 149 H :    0.233391
 150 H :    0.242347
 151 C :    1.738211
 152 H :    0.257894
 153 C :    1.745999
 154 H :    0.261316
 155 C :    1.278021
 156 H :    0.234346
 157 H :    0.239138
 158 H :    0.235190
 159 C :    1.277617
 160 H :    0.238571
 161 H :    0.239680
 162 H :    0.231871
 163 C :    2.066214
 164 C :    1.300966
 165 H :    0.208534
 166 H :    0.238248
 167 H :    0.239205
 168 C :    1.282391
 169 H :    0.230939
 170 H :    0.227320
 171 H :    0.244715
 172 C :    1.305539
 173 H :    0.232752
 174 H :    0.201493
 175 H :    0.244857
 176 C :    1.285207
 177 H :    0.246098
 178 H :    0.223971
 179 H :    0.234691
 180 C :    1.289387
 181 H :    0.228091
 182 H :    0.235040
 183 H :    0.236033
Sum of atomic charges:  254.0000000

Initial value of the localization sum :    49.061192
ITERATION   0 : L=   71.5222010137 DL=  2.25e+01 (MAX-T)=  44.971
ITERATION   1 : L=   72.3266588102 DL=  8.04e-01 (MAX-T)=  42.691
ITERATION   2 : L=   72.3476007011 DL=  2.09e-02 (MAX-T)=  39.804
ITERATION   3 : L=   72.3476880220 DL=  8.73e-05 (MAX-T)=   1.074
ITERATION   4 : L=   72.3476897480 DL=  1.73e-06 (MAX-T)=   0.057
ITERATION   5 : L=   72.3476898130 DL=  6.50e-08 (MAX-T)=   0.014
LOCALIZATION SUM CONVERGED


--------------------------------------------------------------------------------
                    LOCALIZED MOLECULAR ORBITAL COMPOSITIONS
--------------------------------------------------------------------------------

The Mulliken populations for each LMO on each atom are computed
The LMO`s will be ordered according to atom index and type
  (A) Strongly localized MO`s have populations of >=0.950 on one atom
  (B) Two center bond orbitals have populations of >=0.850 on two atoms
  (C) Other MO`s are considered to be `delocalized`

FOUND  -  27 strongly local MO`s
       - 212 two center bond MO`s
       -  27 significantly delocalized MO`s

Rather strongly localized orbitals:
MO 153:  74F  -   0.989321
MO 152:  46F  -   0.990971
MO 151:  39O  -   0.993362
MO 150:  27F  -   0.990826
```

```
MO 149:  26O  -   1.001402
MO 148:  16P  -   1.000053
MO 147:  16P  -   0.999899
MO 146:  16P  -   0.998920
MO 145:  15P  -   0.999936
MO 144:  15P  -   0.999998
MO 143:  15P  -   0.999158
MO 142:  14P  -   0.999004
MO 141:  14P  -   0.999879
MO 140:  14P  -   1.000114
MO 139:  13Y  -   1.006492
MO 138:  13Y  -   1.006376
MO 137:  13Y  -   1.006672
MO 136:  13Y  -   1.013127
MO 135:  12Ag -   1.004572
MO 134:  12Ag -   0.990299
MO 133:  12Ag -   0.992516
MO 132:  12Ag -   0.990172
MO 131:  12Ag -   0.979541
MO 130:  12Ag -   0.999934
MO 129:  12Ag -   1.002306
MO 128:  12Ag -   1.001718
MO 127:  12Ag -   0.976599
Bond-like localized orbitals:
MO 365: 183H  -   0.485305  and 180C  -   0.514903
MO 364: 182H  -   0.487814  and 180C  -   0.511289
MO 363: 181H  -   0.489304  and 180C  -   0.512125
MO 362: 180C  -   0.511041  and  75C  -   0.528517
MO 361: 179H  -   0.491998  and 176C  -   0.503577
MO 360: 178H  -   0.458670  and 176C  -   0.534689
MO 359: 177H  -   0.465295  and 176C  -   0.529478
MO 358: 176C  -   0.473624  and 163C  -   0.557236
MO 357: 175H  -   0.466360  and 172C  -   0.523606
MO 356: 174H  -   0.469927  and 172C  -   0.515356
MO 355: 173H  -   0.486007  and 172C  -   0.500046
MO 354: 172C  -   0.481263  and 163C  -   0.548801
MO 353: 171H  -   0.485175  and 168C  -   0.509326
MO 352: 170H  -   0.489996  and 168C  -   0.504571
MO 351: 169H  -   0.488090  and 168C  -   0.506169
MO 350: 168C  -   0.513541  and 163C  -   0.528512
MO 349: 167H  -   0.479970  and 164C  -   0.510634
MO 348: 166H  -   0.477857  and 164C  -   0.509833
MO 347: 165H  -   0.463527  and 164C  -   0.525443
MO 346: 164C  -   0.481169  and  99C  -   0.547888
MO 345: 163C  -   0.517856  and  51C  -   0.515422
MO 344: 162H  -   0.487255  and 159C  -   0.507231
MO 343: 161H  -   0.490167  and 159C  -   0.505314
MO 342: 160H  -   0.482386  and 159C  -   0.513594
MO 341: 159C  -   0.504971  and  75C  -   0.541137
MO 340: 158H  -   0.484968  and 155C  -   0.510787
MO 339: 157H  -   0.489581  and 155C  -   0.506556
MO 338: 156H  -   0.484427  and 155C  -   0.510186
MO 337: 155C  -   0.505744  and  59C  -   0.540663
MO 336: 154H  -   0.504699  and 153C  -   0.506916
MO 335: 153C  -   0.517706  and 141C  -   0.503633
MO 334: 153C  -   0.514511  and  80C  -   0.507495
MO 333: 152H  -   0.496669  and 151C  -   0.516883
MO 332: 151C  -   0.498161  and 115C  -   0.519691
MO 331: 151C  -   0.505972  and  49C  -   0.511206
MO 330: 150H  -   0.483244  and 147C  -   0.519641
MO 329: 149H  -   0.487121  and 147C  -   0.513843
MO 328: 148H  -   0.489092  and 147C  -   0.510936
MO 327: 147C  -   0.506790  and  59C  -   0.535558
MO 326: 146H  -   0.488657  and 143C  -   0.507934
MO 325: 145H  -   0.483849  and 143C  -   0.517678
MO 324: 144H  -   0.482653  and 143C  -   0.519091
MO 323: 143C  -   0.509785  and  59C  -   0.527289
MO 322: 142H  -   0.508995  and 141C  -   0.501052
MO 321: 141C  -   0.515567  and 129C  -   0.500063
MO 320: 140C  -   0.319871  and  74F  -   0.687844
MO 319: 140C  -   0.314951  and  46F  -   0.689542
MO 318: 140C  -   0.311657  and  27F  -   0.689429
MO 317: 140C  -   0.505976  and  17S  -   0.575103
MO 316: 139H  -   0.504914  and 138C  -   0.505113
MO 315: 138C  -   0.505690  and 125C  -   0.516921
MO 314: 138C  -   0.514129  and  62C  -   0.502101
MO 313: 137H  -   0.503983  and 136C  -   0.508151
MO 312: 136C  -   0.512199  and 103C  -   0.509556
MO 311: 136C  -   0.521458  and  64C  -   0.500473
MO 310: 135H  -   0.504381  and 134C  -   0.508177
MO 309: 134C  -   0.516276  and 131C  -   0.505525
MO 308: 134C  -   0.513183  and 115C  -   0.508777
```

```
MO 307: 133C  -   0.507169  and  87C  -   0.511120
MO 306: 133C  -   0.513270  and  62C  -   0.501982
MO 305: 133C  -   0.556100  and  15P  -   0.446579
MO 304: 132H  -   0.504871  and 131C  -   0.505513
MO 303: 131C  -   0.512169  and 127C  -   0.505261
MO 302: 130H  -   0.501042  and 129C  -   0.514262
MO 301: 129C  -   0.502980  and  50C  -   0.516053
MO 300: 128H  -   0.512418  and 127C  -   0.494190
MO 299: 127C  -   0.495872  and  49C  -   0.515967
MO 298: 126H  -   0.502407  and 125C  -   0.509021
MO 297: 125C  -   0.515407  and  57C  -   0.507384
MO 296: 124H  -   0.482826  and 121C  -   0.519973
MO 295: 123H  -   0.489212  and 121C  -   0.510671
MO 294: 122H  -   0.487067  and 121C  -   0.513597
MO 293: 121C  -   0.506314  and  75C  -   0.536117
MO 292: 120H  -   0.487191  and 117C  -   0.507050
MO 291: 119H  -   0.487059  and 117C  -   0.508413
MO 290: 118H  -   0.489719  and 117C  -   0.506214
MO 289: 117C  -   0.514180  and  99C  -   0.529759
MO 288: 116H  -   0.504530  and 115C  -   0.504706
MO 287: 114C  -   0.503720  and  75C  -   0.533396
MO 286: 114C  -   0.522782  and  69C  -   0.509621
MO 285: 114C  -   0.490910  and  52C  -   0.540133
MO 284: 113C  -   0.511296  and  72C  -   0.518516
MO 283: 113C  -   0.502676  and  59C  -   0.535604
MO 282: 113C  -   0.491592  and  35C  -   0.543101
MO 281: 112H  -   0.502534  and 111C  -   0.507984
MO 280: 111C  -   0.518154  and 109C  -   0.503664
MO 279: 111C  -   0.515873  and  89C  -   0.505928
MO 278: 110H  -   0.506313  and 109C  -   0.504503
MO 277: 109C  -   0.516333  and  44C  -   0.499729
MO 276: 108H  -   0.488319  and 105C  -   0.507399
MO 275: 107H  -   0.483089  and 105C  -   0.511467
MO 274: 106H  -   0.489808  and 105C  -   0.502671
MO 273: 105C  -   0.511612  and 100C  -   0.528762
MO 272: 104H  -   0.503002  and 103C  -   0.507090
MO 271: 103C  -   0.513381  and  91C  -   0.504193
MO 270: 102H  -   0.515450  and 101C  -   0.501139
MO 269: 101C  -   0.503071  and  97C  -   0.511574
MO 268: 101C  -   0.503678  and  21C  -   0.512291
MO 267: 100C  -   0.554641  and  93C  -   0.475036
MO 266: 100C  -   0.551651  and  83C  -   0.480662
MO 265: 100C  -   0.514630  and  71C  -   0.518532
MO 264:  99C  -   0.521413  and  68C  -   0.513136
MO 263:  99C  -   0.554361  and  40C  -   0.475964
MO 262:  98H  -   0.510880  and  97C  -   0.501368
MO 261:  97C  -   0.504965  and  47C  -   0.517466
MO 260:  96H  -   0.487241  and  93C  -   0.497467
MO 259:  95H  -   0.452260  and  93C  -   0.538541
MO 258:  94H  -   0.475564  and  93C  -   0.507464
MO 257:  92H  -   0.503761  and  91C  -   0.509909
MO 256:  91C  -   0.514335  and  28C  -   0.495702
MO 255:  90H  -   0.502411  and  89C  -   0.508871
MO 254:  89C  -   0.516128  and  66C  -   0.502700
MO 253:  88H  -   0.493895  and  87C  -   0.517494
MO 252:  87C  -   0.498898  and  57C  -   0.519445
MO 251:  86H  -   0.462271  and  83C  -   0.529658
MO 250:  85H  -   0.469646  and  83C  -   0.523631
MO 249:  84H  -   0.489270  and  83C  -   0.505058
MO 248:  82C  -   0.533816  and  31C  -   0.505381
MO 247:  82C  -   0.541499  and   8C  -   0.502311
MO 246:  82C  -   0.527608  and   4C  -   0.513443
MO 245:  82C  -   0.534326  and   0C  -   0.506609
MO 244:  81H  -   0.502744  and  80C  -   0.506314
MO 243:  80C  -   0.520423  and  37C  -   0.499147
MO 242:  79H  -   0.513476  and  78C  -   0.502997
MO 241:  78C  -   0.494823  and  64C  -   0.521442
MO 240:  78C  -   0.502618  and  28C  -   0.522486
MO 239:  77H  -   0.516689  and  76C  -   0.496923
MO 238:  76C  -   0.544857  and  31C  -   0.490240
MO 237:  76C  -   0.492026  and  29C  -   0.509132
MO 236:  74F  -   0.933965  and  17S  -   0.007904
MO 235:  74F  -   0.933659  and  13Y  -   0.006785
MO 234:  73H  -   0.519032  and  72C  -   0.498129
MO 233:  72C  -   0.551121  and  71C  -   0.469689
MO 232:  71C  -   0.507752  and  34C  -   0.526641
MO 231:  70H  -   0.516733  and  69C  -   0.500409
MO 230:  69C  -   0.532292  and  51C  -   0.488225
MO 229:  68C  -   0.473197  and  55C  -   0.546176
MO 228:  68C  -   0.507270  and  30C  -   0.531261
MO 227:  67H  -   0.496560  and  66C  -   0.515334
MO 226:  66C  -   0.515171  and  54C  -   0.499299
```

MO 225: 65H - 0.507010 and 64C - 0.504661
MO 224: 63H - 0.501638 and 62C - 0.503704
MO 223: 61H - 0.501141 and 60C - 0.507381
MO 222: 60C - 0.505916 and 47C - 0.515999
MO 221: 60C - 0.520401 and 32C - 0.497703
MO 220: 58H - 0.502166 and 57C - 0.507703
MO 219: 56H - 0.518149 and 55C - 0.498593
MO 218: 55C - 0.524096 and 31C - 0.506359
MO 217: 54C - 0.506090 and 44C - 0.510399
MO 216: 54C - 0.551716 and 16P - 0.454479
MO 215: 53H - 0.513211 and 52C - 0.500537
MO 214: 52C - 0.516498 and 23C - 0.485666
MO 213: 51C - 0.496225 and 22C - 0.541740
MO 212: 50C - 0.510184 and 37C - 0.504388
MO 211: 50C - 0.529897 and 16P - 0.473825
MO 210: 49C - 0.544941 and 14P - 0.455493
MO 209: 48H - 0.504443 and 47C - 0.507886
MO 208: 46F - 0.933819 and 42H - 0.007284
MO 207: 46F - 0.936127 and 17S - 0.008746
MO 206: 45H - 0.503612 and 44C - 0.503920
MO 205: 43H - 0.451203 and 40C - 0.542209
MO 204: 42H - 0.478547 and 40C - 0.516969
MO 203: 41H - 0.490034 and 40C - 0.504592
MO 202: 39O - 0.838019 and 17S - 0.156965
MO 201: 39O - 0.552105 and 17S - 0.451941
MO 200: 39O - 0.827298 and 17S - 0.140675
MO 199: 38H - 0.491477 and 37C - 0.519368
MO 198: 36H - 0.508344 and 35C - 0.505561
MO 197: 35C - 0.495121 and 25C - 0.507441
MO 196: 34C - 0.482997 and 25C - 0.541827
MO 195: 34C - 0.367233 and 18O - 0.631753
MO 194: 33H - 0.490053 and 32C - 0.518016
MO 193: 32C - 0.498125 and 21C - 0.516115
MO 192: 30C - 0.479042 and 29C - 0.551299
MO 191: 30C - 0.369007 and 19O - 0.631022
MO 190: 29C - 0.505443 and 16P - 0.493103
MO 189: 28C - 0.530555 and 14P - 0.471391
MO 188: 27F - 0.936740 and 17S - 0.009969
MO 187: 27F - 0.935580 and 13Y - 0.000573
MO 186: 26O - 0.545922 and 17S - 0.456264
MO 185: 26O - 0.833969 and 17S - 0.157834
MO 184: 26O - 0.824109 and 17S - 0.148808
MO 183: 25C - 0.499769 and 14P - 0.493470
MO 182: 24O - 0.623327 and 17S - 0.373977
MO 181: 24O - 0.870914 and 17S - 0.080239
MO 180: 24O - 0.887054 and 13Y - 0.068201
MO 179: 24O - 0.924954 and 13Y - 0.047098
MO 178: 23C - 0.553711 and 22C - 0.471189
MO 177: 23C - 0.510450 and 15P - 0.488824
MO 176: 22C - 0.366492 and 20O - 0.633012
MO 175: 21C - 0.532287 and 15P - 0.464218
MO 174: 20O - 0.881370 and 13Y - 0.064247
MO 173: 20O - 0.913599 and 13Y - 0.105775
MO 172: 20O - 0.841165 and 12Ag - 0.061001
MO 171: 19O - 0.880029 and 13Y - 0.067762
MO 170: 19O - 0.913905 and 13Y - 0.106459
MO 169: 19O - 0.842712 and 12Ag - 0.047577
MO 168: 18O - 0.908581 and 13Y - 0.094945
MO 167: 18O - 0.888160 and 13Y - 0.062616
MO 166: 18O - 0.841510 and 12Ag - 0.048484
MO 165: 16P - 0.884373 and 12Ag - 0.137267
MO 164: 15P - 0.899136 and 12Ag - 0.124686
MO 163: 14P - 0.891017 and 12Ag - 0.116201
MO 162: 11H - 0.484700 and 8C - 0.508508
MO 161: 10H - 0.489741 and 8C - 0.505408
MO 160: 9H - 0.482529 and 8C - 0.512185
MO 159: 7H - 0.488911 and 4C - 0.508124
MO 158: 6H - 0.487817 and 4C - 0.514804
MO 157: 5H - 0.481643 and 4C - 0.520539
MO 156: 3H - 0.485356 and 0C - 0.516154
MO 155: 2H - 0.486751 and 0C - 0.514228
MO 154: 1H - 0.488693 and 0C - 0.512441
More delocalized orbitals:
MO 392: 49C - 0.223 127C - 0.489 131C - 0.232
MO 391: 52C - 0.240 69C - 0.209 114C - 0.496
MO 390: 34C - 0.173 71C - 0.487 72C - 0.290
MO 389: 62C - 0.201 87C - 0.216 133C - 0.512
MO 388: 21C - 0.520 32C - 0.205 101C - 0.204
MO 387: 54C - 0.208 66C - 0.488 89C - 0.242
MO 386: 22C - 0.172 23C - 0.550 52C - 0.209
MO 385: 29C - 0.557 30C - 0.187 76C - 0.184
MO 384: 28C - 0.258 64C - 0.203 78C - 0.478

```
MO 383:  89C - 0.204 109C - 0.244 111C - 0.491
MO 382:  32C - 0.226 47C - 0.224 60C - 0.495
MO 381:  129C - 0.231 141C - 0.493 153C - 0.220
MO 380:  62C - 0.234 125C - 0.216 138C - 0.495
MO 379:  31C - 0.494 55C - 0.188 76C - 0.265
MO 378:  35C - 0.284 72C - 0.165 113C - 0.493
MO 377:  64C - 0.237 103C - 0.208 136C - 0.492
MO 376:  37C - 0.223 80C - 0.498 153C - 0.222
MO 375:  115C - 0.232 131C - 0.210 134C - 0.498
MO 374:  44C - 0.483 54C - 0.252 109C - 0.201
MO 373:  28C - 0.209 91C - 0.487 103C - 0.243
MO 372:  37C - 0.209 50C - 0.519 129C - 0.202
MO 371:  57C - 0.497 87C - 0.221 125C - 0.227
MO 370:  25C - 0.560 34C - 0.194 35C - 0.170
MO 369:  22C - 0.208 51C - 0.490 69C - 0.247
MO 368:  49C - 0.228 115C - 0.217 151C - 0.495
MO 367:  47C - 0.218 97C - 0.493 101C - 0.234
MO 366:  30C - 0.189 55C - 0.266 68C - 0.492
```

Fuzzy bond order analysis was performed in MultiWFN 3.8 using the orbitals generated by ORCA. ORCA *.gbw files were transformed to molden input files using the orca_2mkl tool included in ORCA 5.0.3.
